# A non-canonical, interferon-independent signaling activity of cGAMP triggers DNA damage response signaling

**DOI:** 10.1038/s41467-021-26240-9

**Published:** 2021-10-27

**Authors:** Daipayan Banerjee, Kurt Langberg, Salar Abbas, Eric Odermatt, Praveen Yerramothu, Martin Volaric, Matthew A. Reidenbach, Kathy J. Krentz, C. Dustin Rubinstein, David L. Brautigan, Tarek Abbas, Bradley D. Gelfand, Jayakrishna Ambati, Nagaraj Kerur

**Affiliations:** 1 Aravind Medical Research Foundation, Madurai, 625020 India; 2 Center for Advanced Vision Science, University of Virginia School of Medicine, Charlottesville, VA USA; 3 Department of Ophthalmology, University of Virginia School of Medicine, Charlottesville, VA USA; 4 Department of Environmental Sciences, University of Virginia, Charlottesville, VA USA; 5 Genome Editing & Animal Models Core, University of Wisconsin Biotechnology Center, Madison, WI USA; 6 Center for Cell Signaling, University of Virginia School of Medicine, Charlottesville, VA USA; 7 Department of Microbiology, Immunology, and Cancer Biology, University of Virginia School of Medicine, Charlottesville, VA USA; 8 Department of Radiation Oncology, University of Virginia, Charlottesville, VA USA; 9 Department of Biomedical Engineering, University of Virginia School of Medicine, Charlottesville, VA USA; 10 Department of Pathology, University of Virginia, Charlottesville, VA USA; 11 Carter Immunology Center, University of Virginia School of Medicine, Charlottesville, VA USA; 12 Department of Ophthalmology and Visual Sciences, The Ohio State University Wexner Medical Center, Columbus, OH USA

**Keywords:** Cell signalling, Interferons, Innate immunity, DNA damage and repair

## Abstract

Cyclic guanosine monophosphate-adenosine monophosphate (cGAMP), produced by cyclic GMP-AMP synthase (cGAS), stimulates the production of type I interferons (IFN). Here we show that cGAMP activates DNA damage response (DDR) signaling independently of its canonical IFN pathways. Loss of cGAS dampens DDR signaling induced by genotoxic insults. Mechanistically, cGAS activates DDR in a STING-TBK1-dependent manner, wherein TBK1 stimulates the autophosphorylation of the DDR kinase ATM, with the consequent activation of the CHK2-p53-p21 signal transduction pathway and the induction of G1 cell cycle arrest. Despite its stimulatory activity on ATM, cGAMP suppresses homology-directed repair (HDR) through the inhibition of polyADP-ribosylation (PARylation), in which cGAMP reduces cellular levels of NAD^+^; meanwhile, restoring NAD^+^ levels abrogates cGAMP-mediated suppression of PARylation and HDR. Finally, we show that cGAMP also activates DDR signaling in invertebrate species lacking IFN (*Crassostrea virginica* and *Nematostella vectensis*), suggesting that the genome surveillance mechanism of cGAS predates metazoan interferon-based immunity.

## Abstract (web summary)

Cyclic guanosine monophosphate-adenosine monophosphate (cGAMP) is normally induced by innate immunity sensing for protection from pathogens. Here the authors show that cGAMP is also upstream of DNA damage signaling by activating ATM-CHK2-mediated repair pathway, while simultaneously suppressing the homology-directed repair.

## Introduction

The ability of eukaryotic cells to detect and appropriately respond to invading nucleic acids in the cytosol is an evolutionarily conserved defense mechanism enabled by an array of pattern recognition receptors (PRRs). Cyclic GMP-AMP synthase (cGAS) is a cytosolic DNA-sensing PRR that triggers downstream signaling pathways by catalyzing the formation of a second messenger, cyclic guanosine monophosphate-adenosine monophosphate (cGAMP). STING, an essential transmembrane adaptor protein, binds to cGAMP and recruits TANK-binding kinase 1 (TBK1) and interferon (IFN) regulatory factor 3 (IRF3) to induce the transcription of IFNs and other cytokines^1,2^.

cGAS is also activated by endogenous double-stranded DNA of nuclear and mitochondrial origin mislocalized to the cytosol^3^. Previous studies have reported that cGAS activation by nuclear DNA occurs in the context of genomic instability and that cGAS–nuclear DNA engagement occurs both in the cytosol^4–9^ and in micronuclei localized outside the primary nuclei^10,11^. More recent work suggests that cGAS accumulates at sites of DNA damage^12^, interferes with homology-directed repair (HDR) independently of its catalytic activity^12,13^, and promotes tumor growth^12^. Although genomic instability triggers an innate immune response that requires cGAS-catalyzed cGAMP activity^4–11^, whether cGAMP signaling in this context impacts the DNA damage response (DDR) pathway is unknown. DDR consists of a coordinated network of signaling pathways that sense DNA damage and promote cellular responses via a set of DNA repair mechanisms, cell survival and death processes, and cell cycle checkpoint pathways. The key apical mammalian DDR signaling components include the protein kinases ATM and ATR, which upon activation in response to aberrant DNA structures influence a multitude of signaling cascades important for DNA replication, DNA repair, and cell cycle control^14^.

Here we identify an unexpected function of cGAS-cGAMP signaling as a mediator of DDR signaling in human, mouse, and invertebrate cells. We show that cGAS-STING signaling promotes DDR signaling activity, resulting in the activation of ATM, G1 cell cycle arrest, and impaired DNA repair via homologous recombination. These data illustrate a previously undiscovered, evolutionarily conserved genome surveillance function of the second messenger cGAMP and introduce new insights into cGAS/cGAMP signaling in an array of biological and therapeutic contexts such as cancer, aging, immunity, cancer therapeutics, and genome editing.

## Results

### cGAMP activates DDR signaling

**Fig. 1:**
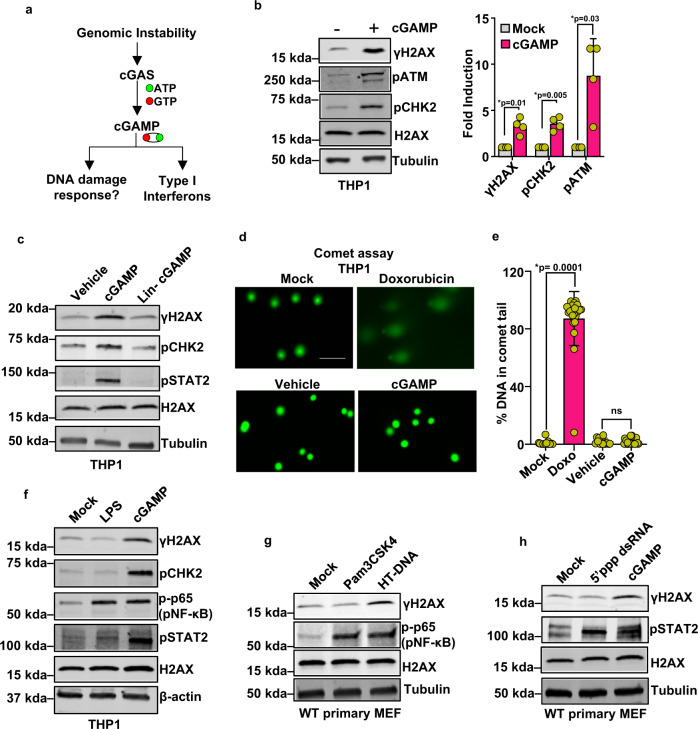
cGAMP activates DNA damage response signaling. **a** Schematic of the proposed hypothesis: the catalysis of cGAMP during genomic instability promotes DNA damage response (DDR). **b** Immunoblots showing the phosphorylation status of DDR signaling proteins H2AX (γH2AX), ATM (pATM), and CHK2 (pCHK2) in THP1 cells stimulated with cGAMP (+) or vehicle (−) for 16 h. Molecular-weight markers (kDa) are indicated to the left of the blots. Quantification of γH2AX, pCHK2, and pATM bands is presented in the bar graph (*n* = 4 independent experiments; data presented are mean ± s.d.; two-tailed paired *t* test; *p < 0.05 indicates significance compared to respective groups; ns indicates not significant). **c** Immunoblots for phosphorylated H2AX (γH2AX), CHK2 (pCHK2), and STAT2 (pSTAT2) in THP1 cells stimulated with vehicle, cGAMP, or signaling incompetent linearized cGAMP (Lin-cGAMP) for 16 h. Bands of interest from representative immunoblots from three independent experiments are shown. **d** Alkaline comet assay was performed to assess DNA damage in THP1 cells that were mock treated, treated with 2 μM doxorubicin, stimulated with vehicle, or stimulated with cGAMP for 16 h. DNA (green) was visualized by staining with Vista Green DNA Dye. While the comet head is composed of intact DNA, the tail consists of genetic fragments and has a length reflective of the amount of DNA damage the cell has sustained. Representative images are presented. Scale bar = 100 μm. **e** Comets with *n* = 28 for Mock, *n* = 23 for Dox, *n* = 15 for vehicle, and *n* = 25 for cGAMP group cells per condition were analyzed using OpenComet; quantification of DNA signal intensity in comet tails as a measure of DNA damage is presented (data presented are mean ± s.d.; two-tailed unpaired *t* test; *p < 0.05 indicates significance compared to respective groups; ns indicates not significant). **f** Immunoblots for γH2AX, pCHK2, pSTAT2, and pNF-κB (p-p65) in THP1 stimulated with LPS (500 ng/ml, 6 h) from *S. minnesota* R595 or cGAMP (16 h). Bands of interest from representative immunoblots from three independent experiments are shown. **g** Immunoblots show phosphorylated H2AX (γH2AX) and NF-κB (p-p65) in WT primary mouse embryonic fibroblasts stimulated with Pam3CSK4 (500 ng/ml, 6 h) or HT-DNA (4 μg/6 well for 6 h). Bands of interest from representative immunoblots from three independent experiments are shown. **h** Immunoblots for γH2AX and pSTAT2 in WT primary MEF transfected with 5’ppp-dsRNA (0.5 μg/6 well for 6 h) or cGAMP (16 h). Total H2AX (H2AX), Tubulin, and/or β-actin were used as loading controls for immunoblots, as indicated. Bands of interest from representative immunoblots from three independent experiments are shown.

Cytosolic DNA triggers cGAS-catalyzed synthesis of the second messenger cGAMP, which subsequently activates IFN signaling. Similarly, nuclear DNA released directly into the cytosol or sequestered in micronuclei following episodes of genomic instability can also be exposed to, and thereby activate, cGAS^4,5,7–11^. We sought to determine whether, in addition to triggering IFN, cGAS-catalyzed cGAMP plays a direct role in genome surveillance (Fig. 1a). To test this, human monocytic THP1 cells were stimulated with cGAMP and the status of DDR signaling was assessed through monitoring the phosphorylation status of key DDR signaling molecules, including histone H2AX (γH2AX), ATM, and CHK2, by immunoblotting. cGAMP treatment of THP1 cells induced phosphorylation of all these proteins (Fig. 1b and Supplementary Fig. 1a). Signaling-incompetent linearized cGAMP (Lin-cGAMP), by contrast, was unable to activate DDR signaling (Fig. 1c). Importantly, cGAMP-induced activation of the DDR was not a consequence of DNA double-strand breaks (DSBs), as no strand breaks were observed in the standard comet assays^15,16^ (Fig. 1d, e).

Activation of innate immune responses relies on the recognition of evolutionarily conserved patterns characteristic of invading pathogens, termed pathogen-associated molecular patterns (PAMPs), which are recognized by PRRs, such as Toll-like receptors (TLRs) and cytosolic DNA/RNA sensors. In order to assess whether DDR activation is unique to cGAMP or represents a generic response induced by PRRs in general, we challenged THP1 or primary mouse embryonic fibroblast (MEF) cells with PAMPs that have the capacity to activate both IFN-dependent and IFN-independent innate immune responses, including the cGAS agonist herring testes (HT) DNA, TLR4 agonist lipopolysaccharide (LPS), the TLR2/TLR1 agonist synthetic triacylated lipopeptide (Pam3CSK4), and the RIG-I agonist 5’PPP-dsRNA. As expected, all these PAMPs activated their characteristic downstream signaling molecules such as nuclear factor-κB and signal transducer and activator of transcription factor 2 (STAT2; Fig. 1f–h). However, whereas cGAMP and the cGAS ligand HT-DNA activated the DDR, the other PAMPs (LPS, Pam3CSK4, and 5’PPP-dsRNA) failed to stimulate the DDR (Fig. 1f–h). Collectively, these studies show that cGAMP induces DDR signaling without triggering DNA strand breaks and that this response is not a general consequence of PRR activation.

### cGAMP-induced DDR activation requires STING and TBK1 but operates independently of IFN signaling

**Fig. 2:**
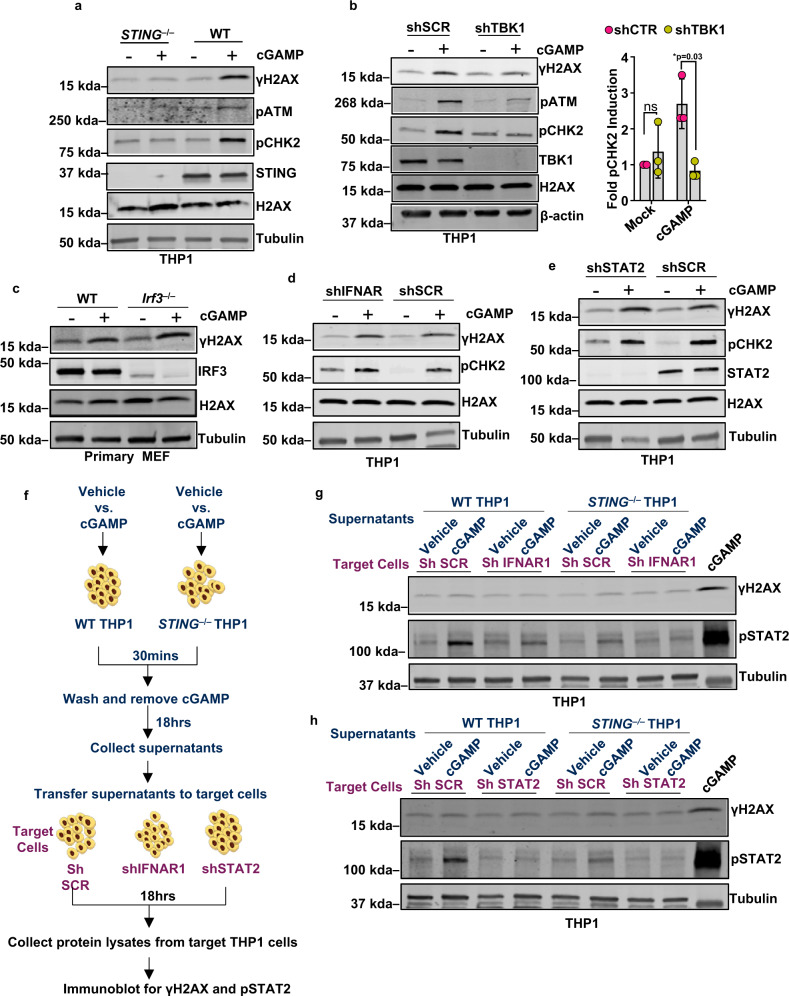
cGAMP-driven DDR signaling requires STING and TBK1 but operates independently of interferon signaling. **a** Immunoblots for phosphorylated H2AX (γH2AX), ATM (pATM), CHK2 (pCHK2), and STING in WT and *STING*^−/−^ THP1 cells treated with vehicle (−) or cGAMP (+) for 16 h. Bands of interest from representative immunoblots from three independent experiments are shown. **b** Immunoblots for γH2AX, pATM, pCHK2, and TBK1 in control (shSCR) or TBK1 shRNA knockdown (shTBK1) THP1 cells stimulated with vehicle (−) or cGAMP (+) for 16 h. Quantification of pCHK2 bands is presented in the bar graph (*n* = 3 independent experiments; data presented are mean ± s.d.; two-tailed unpaired *t* test *p < 0.05 indicates significance compared to respective groups; ns indicates not significant). **c** Immunoblots for phosphorylated H2AX (γH2AX) and total IRF3 in WT and *Irf3*^−/−^ primary mouse embryonic fibroblasts mock transfected (−) or transfected with cGAMP (+) for 16 h. Bands of interest from representative immunoblots from three independent experiments are shown. **d** Immunoblots for phosphorylated H2AX (γH2AX) and CHK2 (pCHK2) in control (shSCR) or shIFNAR1 knockdown THP1 cells stimulated with cGAMP (+) or vehicle (−) for 16 h. Bands of interest from representative immunoblots from three independent experiments are shown. **e** Immunoblots for phosphorylated H2AX (γH2AX), CHK2 (pCHK2), and STAT2 in control (shSCR) or shSTAT2 knockdown THP1 cells stimulated with cGAMP (+) or vehicle (−) for 16 h. Bands of interest from representative immunoblots from three independent experiments are shown. **f** Schematic of the experimental design employed to test whether cGAMP-induced paracrine signaling mediates activation of DDR. Supernatants from vehicle- or cGAMP-stimulated WT or *STING*^−/−^ THP1 cells were collected after 18 h and added to target THP1 cells: shSCR, shIFNAR1, and shSTAT2 for 18 h. These cultures were analyzed by immunoblotting for γH2AX and pSTAT2. **g** Immunoblots for γH2AX and pSTAT2 in target THP1 cells (shSCR and shIFNAR1) incubated with conditioned media from vehicle- or cGAMP-stimulated WT or *STING*^*−/−*^ THP1 cells (experimental design described in **f**). Bands of interest from representative immunoblots from three independent experiments are shown. **h** Immunoblots for γH2AX and pSTAT2 in target THP1 cells (shSCR and shSTAT2) incubated with conditioned media from vehicle- or cGAMP-stimulated WT or *STING*^*−/−*^ THP1 cells (experimental design described in **f**). Lysates from cGAMP-stimulated wild type THP1 cells were run alongside test samples as positive controls in **g**, **h**. Total H2AX (H2AX), Tubulin, and/or β-actin were used as loading controls for immunoblots as indicated. Bands of interest from representative immunoblots from three independent experiments are shown.

Since canonical cGAMP signaling associated with IFN induction is mediated via the binding of cGAMP to the adaptor protein STING^17^, we tested whether STING is involved in cGAMP-induced DDR activation. Activation of DDR by cGAMP, as indicated by the phosphorylation status of H2AX, ATM, and ATM substrate CHK2, was abrogated by the deletion of STING in THP1 cells and in primary MEFs (Fig. 2a and Supplementary Fig. 1b). Furthermore, short hairpin RNA (shRNA)-mediated knockdown of TBK1, which is necessary for cGAMP-mediated IFN induction^18,19^, substantially reduced cGAMP-induced DDR signaling in THP1 cells (Fig. 2b). We conclude that cGAMP induces DDR signaling via STING and TBK1. The induction of IFN genes in our experimental conditions using exogenous cGAMP stimulation was comparable to that of cytosolic DNA-induced, endogenously produced cGAMP (Supplementary Fig. 1c)

Once activated by cGAS-STING, TBK1 proceeds to phosphorylate the transcription factor IRF3, which subsequently translocates to the nucleus to drive the expression of type I IFNs. We therefore evaluated the potential involvement of IRF3 in cGAMP-mediated DDR signaling by challenging wild-type (WT) and *Irf3*^–/–^ primary with the cGAS second messenger cGAMP. Notably, activation of DDR marker γH2AX proceeded normally in response to cGAMP in *Irf3*^–/–^ primary MEFs (Fig. 2c). Cells treated with human recombinant IFN β (recIFN-β), a primary cytokine induced by cGAMP via IRF3^1^, demonstrated activation of the downstream signaling molecule STAT2 but not the DDR marker γH2AX (Supplementary Fig. 2a). Binding of type I IFN to IFN-α/β receptor (IFNAR) triggers downstream signaling via a STAT2-containing transcriptional factor complex, which consequently induces IFN-stimulated genes^20^. Knockdown of IFNAR1 (Supplementary Fig. 2b, c) and STAT2 (Supplementary Fig. 2d) in THP1 cells failed to impede cGAMP-mediated induction of γH2AX and pCHK2 (Fig. 2d, e). Consistent with this observation, cGAMP-induced H2AX phosphorylation was unimpaired in *Ifnar1*^*–/–*^ or in *Stat2*^*–/–*^ primary MEFs (Supplementary Fig. 2e, f). Next, to assess the potential role of cGAMP-induced cytokines in DDR activation, we treated WT and STING-deficient THP1 cells with cGAMP and collected conditioned media from the cultures after 18 h. Target IFNAR1 and STAT2 knockdown THP1 cells (vs. scramble shRNA THP1) were incubated with the conditioned media from cGAMP-treated cells and probed for γH2AX (Fig. 2f). As expected, control (shSCR) but not shIFNAR1/shSTAT2 target cells exhibited elevated levels of pSTAT2 when treated with supernatant from WT but not from *STING*^*−/−*^ THP1 (Fig. 2g, h; middle blot). Despite activating their IFNAR/STAT2 signaling pathways, conditioned media from cGAMP-treated cells failed to induce γH2AX (Fig. 2g, h; top blot). Collectively, these results indicate that DDR signaling induction by cGAMP requires STING and TBK1 but proceeds independently of the IRF3-IFNβ-IFNAR-STAT2 signaling axis and paracrine signaling.

### cGAS-cGAMP-STING-TBK1 signaling axis promotes DDR signaling induced by genotoxic agents

**Fig. 3:**
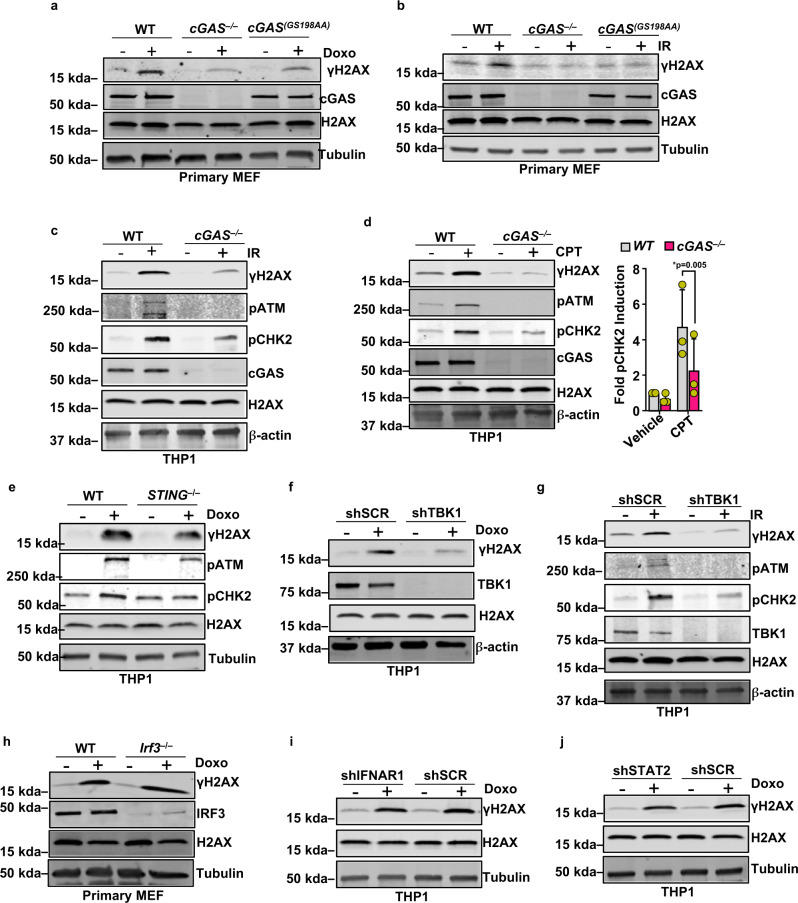
cGAS-cGAMP-STING-TBK1 signaling axis promotes DDR signaling induced by genotoxic agents. **a**, **b** Immunoblots for γH2AX and cGAS in whole-cell lysates collected from WT, *cGAS*^*–/–*^, and catalytically inactive mutant *cGAS*^*(GS198AA)*^ primary MEF cultures mock treated (−) or treated with doxorubicin (0.5 μM for 2 h) or ionizing radiation (5 Gy for 1 h) (+). Bands of interest from representative immunoblots from three independent experiments are shown. **c**, **d** Immunoblots for γH2AX, pATM, pCHK2, and cGAS in WT and *cGAS*^−/−^ THP1 upon mock treatment (−) and ionizing radiation (5 Gy for 1 h) or camptothecin (2 μM for 4 h) (+) respectively. Quantification of pCHK2 bands is presented in the bar graph (*n* = 3 independent experiments; data presented are mean ± s.d.; two-tailed paired *t* test; *p < 0.05 indicates significance compared to respective groups; ns indicates not significant). Bands of interest from representative immunoblots from three independent experiments are shown. **e** Immunoblots for γH2AX, pATM, and pCHK2 in WT and *STING*^−/−^ THP1 following Doxorubicin treatment (1 μM for 16 h). **f** Immunoblots for γH2AX and TBK1 in control (shSCR) or shTBK1 knockdown THP1 cells stimulated with Doxorubicin (1 μM for 16 h). Bands of interest from representative immunoblots from three independent experiments are shown. **g** Immunoblots for γH2AX, pATM, pCHK2, and TBK1 in control (shSCR) or shTBK1 knockdown THP1 cells following ionizing radiation (+) (5 Gy for 1 h). Bands of interest from representative immunoblots from three independent experiments are shown. **h** Immunoblots for γH2AX and IRF3 in WT and *Irf3*^−/−^ primary MEF cells mock treated (−) or treated with doxorubicin (+) (0.5 μM for 2 h). Bands of interest from representative immunoblots from three independent experiments are shown. **i**, **j** Immunoblots for γH2AX in whole-cell lysates from shScrambled (shSCR) and shIFNAR1 (**i**) or shSTAT2 (**j**) THP1 cells mock treated (−) or treated with doxorubicin (+) (1 μM for 16 h). Total H2AX (H2AX), Tubulin, and/or β-actin were used as loading controls for immunoblots as indicated. Bands of interest from representative immunoblots from three independent experiments are shown.

**Fig. 4:**
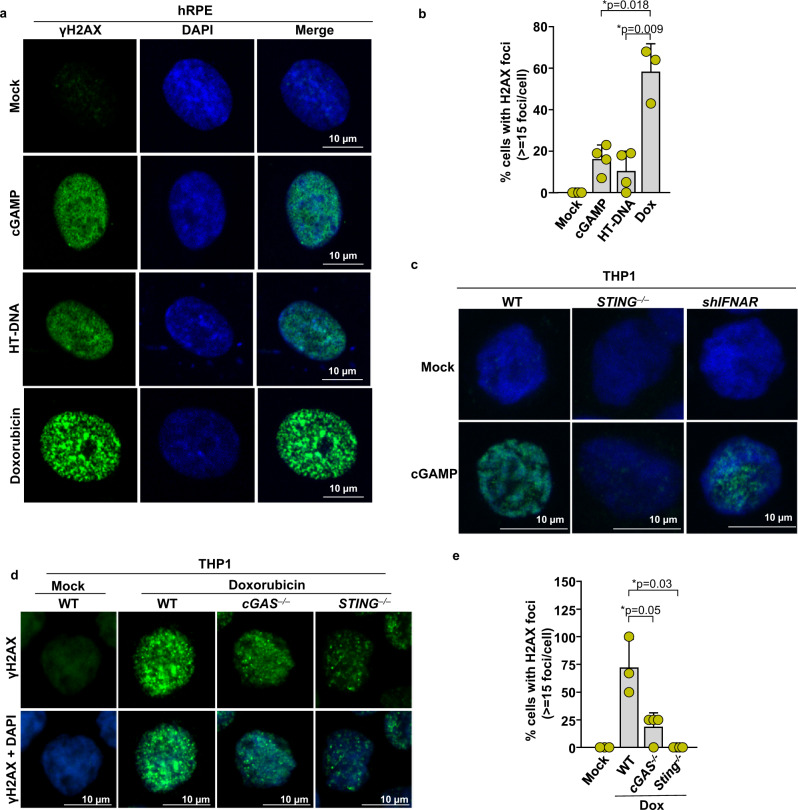
cGAMP does not induce γH2AX foci formation. **a** Immunofluorescence of γH2AX in human RPE cells mock treated, exposed to doxorubicin for 2 h, transfected with cGAMP, or incubated with HT-DNA for 6 h, scale bar = 10 μm. Bands of interest from representative immunoblots from three independent experiments are shown. **b** Quantification of γH2AX cells, each data point represents the percentage of cells with ≥5 foci in a microscopic field (*n* = 4 number of fields each condition, data presented are mean ± s.d.; two-tailed, unpaired *t* test; *p < 0.025 indicates significance compared to respective groups; ns indicates not significant; adjustments are made for multiple comparisons). **c** Immunofluorescence of γH2AX in WT, *STING*^−/−^, or shIFNAR THP1 cells stimulated with cGAMP for 16 h, scale bar = 10 μm. Representative images from three independent biological replicates are shown. **d** Immunofluorescence of γH2AX in WT, *STING*^−/−^, or cGAS^−/−^ THP1 cells mock treated (WT only) or treated with doxorubicin, scale bar = 10 μm. **e** Quantification of γH2AX cells, each data point represents the percentage of cells with ≥5 foci in a microscopic field (*n* = 4 number of fields each condition, data presented are mean ± s.d.; two-tailed unpaired *t* test; *p < 0.025 indicates significance compared to respective groups; ns indicates not significant; adjustments are made for multiple comparisons).

Our observations demonstrating activation of DDR signaling by cGAMP prompted us to test whether cGAS-cGAMP signaling contributes to the activation of DDR signaling following genotoxic insults. Consistent with earlier reports that cGAS-driven innate immune signaling is activated in response to DNA damage^4–11^, THP1 cells exposed to doxorubicin (dox) reacted by producing cGAMP (Supplementary Fig. 3a). We then assessed DDR signaling activity in primary *cGAS*^*−/−*^ MEFs and in MEFs harvested from a new catalytically inactive mutant cGAS mouse model (*cGAS*^*(GS198AA)*^) created by us, in which amino acid residues Gly198 and Ser199 in the mouse cGAS catalytic domain^1^ are mutated to Ala (Supplementary Fig. 3b). DDR signaling was suppressed both in *cGAS*^*−/−*^ and *cGAS*^*(GS198AA)*^ primary MEFs exposed to dox, ionizing radiation (IR), or camptothecin (CPT) (Fig. 3a, b and Supplementary Fig. 3c). Furthermore, the phosphorylation of H2AX, ATM, and CHK2 in response to IR and CPT were all suppressed in cGAS^*−/−*^ human THP1 cells (Fig. 3c, d). Just as with cGAMP (Fig. 2), DDR induction by dox, IR, or CPT was abrogated in cells lacking STING (Fig. 3e and Supplementary Fig. 3d–f) or TBK1 (Fig. 3f, g) but not in cells lacking IRF3, IFNAR, or STAT2 (Fig. 3h–j). Collectively, these findings suggest that cGAS-cGAMP signaling plays a critical role in promoting DDR signaling in response to genotoxic insults and that this novel activity of cGAS-cGAMP is mediated via STING and TBK1 but operates independently of its other downstream canonical IFN signaling pathway. Although cGAMP stimulation induced DDR signaling as demonstrated by H2AX, ATM, and CHK2 phosphorylation, no strand breaks were observed in the comet assay (Fig. 1d, e). Interestingly, whereas cGAMP treatment, as well as treatment with HT-DNA, yielded γH2AX marked by pan-nuclear staining, exposure to dox catalyzed the formation of γH2AX foci characteristic of DSB induction (Fig. 4a, b). As expected from our biochemical analysis of *STING*^*−/−*^ cells detailed above (Fig. 3e and Supplementary Fig. 3d–f), cGAMP-induced pan-nuclear γH2AX staining was suppressed in *STING*^*–/–*^ but not in shIFNAR THP1 cells (Fig. 4c). Furthermore, deficiency of cGAS and STING in dox-treated cells resulted in a significant reduction in the intensity of γH2AX foci (Fig. 4d, e). Collectively these findings demonstrate that although cGAS-cGAMP signaling induces phosphorylation of H2AX, it does not directly contribute to the formation of γH2AX foci, but rather it mimics DDR signaling without requiring strand breaks as well as amplifies DDR signaling induced by DNA damage.

### TBK1 kinase activity stimulates ATM autophosphorylation

**Fig. 5:**
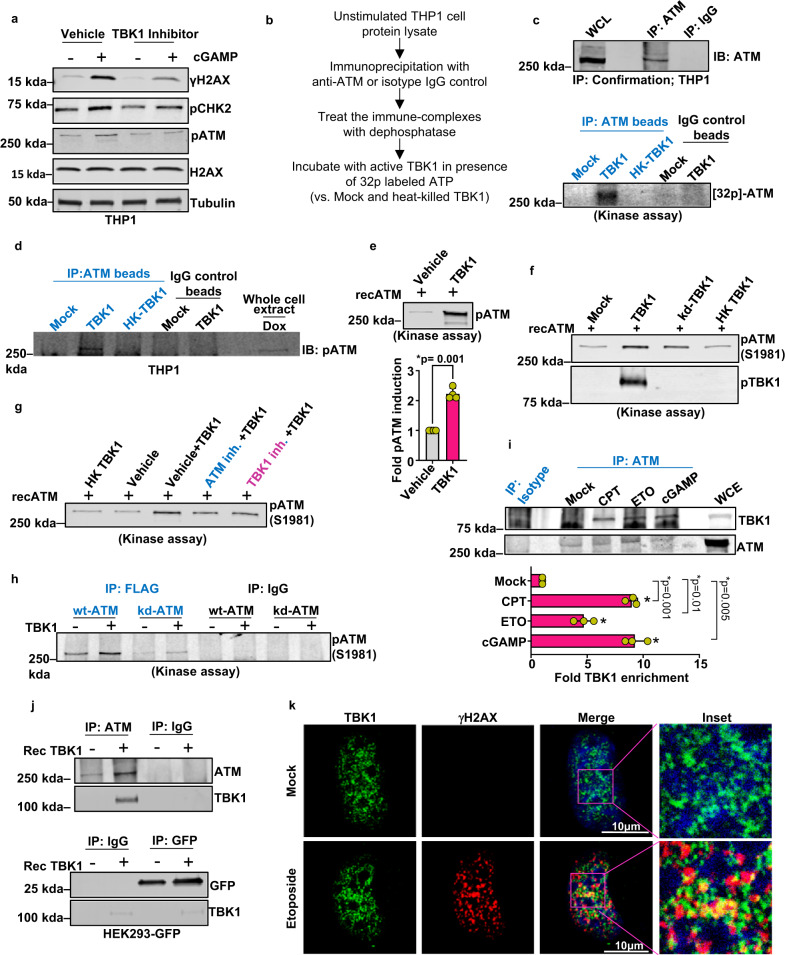
cGAS-cGAMP-induced TBK1 kinase activity stimulates ATM autophosphorylation. **a** Immunoblots for γH2AX, pCHK2, and pATM in THP1 cells pretreated with vehicle or 2 μM TBK1 inhibitor (MRT67307) and then stimulated with cGAMP (+) or vehicle (−) for 16 h. Bands of interest from representative immunoblots from three independent experiments are shown. **b** Schematic of TBK1 kinase assay. **c** Endogenous ATM in WT-THP1 cells that were pulled down using immunoprecipitation and used as substrates in kinase assays performed with a recombinant TBK1 protein and radiolabeled γ-ATP. Upper panel shows immunoblot of immunoprecipitated ATM. Lower panel shows autoradiogram of ^32^P incorporated into beads bound to endogenous ATM. Bands of interest from representative immunoblots from three independent experiments are shown. **d** Immunoblot showing phosphorylated ATM from the kinase assay reaction using Phospho-ATM (Ser1981) (IP: ATM beads) antibody. Bands of interest from representative immunoblots from three independent experiments are shown. **e** Immunoblot showing phosphorylated ATM from the kinase assay reaction with recombinant ATM and TBK1 proteins. The immunoblotting was carried out using Phospho-ATM (Ser1981) antibody. Quantification of pATM bands is presented in the bar graph (*n* = 4 independent experiments; data presented are mean ± s.d.; two-tailed, paired *t* test; *p < 0.05 indicates significance compared to respective groups; ns indicates not significant). **f** Immunoblot showing phosphorylated ATM from the kinase assay reaction using recombinant ATM and catalytically active TBK1, kinase-dead TBK1 (kd-TBK1), or heat-killed TBK1 (HK TBK1) as indicated. Bands of interest from representative immunoblots from three independent experiments are shown. **g** Immunoblot showing phosphorylated ATM from the kinase assay reaction using recombinant ATM and TBK1 in the presence of inhibitors of ATM or TBK1, as indicated. Bands of interest from representative immunoblots from three independent experiments are shown. **h** Immunoblot showing phosphorylated ATM from the kinase assay reaction entailing incubation of wild-type (wt) or catalytically dead (kd) ATM with recombinant TBK1. Bands of interest from representative immunoblots from three independent experiments are shown. **i** Immunoblot showing TBK1 enrichment in ATM immunoprecipitate in cells mock treated or treated with CPT 5 μM, etoposide (ETO) 10 μM, or 2 μg cGAMP for 16 h each. WCE is the whole-cell extract. Quantification of pATM bands is presented in the bar graph (*n* = 3 independent experiments; data presented are mean ± s.d.; *p < 0.0.016, two-tailed paired *t* test; ns = not significant; adjustments are made for multiple comparisons). **j** Interaction of ATM and TBK1 shown by Co-IP analysis. ATM and GFP were immunoprecipitated from GFP-positive HEK293 cells using target-specific or isotype antibodies. The resulting bead-bound ATM and GFP complexes were incubated with recombinant TBK1. The beads with immune complexes were washed and immunoblotted to examine for the presence of TBK1. TBK1 was found in complex with ATM but not with GFP. Bands of interest from representative immunoblots from three independent experiments are shown. **k** Immunofluorescence imaging of γH2AX and TBK1 in U2OS-STING cells mock treated or treated with etoposide 10 μM for 16 h. Representative images from three independent biological replicates are shown.

TBK1 is a serine/threonine kinase whose activity is mediated by formation of distinct complexes, the composition of which are dictated by cellular stimuli and cell type^21–23^. Because DDR activation by cGAMP and genotoxic insults is dependent on TBK1 (Figs. 2b and 3f, g), we investigated the role of TBK1 kinase activity in DDR signaling. Pretreatment of THP1 cells with the TBK1-specific inhibitor MRT67307^24,25^ suppressed cGAMP-induced phosphorylation of ATM (S1981), CHK2, and H2AX (Fig. 5a). ATM is activated through dimerization-induced autophosphorylation at Ser1981, a canonical SQ/TQ motifs, the phosphorylation of which can only be catalyzed by members of the phosphatidylinositol 3-kinase-like family of protein kinases (e.g., ATM, ATR, and DNA-PKcs)^26–28^, with the subsequent release of the active phosphorylated monomers^29,30^. Therefore, employing a standard kinase assay (Fig. 5b), we tested whether TBK1 directly impacts ATM phosphorylation. We immunopurified ATM from THP1 cells (Fig. 5c—top panel) and incubated the immunoprecipitates with recombinant, catalytically active TBK1 in a standard kinase reaction with radiolabeled ATP (Fig. 5b, c—lower panel). Whereas the immunoprecipitated ATM incubated with recombinant TBK1 became phosphorylated, the ATM incubated with heat-killed TBK1 (TBK1-HK), and immunoprecipitation complexes resulting from an isotype control IgG antibody incubated with the active TBK1, did not reveal a band corresponding to phosphorylated ATM incubation (Fig. 5c—lower panel).

Additional experiments revealed that: (a) the recombinant TBK1 promoted the phosphorylation of immunopurified or recombinant ATM specifically on Ser1981 (Fig. 5d, e), and this is augmented by TBK1 catalytic activity (Fig. 5f, g), (b) ATM phosphorylation on Ser1981 in these reactions was suppressed not only by the TBK1 inhibitor (or heat inactivation of the enzyme) but also by the ATM-specific inhibitor KU55933 (Fig. 5g), and (c) TBK1 did not significantly enhance the phosphorylation of catalytically inactive ATM (Fig. 5h). Based on these findings, we conclude that TBK1, through its catalytic activity, promotes ATM autophosphorylation at Ser1981, but it does not directly phosphorylate ATM on Ser1981.

How TBK1 stimulates ATM autophosphorylation remains to be fully understood but likely involves direct contact between these two protein kinases. In support of this hypothesis, co-immunoprecipitation studies demonstrated TBK1 enrichment in the ATM-bound fractions in cells exposed to DNA-damaging agents or cGAMP (Fig. 5i). Furthermore, recombinant TBK1 co-precipitated with the immunoprecipitated ATM but not with control green fluorescent protein (GFP) protein (Fig. 5j). Immunofluorescence imaging further revealed a modest level of superimposition of TBK1 with γH2AX in cells exposed to the DNA-damaging agent etoposide (Fig. 5k).

Collectively our findings identify that cGAS-cGAMP-STING-activated TBK1 kinase activity stimulates ATM autophosphorylation during DDR signaling, potentially through enhancing ATM dimerization or conformational changes induced through interactions with TBK1.

### cGAMP signaling does not promote nuclear localization of cGAS

**Fig. 6:**
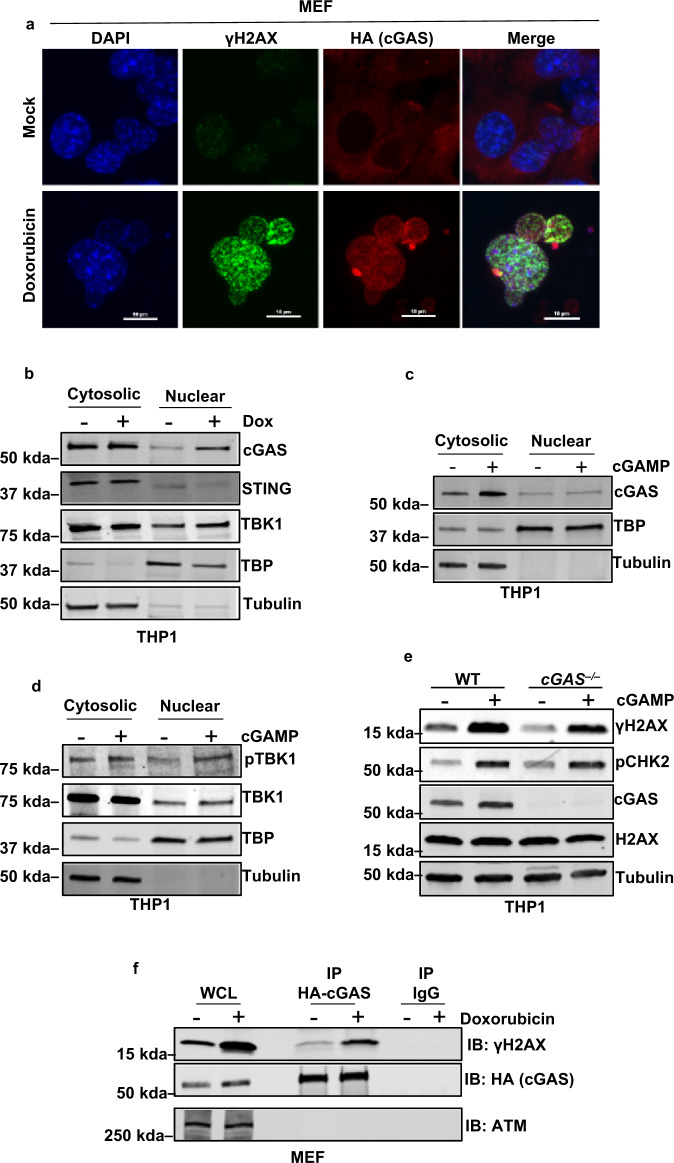
Doxorubicin treatment but not cGAMP signaling promotes nuclear cGAS localization. **a** Immunofluorescence of HA-cGAS and γH2AX in MEF cells mock treated or exposed to doxorubicin (2 μM for 6 h), scale bar = 10 μm. Representative images from three independent biological replicates are shown. **b** Immunoblots for endogenous cGAS, STING, and TBK1 in the cytoplasmic and nuclear fractions of THP1 cells after mock treatment (−) or treatment with doxorubicin (+) (2 μM for 6 h). Tubulin and TBP served as cytoplasmic and nuclear loading controls, respectively. Bands of interest from representative immunoblots from three independent experiments are shown. **c** Immunoblots for endogenous cGAS in the cytoplasmic and nuclear fractions of THP1 cells after mock treatment (−) or treatment with cGAMP (+) for 16 h. Tubulin and TBP served as cytoplasmic and nuclear loading controls, respectively. Bands of interest from representative immunoblots from three independent experiments are shown. **d** Immunoblots for phosphorylated and total endogenous TBK1 in the cytoplasmic and nuclear fractions of THP1 cells after mock treatment (−) or treatment with cGAMP (+) for 16 h. Tubulin and TBP served as cytosolic and nuclear loading controls, respectively. Bands of interest from representative immunoblots from three independent experiments are shown. **e** Immunoblots for DDR signaling proteins H2AX (γH2AX), phosphorylated CHK2 (pCHK2), and endogenous cGAS in WT and *cGAS*^−/−^ THP1 cells after treating with mock (−) or cGAMP (+) for 16 h. Total H2AX (H2AX) and tubulin serve as internal controls. Bands of interest from representative immunoblots from three independent experiments are shown. **f** Immunoblot (IB) for γH2AX, ATM, and HA-cGAS of anti-HA or anti-IgG immunoprecipitates (IP) from HA-cGAS-reconstituted *cGAS*^−/−^ immortalized MEF’s in the presence (+) or absence (−) of doxorubicin (2 μM for 6 h). γH2AX but not ATM was enriched in the cGAS immunoprecipitate. Bands of interest from representative immunoblots from three independent experiments are shown.

Although originally described to be a cytosolic DNA sensor, multiple recent studies highlight rather promiscuous cGAS subcellular localization in various compartments, including the nucleus, cytoplasm, and plasma membrane^13,31–34^ (elaborated in the “Discussion” section). Lui et al.^12^ reported that, in response to DNA damage, cGAS translocates to the nucleus and accumulates at sites of DNA damage where it directly interacts with γH2AX. In accordance with these findings, we observed nuclear enrichment of cGAS in response to dox-induced genotoxicity (Fig. 6a, b). Furthermore, we found that among the cGAS-cGAMP downstream signaling components, dox treatment promoted nuclear enrichment of TBK1 but not of STING (Fig. 6b). Since cGAMP activates DDR signaling, we wondered whether cGAMP stimulation, like dox, also induces nuclear translocation of cGAS and its signaling component, TBK1. cGAMP stimulation triggered nuclear enrichment of TBK1 and phosphorylated TBK1 (pTBK1); however, no nuclear enrichment of cGAS was observed in cGAMP-stimulated cells (Fig. 6c, d). Additionally, cGAMP-induced DDR signaling proceeded normally in cGAS-deficient cells (Fig. 6e). These findings suggest that cGAMP induces DDR signaling without provoking cGAS nuclear translocation. Additionally, and consistent with recent reports^12,13^ in dox-treated cells, nuclear translocated cGAS was found in a complex with γH2AX as revealed by co-immunoprecipitation assay (Fig. 6f). Based on the existence of these cGAS-γH2AX assemblages, we examined whether cGAS also associates with other DDR signaling proteins, namely, ATM, but we detected no such interactions following DNA damage (Fig. 6f). These results collectively suggest that, although cGAMP can promote DDR signaling through ATM phosphorylation and activation, it does not contribute to nuclear enrichment of cGAS, which arises specifically in response to bona fide DNA damage. These findings, in conjunction with our data demonstrating: (1) activation of DDR signaling by cGAMP, (2) suppressed DDR signaling in catalytically null cGAS mutant MEFs, (3) nuclear translocation of pTBK1 in dox- and cGAMP-stimulated cells, and (4) TBK1-mediated ATM phosphorylation, suggest that cGAS-cGAMP-STING-activated TBK1 kinase activity induces ATM autophosphorylation and consequent DDR signaling.

### cGAMP signaling induces G1 cell cycle arrest

**Fig. 7:**
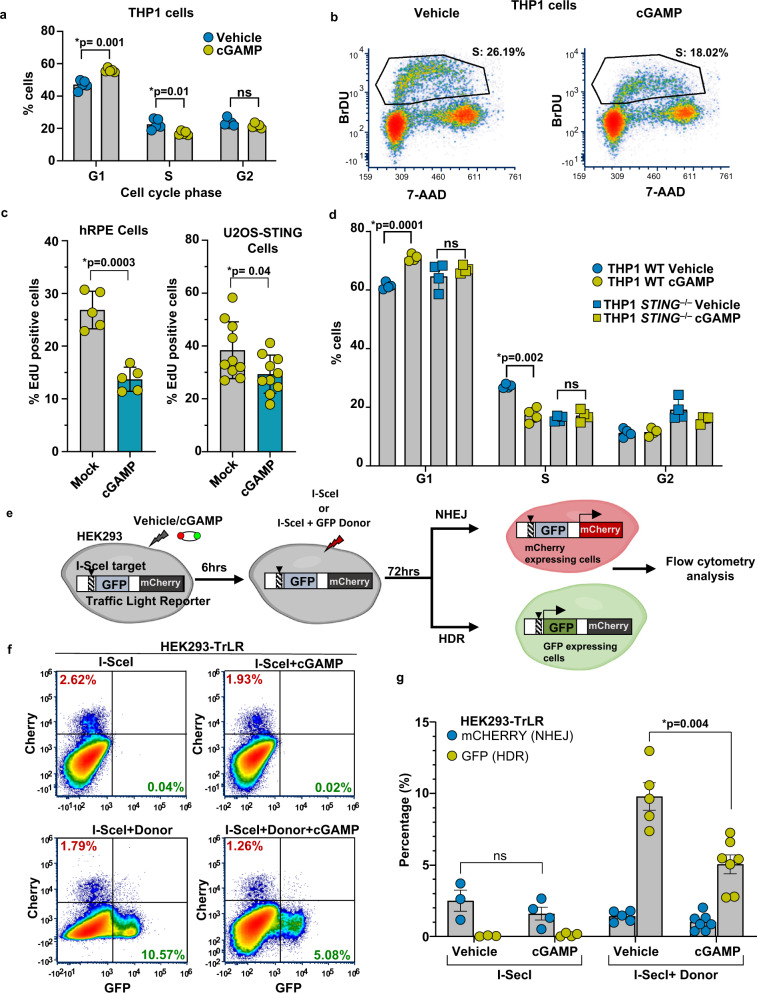
cGAMP signaling induces G1 arrest and HDR suppression. **a** The distribution of WT THP1 cells in the G1, S, and G2 cell cycle phases (BrdU-FITC positivity) 24 h after stimulation with vehicle or cGAMP (*n* = 5 independent experiments; data presented are mean ± s.d.; two-tailed unpaired *t* test; *p < 0.05 indicates significance compared to respective groups; ns indicates not significant). Bromodeoxyuridine (BrdU) was added to label cells for 1 h before harvesting. Fixed cells were stained with FITC-conjugated anti-BrdU antibody and 7-AAD for total DNA content. The percentage of cells in each cell cycle phrase is shown; 20,000 cells were counted for FACS analysis. **b** Representative cell cycle dot plots of WT THP1 cells stimulated with vehicle or cGAMP. **c** Cell proliferation in human RPE cells and U2OS-STING cells after vehicle or cGAMP treatment (18 h) is measured by EdU incorporation. Cells incubated with EdU for 1 h prior to harvesting were stained for EdU incorporation using a Click-iT EdU assay. The percentages of cells with incorporated EdU as visualized by confocal microscopy are indicated in the graph. Each data point represents the percentage of cells in one image field (*n* = 5 fields with over 100 cells collectively per condition for hRPE cells; *n* = 10 fields with over 200 cells collectively per condition for U2OS-STING cells; data presented are mean ± s.d.; two-tailed unpaired *t* test; *p < 0.05 indicates significance compared to respective groups; ns indicates not significant). **d** Cell cycle analysis (propidium iodide stain) of WT and *STING*^−/−^ THP1 cells, 24 h after stimulation with cGAMP or vehicle (*n* = 4 independent experiments; data presented are mean ± s.d.; two-tailed unpaired *t* test; *p < 0.05 indicates significance compared to respective groups; ns indicates not significant). **e** Schematic of the experimental design utilizing a Traffic Light Reporter (TrLR) system in HEK293 cells employed to monitor DSB repair by non-homologous end joining (NHEJ) and HDR. HEK293 cells with stably integrated TrLR (HEK293-TrLR) were mock stimulated or stimulated via cGAMP transfection. Six hours post cGAMP transfection, DSBs were induced via enforced expression of the endonuclease I-SceI with or without GFP donor repair template. Seventy-two hours later, cells were trypsinized and analyzed by flow cytometry for mCherry+ or GFP+ fluorescence, indicative of NHEJ or HDR at the reporter locus, respectively. **f** Flow cytometric analysis of HEK293-TrLR cells transfected with vehicle/cGAMP expressing I-SceI only or I-SceI with donor. Representative graphs from *n* = 3 independent experiments are presented. **g** Quantification of data from **f** is presented (*n* = 3 Vehicle+I-SceI, *n* = 4 cGAMP+I-SceI, *n* = 5 Vehicle+I-SceI+Donor, and *n* = 7 cGAMP+I-SceI+Donor, Samples are from independent experiments; data presented are mean ± s.e.m.; two-tailed unpaired *t* test; *p < 0.05 indicates significance compared to respective groups; ns indicates not significant).

Our observation that cGAMP triggers phosphorylation of CHK2, a key component of DDR whose activation in response to genotoxic stress triggers G1 cell cycle arrest^35,36^, prompted us to investigate the effect of cGAMP on cell cycle progression. Employing a bromodeoxyuridine (BrdU) incorporation-based cell cycle analysis approach, we found that treatment of THP1 cells with cGAMP substantially increased the proportion of cells arrested in the G1 phase and simultaneously decreased the number of cells in the S phase (Fig. 7a, b). Consistent with these data, cGAMP also inhibited THP1 cell proliferation (Supplementary Fig. 4a). We then utilized a 5-ethynyl-2’-deoxyuridine (EdU) incorporation assay to quantify the effect of cGAMP on the abundance of S phase cells in two other cell types—human primary retinal pigment epithelium (hRPE) and human osteosarcoma U2OS cells. Since U2OS cells were found to express low levels of STING (Supplementary Fig. 4b, upper panel), they were reconstituted with a human STING expression cassette via lentiviral transduction (Supplementary Fig. 4c, upper panel). When stimulated with cGAMP, these STING-reconstituted U2OS (STING-U2OS) cells responded by exhibiting biochemical evidence of DDR signaling (Supplementary Fig. 4c, lower panel). Consistent with the cGAMP-induced reduction in the number of THP1 cells in the S phase, our EdU incorporation assay revealed a diminished count of hRPE and STING-U2OS EdU-positive cells under cGAMP-stimulated conditions (Fig. 7c). Unlike cGAMP, however, the signaling-incompetent Lin-cGAMP did not affect cell cycle progression (Supplementary Fig. 4d). Furthermore, studies in cells deficient or depleted of STING, TBK1, IFNAR1, and STAT2 demonstrated that cGAMP-induced G1 cell cycle arrest was reliant on the former two signaling proteins (i.e., STING and TBK1) but occurs independently of IFNAR and STAT2 (Fig. 7d and Supplementary Fig. 4e–g). This pattern mirrors the selective dependency of cGAS/cGAMP-driven phosphorylation of H2AX, ATM, and CHK2 on these signaling proteins.

The G1/S transition is tightly controlled by a dynamic protein complex that contains the retinoblastoma (Rb) and the E2F transcription factors^37,38^; while Rb in its hypophosphorylated state binds E2F transcription factors to form an inhibitory complex, it is phosphorylated in cells committed to entering the S phase and thus disassociates from and releases E2F transcription factors to enable transcription of their target genes required for DNA synthesis. Consistent with the G1 arrest induced by cGAMP, cGAMP-stimulated THP1 cells exhibited Rb hypophosphorylation (Supplementary Fig. 5a). This observation was further supported by the decreased transcript abundance of E2F target genes^39,40^ (Supplementary Fig. 5b). To similar effect, a screen assessing the relative transcript abundance of DNA damage response-relevant genes in cGAMP-stimulated cells revealed strong induction of CDKN1A/p21 (Supplementary Fig. 5c), a cell cycle regulator and p53 target critical for genotoxic stress-induced G1 cell cycle arrest^41^. Unsurprisingly, other genes found to be upregulated are also implicated in the mediation of cell cycle progression, either directly as G1 checkpoint regulators or indirectly through interactions with p53 and other key tumor-suppressor factors (Supplementary Fig. 5c). Importantly, the induction of these genes by cGAMP was dependent on STING and TBK1 (Supplementary Fig. 6a, b). Furthermore, shRNA-mediated knockdown of p53 abrogated the cGAMP-induced G1 arrest (Supplementary Fig. 7a, b). Similarly, the blockade of ATM either with small molecule inhibitors or shRNA-mediated knockdown prevented cGAMP from inducing G1 arrest (Supplementary Fig. 7c, d). These findings collectively suggest that cGAMP-driven G1 arrest proceeds through the activation of the ATM-CHK2-p53 signal transduction axis and the consequent induction of p21 and CDK2 inhibition, resulting in Rb hypophosphorylation and the suppression of E2F-dependent gene expression and cell cycle progression.

### cGAMP impairs the efficiency of HDR in human cells

DNA DSBs are primarily repaired either by error-prone non-homologous end joining (NHEJ) or error-free HDR. The latter proceeds via strand invasion and relies upon homologous DNA templates for sequence reconstruction and is thus a more accurate mode of DSB repair that is employed primarily in the S and G2 phases of the cell cycle^42^. Given our observation that cGAMP inhibits G1/S phase cell cycle progression, we examined whether cGAMP signaling impairs HDR using a Traffic Light Reporter (TrLR) assay that permits concurrent fluorescent measurement of NHEJ and HDR following the expression of a site-specific endonuclease (Fig. 7e)^43^. Using lentivirus, a TrLR reporter plasmid was integrated into HEK293 (HEK293-TrLR). HEK293 cells, which intrinsically lack cGAS expression^44,45^, do express STING and respond normally to cGAMP stimulation as revealed by the induced phosphorylation status of H2AX, STING, and STAT2 (Supplementary Fig. 8a, b). HEK293-TrLR reporter cells transfected with cGAMP (vs. vehicle) were subjected to site-specific I-SceI endonuclease activity in the presence or absence of donor GFP template before being analyzed by flow cytometry for the simultaneous determination of frequencies of NHEJ (reported by mCherry expression as a result of an indel-causing frameshift mutation) and HDR (reported by GFP expression arising from the repair of the GFP expression cassette with an exogenous donor) frequencies (Fig. 7e and Supplementary Fig. 8c). Remarkably, we found that cGAMP, but not the signaling incompetent Lin-cGAMP treatment, significantly reduced the prevalence of HDR. NHEJ frequency, by contrast, was unaffected in either case (Fig. 7f, g and Supplementary Fig. 8d). In accordance with these findings, a comet assay revealed that cGAMP exacerbates CPT-induced DNA damage (Supplementary Fig. 8e, f). Since HEK293-TrLR reporter cells do not express cGAS (Supplementary Fig. 8g), our findings also underscore that cGAS protein is itself not required for cGAMP to effectuate its HDR-suppressive activity. These findings also highlight that cGAMP’s HDR-suppressive activity is distinct from the previously reported HDR-inhibitory activity of cGAS, which is reported to be independent of cGAS’s catalytic activity^12,13^.

We next sought to determine whether cGAMP’s HDR-suppressive activity could be mimicked by cGAS. We observed that HEK293-TrLR cells that inherently lack cGAS expression, when reconstituted with cGAS (cGAS-HEK293-TrLR), activate STING and H2AX following exposure to HT-DNA (Supplementary Fig. 8h). Just as with cGAMP-treated cells, cGAS-reconstituted HEK293 with an integrated TrLR reporter (cGAS-HEK293-TrLR) showed reduced HDR compared to control cells (Ctr-HEK293-TrLR) (Supplementary Fig. 8i, j). Taken together, our studies demonstrate that cGAS-cGAMP signaling suppresses DSB repair via the HDR pathway.

We further examined the effect of cGAMP on NHEJ by monitoring RIF1 and phospho-53BP1 foci^46^. Interestingly, cGAMP stimulation augmented the RIF1 and phospho-53BP1 foci formation induced by etoposide or by CPT (Supplementary Fig. 9a–d). The augmented RIF1 and phospho-53BP1 foci formation possibly reflects the increased DNA damage observed owing to suppression of HDR as revealed by comet assay^12,13^ (Supplementary Fig. 8e).

### cGAS-cGAMP suppresses CRISPR-Cas9-mediated genome editing

**Fig. 8:**
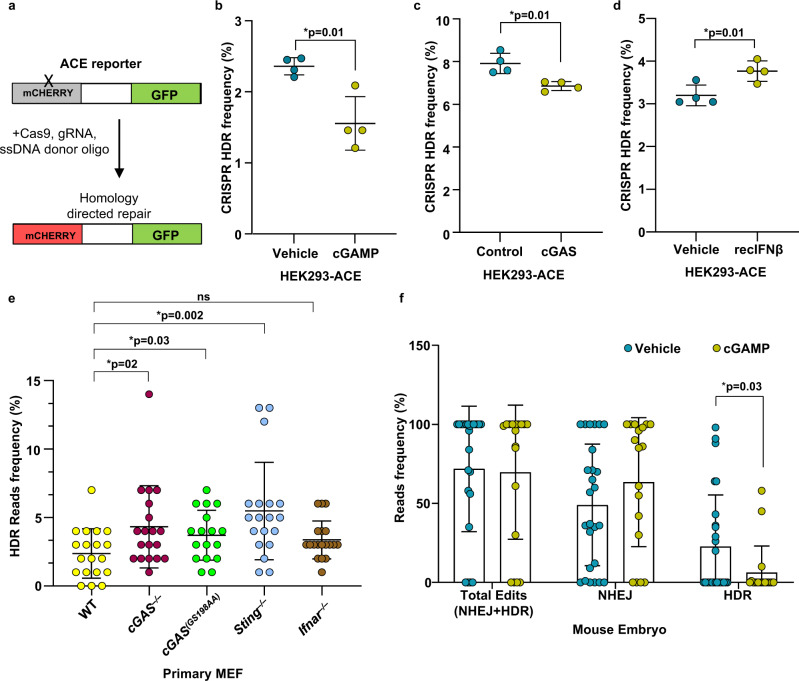
cGAMP suppresses CRISPR-Cas9 genome editing. **a** GFP-positive HEK293-ACE CRISPR/Cas9 reporter cells were transfected with recombinant Cas9, gRNA, and donor template to repair DNA sequences encoding mutant non-fluorescent mCherry to functional fluorescent mCherry expression cassettes. **b** The percentages of HEK293-ACE CRISPR/Cas9 reporter cells mock stimulated and stimulated with cGAMP determined to be mCherry positive are presented (*n* = cell culture replicates, data presented are mean ± s.d.; two-tailed unpaired *t* test; *p < 0.05 indicates significance compared to respective groups; ns indicates not significant). **c** The percentages of HEK293-ACE cells stably expressing cGAS and control (empty plasmid) determined by flow cytometry to be mCherry positive are presented (data presented are mean ± s.d., *n* = 4 cell culture replicates, data presented are mean ± s.d.; two-tailed unpaired *t* test; *p < 0.05 indicates significance compared to respective groups; ns indicates not significant). **d** GFP-positive HEK293-ACE CRISPR/Cas9 reporter cells were treated with human recombinant interferon-β (50 ng/ml) and then transfected with recombinant Cas9, gRNA, and donor template to repair DNA sequences encoding mutant non-fluorescent mCherry to functional fluorescent mCherry expression cassettes, followed by flow cytometry. Quantification of flow cytometry data is presented (*n* = 5 cell culture replicate; data presented are mean ± s.d.; two-tailed unpaired *t* test; *p < 0.05 indicates significance compared to respective groups; ns indicates not significant). **e** The Rosa26 locus was edited using CRISPR/Cas9 in WT, *cGAS*^−/−^, *cGAS*^*(GS198AA)*^, *Sting*^−/−^, and *Ifnar*^−/−^ mouse primary embryonic fibroblasts and subsequently PCR amplified for next-generation sequencing and CRISPResso analysis. The frequency of CRISPR/Cas9-mediated homology-directed repair outcomes in these genotypes are presented (*N* = 19 for WT, *N* = 19 for *cGAS*^−/−^, *N* = 17 for *cGAS*^*(GS198AA)*^, *N* = 19 for *Sting*^−/−^, and *N* = 19 for *Ifnar*^−/−^ cell culture replicates; data presented are mean ± s.d.; two-tailed unpaired *t* test, no adjustments were made for multiple comparisons; *p < 0.05 indicates significance compared to respective groups; ns indicates not significant). Each data point represents percentage of sequence reads, indicative of HDR, *n* = 19 cell culture replicates for each genotype. **f** The frequency of CRISPR/Cas9-mediated genome-editing outcomes in mouse embryos in the presence or absence of cGAMP was determined as described in the schematic (Supplementary Fig. 10c; *n* = 26 embryos for vehicle, *n* = 18 embryos for cGAMP; data presented are mean ± s.d.; two-tailed unpaired *t* test; *p < 0.05 indicates significance compared to respective groups; ns indicates not significant). Each data point represents percentage of sequence reads within the indicated outcomes (Total edits, NHEJ, HDR).

Precise genome editing by the CRISPR/Cas9 system relies on efficient HDR to incorporate desired sequence modifications at a specified genomic locus where guide RNA-targeted Cas9 introduces a DSB^47^. Our observation that cGAS and its catalytic product cGAMP can dampen HDR in human cells prompted us to evaluate CRISPR-HDR frequency in the presence of cGAS/cGAMP using HEK293 cells stably expressing ACE reporter with a mutant mCherry, which is corrected to functional mCherry^48^ following CRISPR/Cas9 editing (Fig. 8a). Consistent with our TrLR reporter data (Fig. 7e–g), either cGAMP transfection or cGAS reconstitution of HEK293-ACE reporter cells significantly dampened HDR-mediated CRISPR editing while the addition of recombinant human IFN had no effect (Fig. 8b–d and Supplementary Fig. 10a, b). Next, to evaluate how cGAS, STING, and IFNAR deficiency may impact CRISPR/Cas9-mediated genome editing, we induced DSBs by CRISPR/Cas9 at the Rosa26 loci in WT, *cGAS*^*−/−*^, *cGAS*^*(GS198AA)*^, *Sting*^*−/−*^, and *Ifnar*^*−/−*^ primary MEFs and analyzed the locus-specific editing outcomes by next-generation sequencing (NGS) of the target locus PCR amplicons. We found that, while the frequency of CRISPR/Cas9-mediated HDR repair outcomes in the *cGAS*^*−/−*^ and *Sting*^*−/−*^ cells increased significantly, the prevalence of HDR in *Ifnar*^*−/−*^ cells was consistent with that in WT cells (Fig. 8e). Furthermore, CRISPR/Cas9-mediated HDR was observed at higher frequency in *cGAS*^*(GS198AA)*^ MEF compared to WT cells (Fig. 8e), therein supporting the conclusion that catalytic activity of cGAS suppresses HDR.

Subsequently, we targeted the Rosa26 loci of one-cell fertilized C57BL/6J embryos for modification by microinjection of single guide RNA (sgRNA), Cas9 protein, and DNA donor templates along with cGAMP or vehicle control (Supplementary Fig. 10c). The resultant genome-editing profiles of embryos were elucidated by NGS of the target locus PCR amplicons (Supplementary Fig. 10c). As was indicated in our cell culture studies (Figs. 7g and 8a–e), CRISPR/Cas9-mediated HDR editing in mouse embryos was significantly diminished in the presence of cGAMP while total editing frequency and NHEJ were unaffected (Fig. 8f). Collectively, these results demonstrate that cGAS-cGAMP signaling suppresses precise HDR CRISPR/Cas9 genome editing in an IFN-independent manner.

### HDR-suppressive activity of cGAMP proceeds independently of its effect on cell cycle

**Fig. 9:**
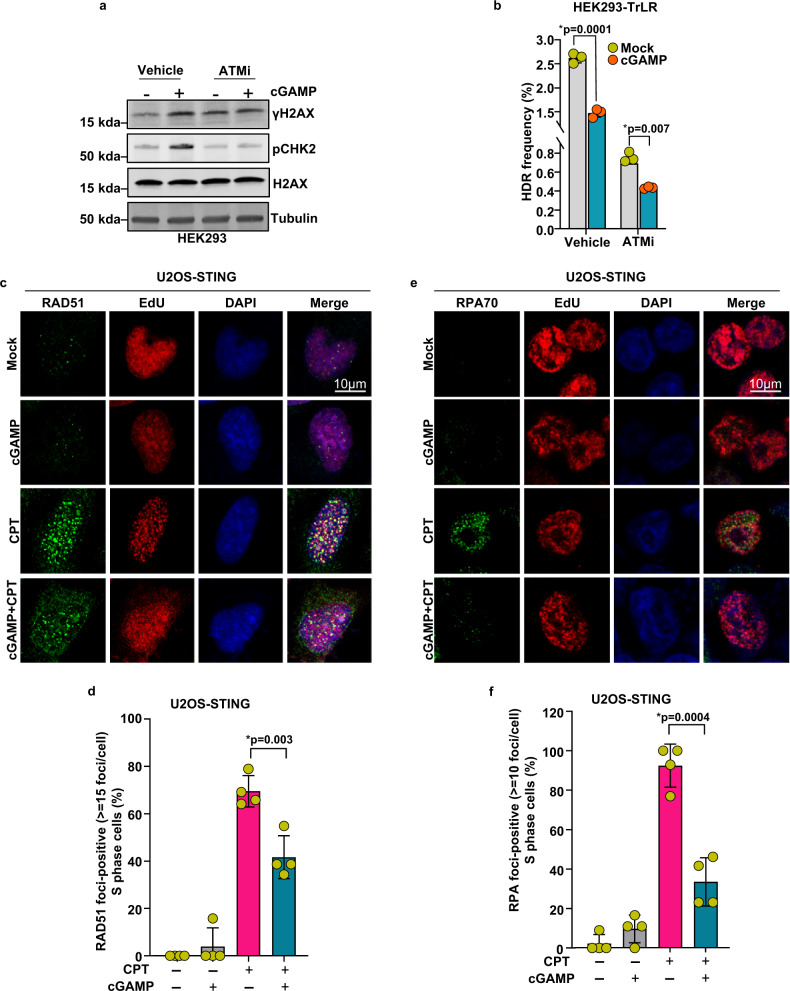
HDR-suppressive activity of cGAMP proceeds independently of its effect on cell cycle. **a** Immunoblots showing phosphorylated H2AX (γH2AX) and CHK2 (pCHK2) in HEK293 cells that were pretreated with 25 μM ATM inhibitor (KU-55933) for 1 h and then transfected with cGAMP for 16 h. Total H2AX (H2AX) and tubulin serve as internal controls. Bands of interest from representative immunoblots from three independent experiments are shown. **b** Quantification of flow cytometric analysis of HEK293-TrLR cells pretreated with 25 μM ATM inhibitor (KU-55933) and then transfected with vehicle/cGAMP before being subjected to the expression of I-SceI with donor for 72 h. HDR events (GFP^+^ cells) are represented as HDR frequency percentages (*n* = 3 independent experiments, data presented are mean ± s.d.; two-tailed unpaired *t* test; *p < 0.05 indicates significance compared to respective groups; ns indicates not significant). **c** Immunofluorescence of RAD51 in EdU^+^ U2OS-STING cells that were transfected with cGAMP for 6 h, then treated with camptothecin (5 μM for 16 h), scale bar = 10 μm. **d** Quantification of RAD51 foci in S phase cells, each data point represents the percentage of EdU^+^ cells with >15 foci in a microscopic field (*n* = 4 fields with over 100 cells collectively per condition, data presented are mean ± s.d.; two-tailed unpaired *t* test; *p < 0.05 indicates significance compared to respective groups; ns indicates not significant). **e** Immunofluorescence of RPA70 in EdU^+^ U2OS-STING cells that were transfected with cGAMP for 6 h, then treated with camptothecin (5 μM for 16 h), scale bar = 10 μm. **f** Immunofluorescence and quantification, respectively, of RPA70 foci in EdU^+^ U2OS-STING cells that were transfected with cGAMP for 6 h followed by camptothecin treatment for (5 μM 16 h), scale bar = 10 μm (*n* = 4 fields with over 100 cells collectively per condition; data presented are mean ± s.d.; two-tailed unpaired *t* test *p < 0.05 indicates significance compared to respective groups; ns indicates not significant).

DNA repair by the HDR pathway is most favored during S and G2 phases^49–52^. Since cGAMP induces G1 arrest (Fig. 7a–c), we examined whether HDR suppression by cGAMP arises from its ability to reduce the prevalence of cells in the G2 and S phases. As ATM is an important activator of the G1/S checkpoint, we tested whether ATM inhibition overcomes cGAMP-induced G1 arrest and, consequently, HDR suppression. Although the ATM inhibitor (KU55933) suppressed the ability of cGAMP to induce γH2AX and pCHK2 (Fig. 9a) and to induce G1 arrest (Supplementary Fig. 7c), cGAMP still suppressed HDR (Fig. 9b, mock vs. cGAMP in ATM inhibitor-treated cells). As reported earlier^53–56^, although ATM inhibition impeded HDR in its own right, cGAMP stimulation of ATM-inhibited cells induced an even greater reduction in HDR activity compared to ATM-inhibited cells in the absence of cGAMP (Fig. 9b, mock vs. cGAMP in ATM inhibitor-treated cells).

One of the important decisive processes in committing cells to HDR is the controlled processing of DSB ends into long stretches of 3’ single-stranded DNA (ssDNA) through a process termed end resection^49,57–59^. The ssDNA strands generated by end resection quickly become coated with the ssDNA-binding protein, replication protein A (RPA), which plays a crucial role in promoting HDR by protecting ssDNA intermediates. RPA is subsequently replaced by recombinase RAD51, which aids in homolog search and the pairing of ssDNA with the complementary strand of the donor DNA^49,57–59^. Using RAD51 and RPA foci formation as surrogate biomarkers of the HDR process^60–64^, we next monitored the HDR-suppressive activity of cGAMP specifically in EdU-labeled S phase cells. Complementing the findings of HDR frequency analysis in ATM-inhibited cells (Fig. 9b), cGAMP suppressed DNA damage-induced RAD51 and RPA foci formation in EdU-positive S phase cells (Fig. 9c–f). Collectively, these results establish that cGAMP can suppress HDR in cells permissive to HDR and that the observed HDR inhibition stems from mechanisms independent of cGAMP-induced G1 arrest.

### cGAMP-induced suppression of polyADP-ribosylation (PARylation) mediates HDR inhibition

**Fig. 10:**
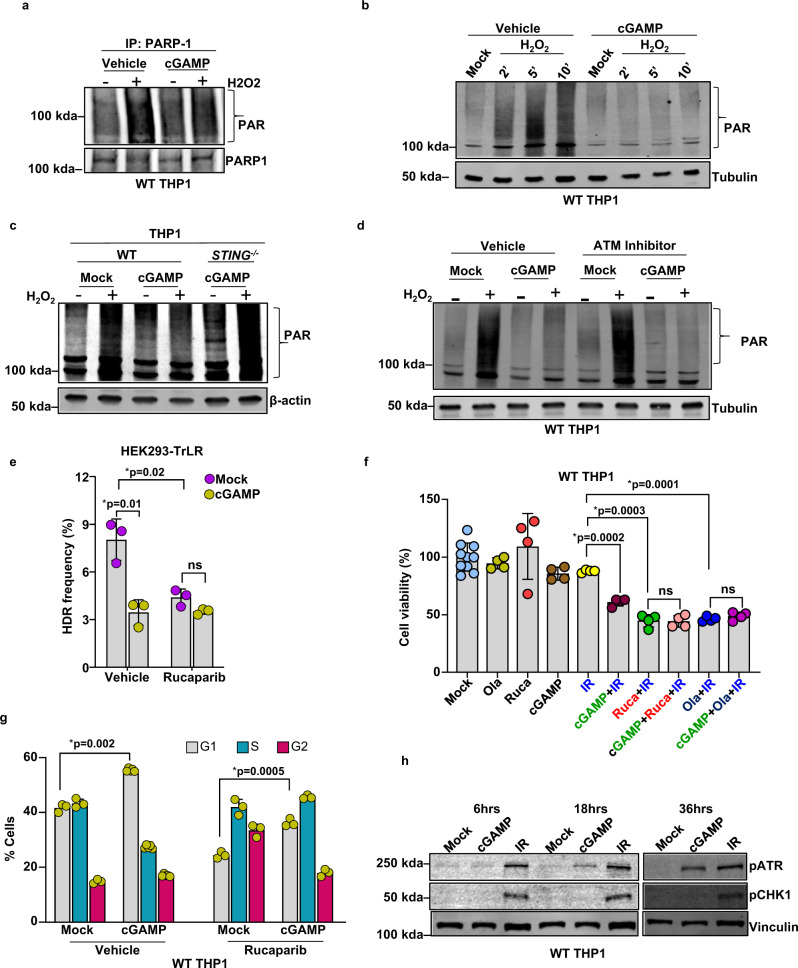
cGAMP-induced suppression of polyADP-ribosylation (PARylation) mediates HDR inhibition. **a** Immunoblots (IB) for polyADP-ribosylated (PAR) proteins of anti-PARP1 immunoprecipitates (IP) from WT THP1 cells treated with vehicle or cGAMP for 6 h and then challenged with 250 μM H_2_O_2_ for 10 min. Bands of interest from representative immunoblots from three independent experiments are shown. **b** Immunoblot of polyADP-ribosylated proteins (PAR) in WT THP1 cells treated with vehicle or cGAMP followed by 250 μM H_2_O_2_ for the indicated time periods. Tubulin served as the loading control. Bands of interest from representative immunoblots from three independent experiments are shown. **c** Immunoblots of polyADP-ribosylated (PAR) proteins in WT and *STING*^*−/−*^ THP1 cells treated with vehicle or cGAMP followed by 250 μM H_2_O_2_ for 10 min. β-Actin served as the loading control. **d** Immunoblots of polyADP-ribosylated (PAR) proteins in WT THP1 cells pretreated with 25 μM ATM inhibitor (KU-55933) or vehicle and then transfected with mock/cGAMP (6 h) and treated with 250 μM H_2_O_2_ (+) for 10 min. Tubulin served as the loading control. Bands of interest from representative immunoblots from three independent experiments are shown. **e** Quantification of flow cytometric analysis of HEK293-TrLR cells pretreated with 10 μM PARP inhibitor (rucaparib) and then transfected with mock/cGAMP (for 6 h) before being subjected to the expression of I-SceI with donor (for 72 h). HDR events (GFP^+^ cells) are represented as HDR frequency percentages (*n* = 3 independent experiments, data presented are mean ± s.d.; *two-tailed unpaired *t* test; *p < 0.016 indicates significance compared to respective groups; ns indicates not significant; adjustments are made for multiple comparisons). **f** Cellular proliferation was assessed using a CellTiter 96 AQueous One Solution Cell Proliferation Assay of WT THP1 cells. Cells were consecutively pretreated with 10 μM of the PARP inhibitor olaparib or rucaparib (1 h), stimulated with cGAMP (6 h), then exposed to 10 Gy ionizing radiation for 48 h before being tested with the viability assay (*n* = 10 for mock or 4 for the rest of the groups, samples are from independent biological replicates, data presented are mean ± s.d.; two-tailed unpaired *t* test; *p < 0.05 indicates significance compared to respective groups; ns indicates not significant). **g** Cell cycle analysis (propidium iodide stain) of WT THP1 cells pretreated with 10 μM of the PARP inhibitor rucaparib (or vehicle) for 1 h and stimulated with mock or cGAMP for 24 h (*n* = 4 independent experiments; data presented are mean ± s.d.; two-tailed unpaired *t* test; *p < 0.05 indicates significance compared to the respective groups; ns indicates not significant). **h** Immunoblots for phosphorylated ATR (pATR) and CHK1 (pCHK1) in WT-THP1 cells post cGAMP treatment (at the indicated time points) or ionizing radiation (1 h). Tubulin serves as the internal control. Bands of interest from representative immunoblots from three independent experiments are shown.

Protein PARylation is a rapid and widespread posttranslational modification that occurs at DNA lesions and is catalyzed by a family of enzymes known as polyADP-ribose polymerases (PARPs). PARylation is foundational to DNA repair in mediating the recruitment of important DNA repair proteins, including HDR factors^65–68^. PARP1 is a dominant member of the PARP family, which, upon recruitment to DNA lesions, undergoes self-PARylation to facilitate the recruitment of HDR factors such as MRE11 and RAD51 to DSBs^65–68^. We thus tested whether cGAMP stimulation affects PARylation and whether such an effect would be responsible for cGAMP’s HDR-inhibitory activity. To examine whether cGAMP affects PARylation, immunoprecipitated PARP1 was analyzed by immunoblotting with anti-poly(ADP-ribose) polymer (PAR) antibodies following treatment of THP1 cells with H_2_O_2_. As expected, H_2_O_2_-induced DNA damage triggered PARP1 PARylation, but this was notably reduced in cGAMP-stimulated cells (Fig. 10a). Additionally, cGAMP-stimulated cells exhibited a substantially reduced total cellular level of protein PARylation when exposed to H_2_O_2_ or to dox (Fig. 10b and Supplementary Fig. 11a). As with cGAMP, cGAS activation by cytosolic DNA (transfected HT-DNA) resulted in reduced PARylation induction in response to H_2_O_2_ (Supplementary Fig. 11b). Whereas STING was required for cGAMP-induced suppression of protein PARylation (Fig. 10c), recombinant IFN-β (recIFN-β), a primary cytokine induced by cGAMP^1^, did not inhibit protein PARylation (Supplementary Fig. 11c), suggesting that cGAMP’s inhibitory effect on protein PARylation is mediated via STING and that canonical IFN signaling alone is not sufficient to trigger cGAMP-driven PARylation inhibition. Additionally, cGAMP-induced suppression of protein PARylation proceeded normally in ATM-inhibited cells (Fig. 10d), suggesting that cGAMP-induced ATM activation and PARylation inhibition are not interdependent. If PARylation inhibition mediated the HDR-suppressive activity of cGAMP, we expected that PARP inhibitor (rucaparib)-pretreated cells with suppressed PARylation would be refractory to cGAMP’s effect on HDR. In supporting this hypothesis, we found that cGAMP was ineffective at HDR suppression in cells pretreated with rucaparib (Fig. 10e). Additionally, the PARP inhibitor rucaparib significantly reduced HDR in TrLR reporter cells (Fig. 10e) and suppressed DNA damage-induced RAD51 and RPA foci formation in EdU-positive S phase cells (Supplementary Fig. 11d–g). Additionally, we found that PARP inhibition by rucaparib treatment, consistent with previous reports^69^, causes increased abundance of S/G2 phase cells (Fig. 10g and Supplementary Fig. 11h). Collectively, these studies show that, despite the increased S phase cells, rucaparib-treated cells showed reduced HDR efficiency, further confirming that the HDR suppression in PARP-inhibited cells in our study was not due to changes in cell cycle.

We next assessed whether cGAMP-induced HDR inhibition confers sensitivity to DNA damage. Cell survival analysis revealed that cells exposed to cGAMP were more sensitive to IR (Fig. 10f, IR vs. IR + cGAMP). However, in agreement with the TrLR reporter assay (Fig. 10e), cGAMP stimulation of rucaparib-pretreated cells was unable to further sensitize cells to IR (Fig. 10f). Collectively, our findings demonstrating (1) cGAMP-driven suppression of protein PARylation, (2) loss of cGAMP’s HDR suppressive activity in cells pretreated with PARP inhibitors, and (3) loss of cGAMP’s ability to confer IR sensitivity to cells pretreated with PARP inhibitors suggest that HDR-suppressive activity of cGAMP is mediated via PARylation inhibition.

Small-molecule PARP inhibitors have been reported to induce replication stress and consequent accumulation of cells in S/G2 phases by activating their ATR and CHK1 proteins^69,70^. In contrast to these findings, cGAMP stimulation promotes G1 arrest in both unperturbed as well as PARP-inhibited cells (Fig. 10g), despite inhibiting PARylation (Fig. 10a–d) and activating ATR (Fig. 10h). As such, despite cGAMP inducing ATR phosphorylation, neither activation of CHK1 nor the expected G2/M arrest was observed (Fig. 7a, d, Supplementary Fig. 4b–e, and Fig. 10h). These findings collectively suggest that cGAMP-induced G1 arrest, which is driven by ATM and its effector CHK2, overwhelms the ATR/CHK1 pathway. Future studies will unravel the detailed mechanism and consequence of cGAMP-induced PARylation inhibition during physiological and cellular dyshomeostatic conditions.

### cGAMP-induced suppression of PARylation is driven by the decline in the NAD^+^ levels

PARylation, the process of synthesis and deposition of polymers of ADP-ribose onto acceptor proteins, is catalyzed by PARPs and requires NAD^+^ as a substrate to generate ADP-ribose monomers for polymerization^71,72^ (Supplementary Fig. 12a). To investigate the mechanism involved in the cGAMP-induced inhibition of protein PARylation, we measured PARP enzymatic activity in the cellular extract of control- and cGAMP-treated THP1 cells. These analyses revealed that cGAMP-induced inhibition of protein PARylation (Fig. 10a–d) was accompanied by a reduction in the PARP enzymatic activity in the lysates of cGAMP-stimulated cells (Supplementary Fig. 12b). Similarly, in a control experiment, extracts from rucaparib-treated cells also showed lower PARP enzymatic activity (Supplementary Fig. 12b). The cGAMP-induced reduction in the PARP enzymatic activity was dependent on STING (Supplementary Fig. 12c). There are 17 members in the PARP family of enzymes, of which, three enzymes—PARP1, PARP2, Tankyrase—synthesize polymers of ADP-ribose on the acceptor proteins, while the remaining catalyze a mono ADP-ribose posttranslational modification^71,72^. We wondered whether the decreased PARP enzymatic activity in cGAMP-stimulated cells can be attributed to a reduction in the levels of these three PARP enzymes with PARylation ability. cGAMP-stimulated cells showed no decrease in the abundance of PARP1, PARP2, or Tankyrase. In fact, the abundance of PARP1 was significantly augmented by cGAMP stimulation (Supplementary Fig. 12d, e). We next tested whether or not cGAMP suppression of protein PARylation resulted from reduced availability of NAD^+^, which provides ADP-ribose monomers for polymerization on to acceptor proteins^71,72^. Consistent with this hypothesis, cGAMP stimulation significantly reduced the abundance of cellular NAD^+^ in a STING-dependent manner (Supplementary Fig. 12f). Similar to cGAMP stimulation, cGAS activation by transfection of HT-DNA also induced decline in the NAD^+^ levels in a cGAS- and STING-dependent manner (Supplementary Fig. 12g, h). Boosting cellular NAD^+^ levels by nicotinamide (NAM) supplementation overcame cGAMP-driven inhibition of cellular PARP enzymatic activity (Supplementary Fig. 12i, j). Importantly, boosting cellular NAD^+^ levels also abrogated cGAMP-driven inhibition of protein PARylation and HDR (Supplementary Fig. 12k, l). Collectively, these findings suggest that cGAMP-induced NAD^+^ decline promotes PARP inhibition and HDR suppression. Future studies should unveil the mechanism involved in the NAD^+^ decline during heightened cGAS activity, which will have significant ramifications for aging and immunity.

### cGAMP activates DNA damage response in *Crassostrea virginica* and *Nematostella vectensis*

An evolutionary analysis of cGAS and STING revealed that they are ancient proteins conserved among a wide variety of species ranging from unicellular choanoflagellates like *Monosiga brevicollis* to complex mammals, including humans^73–75^. Furthermore, STING’s molecular function of binding cyclic dinucleotides (CDNs) is conserved in several invertebrate and vertebrate species^73,74^. The canonical downstream signaling targets of cGAS (namely, IRF3 and type I IFNs) responsible for bestowing antiviral immunity are, however, unique to vertebrates^73,75,76^ (Supplementary Fig. 13a). The establishment of this evolutionary hierarchy prompted us to investigate whether cGAMP can incite DDR in sea anemones *N. vectensis* and eastern oysters *C. virginica*, both of which lack IRF3 and competent type I IFNs, but express STING/TBK1 homologs. Comporting with earlier sequence analysis suggesting that *N. vectensis* and *C. virginica* are capable of binding mammalian cGAMP^74^ (Supplementary Fig. 13b), we observed TBK1 phosphorylation in animals exposed to cGAMP and dox (Supplementary Fig. 13c–h). Further, we observed elevated levels of γH2AX in the animals exposed to cGAMP and dox (Supplementary Fig. 13c–h). The induction of DDR in response to cGAMP in these organisms lacking IFN components suggests that the genome surveillance function of cGAS evolutionarily predates metazoan IFN-based immunity.

## Discussion

Cells endure an almost constant assault of their genomes perpetrated by a host of environmental and endogenous stimuli, including oxidative stress, exposure to chemical mutagens, faulty DNA replication, chromosomal missegregation, and microbial infection. Our study, the first to identify the contribution of a metazoan CDN to genome surveillance mechanisms, reveals an unexpected capacity of cGAMP to activate DDR signaling. The fact that this novel cGAMP signaling mechanism is observed in both vertebrates as well as invertebrates lacking type I IFN suggests that its evolution predates the emergence of IFN-based innate immunity. Additionally, our data showing the suppressive effect of cGAMP signaling on CRISPR-Cas9 mammalian genome editing and homology-directed DSB repair holds far-reaching implications for the understanding of various biological processes and development of more efficient precision genome engineering approaches.

While originally identified as a cytosolic sensor of foreign DNA^1,2^, cGAS-induced IFN signaling has been implicated in an array of other biological contexts, such as autoimmune responses to nuclear^77–79^ and mitochondrial self-DNA^80,81^, cellular senescence^3,10,11^, DNA damage^5,6,10,11^, tumorigenesis^6,8,12^, autophagy^82^, and replicative crisis-induced autophagic cell death^83^. Additionally, a cGAMP-independent role of the cGAS protein has been implicated in the DNA repair processes^12,13^. In unveiling this enzyme as a crucial component of genome surveillance machinery, our present findings further expand the scope of CDN signaling biology in multicellular organisms. Given that DDR pathways are of foundational importance to multiple basic organismal physiologic processes^14^, our research should catalyze investigations into the potential involvement of cGAS in telomerase homeostasis^84^, aging^85,86^, meiotic recombination during gametogenesis^87,88^, and the generation of immune-receptor diversity by V(D)J recombination in developing lymphocytes^89,90^.

The DNA damage response incited by non-canonical cGAMP signaling is characterized by several salient features: (1) ATM and CHK2 activation, Rb hypophosphorylation, and inhibition of E2F target genes leading to G1 cell cycle checkpoint activation, (2) transcriptional modulation of p53 target genes among others, and (3) attenuated HDR of DSBs induced by CRISPR/Cas9 genome editing or I-SceI endonuclease activity. Recent reports propose that p53 upregulation also suppresses CRISRP/Cas9 gene editing in human pluripotent stem cells^91^ and the hTERT-immortalized human retinal pigment epithelial cell line RPE-1^92^. It would therefore be interesting to assess if and how the signaling pathways of p53 and cGAS intersect and interface to produce this shared suppressive effect and whether the contributions of the latter could be targeted to improve the efficacy of genome engineering without perturbing the activities of p53 and related oncogenic pathways.

In response to DNA damage, the kinase activity of ATM is rapidly induced by the phosphorylation of serine at position 1981^29^. Our studies demonstrating the stimulation of ATM autophosphorylation by TBK1 kinase activity unveil a new mechanism of ATM activation and expand the known role of TBK1 to include the coordination of cell cycle checkpoint activation and genome maintenance. In our kinase assay, compared to the reaction with heat-killed TBK1, more ATM auto-phosphorylation was observed in reactions with kinase-dead (KD) TBK1 or TBK1 inhibitor.

It is possible that TBK1 protein, besides its kinase activity, might stabilize the ATM catalytic activity via protein–protein interaction. Future studies are needed to unravel the detailed mechanism of this new mode of ATM activation.

Our findings that cGAS increases in abundance in the nucleus and is found in complex with γH2AX following genomic injury dovetail with studies by Liu et al.^12^, which reported that cGAS is retained in the cytosol by B-lymphoid tyrosine kinase (*BLK*)-maintained constitutive Y215 phosphorylation and that Y215 dephosphorylation promotes nuclear cGAS translocation for recruitment to DNA damage sites^12^ during episodes of genotoxic stress. In this context, cGAS protein molecules recruited to DNA damage sites were observed to interact with γH2AX and PARP1 with cGAS–PARP1 interactions impeding the formation of the PARP1–Timeless complex leading to suppression of DNA repair via HDR^12^. In an independent study, Jiang et al.^13^ reported that cGAS constitutively accumulates in the nucleus and that nuclear cGAS promotes genome destabilization, micronuclei generation, DNA damage-induced cell death, and HDR inhibition, the last of which was attributed to the inhibition of RAD51‐mediated D‐loop formation by DNA-bound cGAS proteins^13^. These two studies collectively reported—albeit by implicating two different mechanisms—that the cGAS protein inhibits HDR without the involvement of its catalytic product cGAMP. In revealing that cGAMP induces DDR signaling, entailing activation of ATM and CHK2, cell cycle checkpoint activation, and suppression of HDR via PARP inhibition, our study not only offers additional mechanisms by which cGAS can impede HDR but more importantly connects the cytosolic DNA immune surveillance function of the cGAS-cGAMP-STING pathway to genome surveillance mechanisms.

The topic of subcellular cGAS localization is rapidly evolving and controversial. We found that, under unperturbed conditions, cGAS both in MEFs and in THP1 cells is predominantly found in the cytosol with low levels of nuclear localization. Furthermore, consistent with the Liu et al.^12^ report, we found that exposure to genotoxic agents triggers nuclear cGAS enrichment and the formation of cGAS-γH2AX complexes. Gentili et al.^31^, Jiang et al.^13^, and Volkman et al.^34^, by contrast, report that cGAS is abundantly present in the nucleus even in unperturbed cells^34^. With respect to the catalytic activity of nuclear cGAS, Gentili et al.^31^ report that the nuclear pool of cGAS can be catalytically activated while multiple other studies have reported mechanisms for cGAS inactivation by nuclear DNA^34,93–98^. These seemingly contradictory findings suggest that the mechanisms governing the subcellular localization of the enzyme and its catalytic activity when present in the nucleus are unclear and warrant further detailed investigation to reconcile these conclusions.

PARylation is an evolutionarily conserved posttranslational modification catalyzed by the PARP family of enzymes that results in covalent polymerization of ADP-ribose units onto amino acid residues of target proteins. PARylation regulates many aspects of human cell biology^71,99,100^. PARylation at DNA lesions promotes DNA repair by facilitating the recruitment of DNA repair factors^65–68^. PARylation is also critical for the recruitment and activity of multiple proteins involved in DNA replication^71,99,101–103^ and plays a crucial role in replication stress by fork reversal and stabilization, which is important for the maintenance of genome stability^68,104^. Our findings that cGAMP stimulation suppresses PARylation expose new potential avenues of research concerning how PARylation-dependent processes interface with antiviral immunity in inducing cytokine programs and interfering with viral DNA synthesis. The PARylation inhibitory activity of cGAS-cGAMP-STING signaling might exacerbate chromosomal instability and hence might contribute to previously reported tumorigenesis^4,12^, tumor cell-autonomous metastasis^8,105^, and IR-induced cell death in rapidly dividing non-cancerous cells such as bone marrow cells in in vivo experiments^13^. Additionally, Liu et al.^12^ reported that cGAS interacted with PARP1 via PAR and that both cGAS–PARP1 interactions and DNA damage-induced nuclear cGAS translocation were blocked by olaparib-mediated inhibition of PARP enzymatic activity^12^. It would thus be interesting to investigate whether cGAMP-induced PARylation inhibition functions in a feed-back loop to restrict uncontrolled nuclear cGAS translocation.

Our studies also report a novel finding that cGAMP-STING signaling reduces the abundance of cellular NAD^+^, a substrate molecule that acts as a source of ADP-ribose needed for PARylation reaction. Analogous to our findings, CDN signaling in bacteria has been recently reported to drive rapid NAD^+^ cleavage. Future investigations should unravel the mechanism by which cGAMP-STING signaling reduces cellular NAD^+^ abundance. It is possible that cGAS-STING signaling impacts homeostatic mechanisms regulating NAD^+^ biosynthesis, degradation, or consumption processes. NAD^+^ is found in all living cells and serves as both a critical coenzyme and a cosubstrate for various metabolic reactions^106,107^. Reduced NAD^+^ has been linked to aging, health span, life span, aging-associated inflammation, and neurodegeneration^106,107^. Therefore, investigating the role of the cGAS-STING pathway in the maintenance of cellular NAD^+^ in health and disease is of broad biological and translational interest.

As the first report on the participation of CDNs in mammalian genome surveillance mechanisms, our study offers new molecular advances into the IFN-independent effector mechanisms of cGAS-cGAMP-STING signaling pathways (Supplementary Fig. 14). Our findings, furthermore, position the innate immune adaptor protein STING and kinase TBK1 as new players in DDR signal transduction. Our data demonstrating the induction of cGAMP-driven DDR in invertebrates, such as *C. virginica* and *N. vectensis*, suggests that the genome surveillance mechanism of cGAS-cGAMP is an important conserved function predating the evolution of type I IFNs in vertebrates. With striking resemblance to our own findings, a similar CDN-directed function has also been observed in the prokaryote *Bacillus subtilis*, wherein DisA, an enzyme with diadenylate cyclase activity, signals the presence of DNA DSBs to cell cycle machinery via its second messenger c-di-AMP^108–110^. This evidence of the early evolutionary origin of CDN-mediated genome surveillance in prokaryotes and invertebrates thus provides a compelling testament to the importance of CDN signaling across all domains of life.

In summary, our study, through its description of the bipartite function of cGAMP, highlights the confluence of two evolutionarily conserved but previously unassociated organismal processes, namely, genome surveillance and innate immunity. Future research will unveil the implications of this unexpected discovery for the broad range of cellular and organismal physiologic and pathologic processes influenced by DDR.

## Methods

### Mice

All animal experiments were approved by the University of Virginia’s Institutional Animal Care and Use Committee. Male and female mice between 10 and 20 weeks of age were used in the study. WT C57BL/6J (Stock No: 000664) and *Stat2*^*−/−*^ (Stock No: 023309) mice were purchased from the Jackson Laboratory. *Ifnar1*^*−/−*^ mice were described earlier^111^ and were a generous gift from M. Aguet. *Irf3*^*−/−*^ mice were a generous gift from T. Taniguchi via M. David^112^. *cGAS*^*−/−*^ mice were generated by K. A. Fitzgerald (University of Massachusetts Medical School) on a C57BL/6 background using cryopreserved embryos obtained from the European Conditional Mouse Mutagenesis Program (EUCOMM)^113^. *Tmem173*^*−/−*^ mice were previously described^114^. Mice were co-housed in barrier animal facility in microisolator cages utilizing individually ventilated cage systems with filtered air and active filter exhaust, 12-h light/12-h dark cycle in temperature- and humidity-controlled environment. All rodent diet (a standard laboratory mouse diet) was irradiated to be sterile, which was provided ad libitum, and sterile water was provided using automatic water systems. Mice were humanely euthanized in a carbon dioxide chamber.

### Generation of catalytically dead cGAS mutant mice (*cGAS*^*GS198AA*^)

Mutant mice with catalytically dead cGAS were developed at the Genome Editing & Animal Models Core of the University of Wisconsin Biotechnology Center by mutating Gly198 and Ser199 to Ala (*cGAS*^*GS198AA*^) via a CRISPR/Cas9 system. Target sites were selected and gRNAs for these target sites were synthesized via in vitro transcription, followed by column clean up and ethanol precipitation purification. One-cell fertilized C57BL/6J embryos were microinjected with a mixture of gRNA (50 ng/µl), ssODN (50 ng/µl), and Cas9 protein (40 ng/µl, PNA Bio). In order to generate *cGAS*^*GS198AA*^, injected embryos were implanted into pseudopregnant recipients, and pups were genotyped at weaning by subcloning and Sanger sequencing of amplified fragments. Founder mice were bred to C57BL/6J mice to generate F1s. gRNA and ssDonor nucleotide sequence is presented in the Supplementary Table 2.

### Cell culture

THP1 cells were cultured in RMPI-1640 media (Thermofisher) supplemented with 10% fetal bovine serum (FBS) and 1% penicillin–streptomycin. Immortalized *cGAS*^*−/−*^ and HA-cGAS-reconstituted *cGAS*^*−/−*^ MEFs^80^ (kind gifts from Gerald S. Shadel) and immortalized human embryonic kidney (HEK293) cells were cultured in Dulbecco’s modified Eagle’s medium (DMEM; Thermofisher) with 10% FBS and 1% penicillin–streptomycin. U2OS cells were purchased from ATCC. All cells were maintained at 37 °C in a 5% CO_2_ environment. Primary MEFs were cultured in Complete DMEM for Primary Cell Isolation (Thermo Fisher) with 10% FBS and antibiotics. Bone-marrow-derived macrophage cells were cultured in DMEM with 10% FBS and 20% L929 supernatants. Immortalized WT and *Tbk1*^*−/−*^ MEF cells were kind gifts from Tom Maniatis’ laboratory and were cultured in DMEM (Thermofisher) with 10% FBS and 1% penicillin–streptomycin.

### Oysters

Wild adult eastern oysters (*C. virginica*) of both male and female sexes ranging from 2 to 7 cm in length (1–3 years in age) were collected from a natural oyster reef on Virgnia’s Eastern Shore. Following collection, the diploid oysters were placed on ice and transported approximately 3 h to the University of Virginia main campus in Charlottesville, VA. There they were stored in an aerated aquarium containing water supplemented with Instant Ocean® Aquarium Sea Salt (50 gm/l). A small hole was made on the oyster shell beneath the adductor muscle where cGAMP (25 µg/50 gm oyster weight) or doxorubicin (1 µg/50 gm oyster weight) was injected. Treated oysters were kept at 4 °C in the dark for 16 h after which they were shucked, had their hemolymph/tissues harvested for analysis by western blotting, and dead oyster and shell were disposed of.

### Starlet sea anemones

WT, laboratory maintained, self-sustaining *N. vectensis* culture containing of 3–6-month-old male and female animals^115^ was a kind gift from Timothy J Jegla (Penn State University Department of Biology). They were maintained in 6-well plates containing water with Instant Ocean® Aquarium Sea Salt (50 gm/l) and fed brine shrimp. To stimulate them with cGAMP, *N. vectensis* were immersed in 0.5 ml digitonin permeabilization solution (50 mM Hepes pH 7.0, 100 mM KCl, 85 mM sucrose, 3 mM MgCl_2_, 0.2% bovine serum albumin (BSA), 1 mM ATP, 0.1 mM dithiothreitol (DTT), 2 μg/ml digitonin) supplemented with 2 μg cGAMP or vehicle for 10 min. Permeabilization solution was removed and the animals returned to maintenance sea salt water. Dox was added to the maintenance sea salt water to a final concentration of 2 µM. Sixteen hours post treatment, *N. vectensis* were collected and washed once with cold phosphate-buffered saline (PBS) before lysing by the addition of 200 μl RIPA buffer. Samples were homogenized by sonication and centrifuged at 4 °C to remove debris. Soluble lysates were quantified for protein analysis by western blot.

### CRISPR/Cas9-mediated gene editing in mouse embryos

Target sites were selected and gRNAs for these target sites were synthesized via in vitro transcription followed by column clean up and ethanol precipitation purification. One-cell fertilized C57BL/6J embryos were microinjected with a mixture of gRNA (50 ng/µl), ssODN (50 ng/µl, IDT, Supplementary Table 2), and Cas9 protein (40 ng/µl, PNA Bio). To determine the effect of cGAMP on genome-editing profiles, 1 fmol/embryo of cGAMP or vehicle was also included. Injected embryos were cultured to blastocysts and then lysed to isolate genomic DNA. The edited locus (Rosa26) was amplified via PCR, and samples were indexed, pooled, and run on a MiSeq Nano 2 × 250 Reagent Kit. Demultiplexed data were analyzed with CRISPResso^116^. Reads ambiguously categorized as “HDR/NHEJ” were manually sorted using a window 30 bp wide flanking the cut site, where reads identical to the expected HDR sequence were recategorized accordingly. Samples with <100 reads were excluded from analysis.

### Cell stimulations

THP1 cells were treated with 500 ng/ml Ultrapure LPS from *Escherichia coli* 0111: B4 (Invivogen, Cat# tlrl-3pelps) or *Salmonella minnesota* (Invivogen, Cat# tlrl-smlps) in standard RPMI complete media. Similarly, THP1 cells in RMPI complete media were stimulated with 500 ng/ml Pam3CSK4 (Invivogen, Cat# tlrl-pms). Primary MEFs were transfected with 0.5 μg of 5′ppp-dsRNA or 4 μg HT-DNA per well of a 6-well plate using with Lipofectamine 2000 as described earlier^117^. Dox (Sigma, Cat# D1515) was supplemented in the THP1 and MEF culture media at 0.5–2 μM. CPT (Cayman Cat# 11694) was used at a concentration of 1–5 μM for the indicated time periods. Cells were exposed to 5 Gy IR and harvested 1 h post-IR. THP1 cells were pretreated with 2 μM TBK1 inhibitor MRT67307 (EMD Millipore, Cat# 506306) or dimethyl sulfoxide vehicle control as described earlier^118^ preceding cGAMP treatment. THP1 cells were stimulated with recombinant human IFN-β (0.5–100 ng/ml) (Peprotech, Cat# 300-02BC) in culture media. Cells were pretreated with 25 μM ATM inhibitor KU-55933 (Sigma Cat# SML1109), 10 μM PARP inhibitor olaparib (Cayman Cat# 10621), or 10 μM PARP1 inhibitor rucaparib (Cayman Cat# 15643) for 2 h. Cells were harvested at the indicated time points for analysis. Cellular NAD^+^ levels were boosted by preincubating cells for 24 h with 2–4 mM NAM (Sigma Cat# 47865-U).

### Lentivirus preparation

Lenti-X™ 293T cell line (Takara, Cat# 632180) was cultured in 10% FBS and 1% penicillin–streptomycin. Cells at 70% confluence were transfected with ViraSafe™ Lentiviral Packaging plasmids (Cell Biolabs Inc. Cat# VPK-206, pCgPv, pRSV-Rev, pCMV-VSV-G) along with transfer vector at a ratio 3:1:1:1 (transfer vector: pCMV-VSV-G: pRSV-REV: pCgPv) using Lipofectamine 2000 transfection reagent (Thermofisher Cat# 11668019). Supernatants containing lentivirus were collected at 24, 48, and 72 h post transfection. Pooled supernatants were centrifuged at 500 × *g* for 5 min to remove cellular debris and filtered through a 0.22-μm Corning® syringe disc-type filter. Lentiviral transduction of target cells was carried out in the presence of 10 μg/ml polybrene.

### CRISPR/Cas9-mediated gene knockout in cells

STING knockout THP1 cells were generated using a human TMEM173 sgRNA CRISPR/Cas9 expressing lentiviral vector (ABMgood Cat# K2402701; Supplementary Table 2). THP1 cells were transduced with lentiviral vectors expressing scramble or sgRNA targeting TMEM173 by incubation with lentiviral supernatant and 10 µg/ml polybrene (Sigma Cat# TR-1003-G) for 24 h. Cells were then allowed to recover for 48 h in complete RMPI media. The lentivirus transduced THP1 cells were selected through exposure to 5 µg/ml puromycin for 2 weeks and cultured to isolate single-cell clones. Knockouts were confirmed by immunoblotting.

### shRNA-mediated knockdown

shRNAs targeting human TBK1, IFNAR1, STAT2, or scramble sequence (Mission shRNA, Sigma-Aldrich Cat# SHC002, Supplementary Table 2) were expressed via lentiviral transduction of THP1 cells as described above. Knockdown of target proteins was assessed by immunoblotting.

### Generation of U2OS-STING cells

WT U2OS cells were reconstituted with STING by integrating LentiORF-TMEM173 (CCSB-Broad Lentiviral Expression Collection, TMEM173, Cat# ccsbBroad304_05465) via lentiviral transduction of U2OS cells as described above. Exposure to blasticidin (10 μg/ml) for 14 days facilitated the selection of STING integrated cells whose protein overexpression profiles were assessed by immunoblotting.

### Primary MEF isolation

Primary MEFs were isolated using the Primary Mouse Embryonic Fibroblast Isolation Kit (Thermofisher, Cat# 88279) according to the manufacturer’s instructions. Mouse embryos were extracted from a euthanized mouse (E11–13) and freshly dissected embryonic tissues were minced into 1–3 mm^3^ fragments in ice-cold Hank’s Balanced Salt Solution (HBSS) buffer. Tissues were washed twice in cold HBSS buffer before 0.2 ml MEF Isolation Enzyme (with Papain) was added to each tube. All samples were then incubated at 37 °C for 30 min. The MEF Isolation Enzyme was removed and the tissues washed twice with cold HBSS buffer. The remaining products were resuspended in 0.5 ml pre-warmed complete DMEM for subsequent primary cell isolation by pipetting up and down. One milliliter of media was added to a single-cell suspension, which was then counted and tested for viability by trypan blue staining and plated according to the manufacturer’s protocol.

### cGAMP delivery and transfection

cGAMP was delivered to THP1 as described previously^44^. In all, 1 × 10^6^ THP1 cells were collected by centrifugation and resuspended in 0.1 ml digitonin permeabilization solution (50 mM Hepes pH 7.0, 100 mM KCl, 85 mM sucrose, 3 mM MgCl_2_, 0.2% BSA, 1 mM ATP, 0.1 mM DTT, 10 μg/ml digitonin) containing 0.5–1 μg cGAMP (Invivogen) or water. Cells were incubated at 37 °C for 20 min after which the permeabilization solution was replaced with RPMI complete media. To transfect cGAMP into primary MEF, HEK293, and U2OS-STING cells, 1 × 10^6^ cells/well were plated on a 6-well plate and transfected with 2–4 μg cGAMP using Lipofectamine 2000 as described previously after 16 h^119^.

### Cell lysis and immunoblotting

Cells were harvested at the indicated time points and lysed in RIPA buffer (Thermofisher Cat# 89900) supplemented with protease inhibitor (Sigma, Cat# 11697498001) and phosphatase inhibitor (Sigma, Cat# 4906845001). Lysates were homogenized by sonication and then centrifuged at 12,000 × *g* for 5 min at 4 °C to remove cellular debris. Pierce BCA Protein Assay Kits (Thermo Fisher Scientific) were used to quantify soluble protein lysates. In all, 20–40 μg of protein were denatured by boiling for 8 min in β-mercaptoethanol and Laemmli buffer. Denatured samples were resolved by sodium dodecyl sulfate-polyacrylamide gel electrophoresis (SDS–PAGE) in Mini-PROTEAN® TGX™ Precast Protein Gels (Bio-Rad) and transferred onto polyvinylidene difluoride membranes using the Trans-Blot Turbo Transfer System (Bio-Rad). Odyssey Blocking Buffer (PBS) or 5% nonfat dry skim milk was used to block membranes for 1 h at room temperature before they were incubated with a primary antibody at 4 °C overnight. Immune-reactive bands were visualized with species-specific secondary antibodies conjugated with IRDye (Licor). Blot images were captured with an Odyssey imaging system. Details of the antibodies used in the western blotting are provided in Supplementary Table 1. Cell Signaling, Thermo Fisher, Novus Biologicals, and Abcam antibodies were used at a 1:1000 dilution, pSTAT2 (Millipore-Sigma Cat# 07-224) at a 1:250 dilution, Santa Cruz Biotechnology antibodies at a 1:200 dilution.

### Immunoblot quantification

Immunoblots were quantified using the Image Studio™ software analysis tool (Licor). Fold change in the bands of interest was calculated after normalizing the signal intensity with loading controls. Uncropped full scan blots are included in the Source data file.

### Cell fractionation

THP1 cells were treated as indicated, then nuclear and cytoplasmic fractions were isolated using NE-PER™ Nuclear and Cytoplasmic Extraction Reagents (Thermo Scientific, Cat# 78833) following the manufacturer’s protocol. Twenty micrograms of cytoplasmic and nuclear proteins were used for immunoblotting.

### Immunoprecipitation

Cells were treated as indicated and were lysed in RIPA buffer. Co-immunoprecipitation experiments were conducted using Dynabeads™ Protein G Immunoprecipitation Kits (Thermo Fisher, Cat# 10007D) as per the manufacturer’s protocol. Fifty microliters of Dynabead slurry was added to a microfuge tube and placed on a magnet to separate the solution. Beads were washed in 200 μl PBS with 0.1% Tween20 (3 times) before 5 μg antibody along with 500–1000 μg protein lysate diluted in PBS-Tween20 to a final volume of 500 μl was added to the beads and incubated overnight at 4 °C with rotation. Beads were collected by magnetic separation and washed 3 times with wash buffer (10 mM Tris; adjust to pH 7.4, 1 mM EDTA; pH 8.0, 150 mM NaCl). Finally, bead-bound proteins were eluted by boiling in 1× Laemmli buffer and samples were analyzed by western blotting.

### Immunofluorescence staining

Sixteen thousand five hundred adherent cells/cm^2^ were plated on a gelatin-coated 8-chamber slide (VWR, Cat# 62407-296) and treated as indicated. Media was removed and cells were washed with 1× PBS with Ca^2+^/Mg^2+^ and fixed with 4% paraformaldehyde (PFA) for 20 min. Fixed cells were permeabilized with PBS + 0.1% Triton-X and washed 3 times with PBS + 0.1% Tween20. After treatment, non-adherent THP1 cells were collected by centrifugation, washed in cold PBS, and fixed in PFA. Post-fixation, they were washed with PBS, spotted on gelatin-coated “PTFE” Printed Slides (Electron Microscopy Sciences, Cat# 63424-06), and air-dried. For γH2AX staining, permeabilized cells were blocked in Serum-free DAKO Protein Block (Agilent, Cat# X0909) for 1 h at room temperature. Cells were incubated in primary antibody (γH2AX, 1:500; anti-HA, 1:100; Supplementary Table 1) in DAKO antibody diluent at 4 °C overnight on a rocker platform. U2OS-STING cells undergoing Rad51 staining were first fixed in methanol for 20 min at −20 °C, blocked for 1 h with donkey block (2% normal donkey serum, 1% BSA, 0.1% Triton X 100, 0.05% Tween20, 0.05% sodium azide in PBS), then finally incubated in an anti-Rad51 antibody overnight at 4 °C (Bio-academia Cat# 70-001, 1:6000 in 5% milk in TBST). For RPA70 staining, cells were fixed in 4% PFA at room temperature for 20 min and blocked in Cell Signaling blocking buffer (1× PBS/5% normal donkey serum/0.3% Triton™ X-100) for 1 h in room temperature and then incubated in an RPA70 antibody (Cell Signaling Cat# 2267, 1:50 in blocking buffer) overnight at 4 °C. Primary antibody was washed 3 times and incubated with secondary antibodies (Thermofisher, Alexa 555, 488) for 1 h at room temperature. Coverslips were mounted on the cells with ProLong™ Gold Antifade mounting media with 4,6-diamidino-2-phenylindole (Thermofisher, Cat# P36935). All images were obtained by confocal microscopy (Nikon, C2).

### Quantitative reverse transcriptase PCR analysis

Cells collected at the indicated time points were washed with ice-cold PBS and lysed in 1 ml Trizol (Invitrogen). Total RNA extracted according to the manufacturer’s recommendations was DNase treated and reverse transcribed to make cDNA with a QuantiTect Reverse Transcription Kit (QIAGEN). The cDNAs synthesized were amplified by real-time quantitative PCR (Applied Biosystems 7900 HT Fast Real-Time PCR system) with Power SYBR green Master Mix. Relative gene expression was determined by the 2−ΔΔCt method, and 18S rRNA or glyceraldehyde 3-phosphate dehydrogenase was used as an internal control. Primer details are provided in Supplementary Table 2.

### Cell cycle analysis by flow cytometry (propidium iodide stain)

In all, 2 × 10^6^ THP1 cells were stimulated with 1 μg cGAMP or vehicle in digitonin permeabilization buffer as described above. Twenty-four hours post cGAMP treatment, THP1 cells were harvested by centrifugation at 300 × *g* for 5 min at 4 °C. Cells were washed with cold PBS and resuspended in 0.5 ml PBS to achieve a single-cell suspension. Cells were fixed in a tube containing 4.5 ml 70% ethanol and kept at 4 °C overnight. Cells were collected by centrifugation at 500 × *g* for 5 min and washed twice with PBS. Washed cells were then stained with 1 ml of freshly prepared staining solution containing propidium iodide (10 μg/ml), DNase-free RNaseA (100 μg/ml), and 0.1% (v/v) Triton X-100 before being subjected to flow cytometry measurement. To determine the percentage of cell population in various cell cycle stages, ~10,000–30,000 cells were acquired on an Attune NxT cytometer and analyzed using the FCS Express software. Cell cycle analysis was performed on propidium iodide-stained cells and single-cell events were determined by gating on the area against the width of the propidium iodide pulse signal. DNA cell cycle modeling and fit was performed using the Multicycle AV plugin (Phoenix Flow Systems).

### HDR and NHEJ assay using Traffic Light Reporter

The effect of cGAMP on the efficiency of DSB repair by HDR and NHEJ was assessed in HEK293 using a Traffic Light Reporter as described earlier^43^. pCVL Traffic Light Reporter 1.1 (Sce target) Ef1a Puro plasmid (TrLR) (Addgene Cat# 31482) was integrated into HEK293 cells by lentiviral transduction as described above. TrLR-integrated HEK293 cells were selected through exposure to 5 µg/ml puromycin for 2 weeks. HEK293-TrLRs were plated on a 6-well plate and allowed to achieve 50% confluency over an 18 h incubation period before being transfected with 1 μg cGAMP using Lipofectamine 2000. Eight hours following this procedure, cells were transfected with pCVL SFFV d14GFP Donor (Addgene Cat# 31475), pCBASceI (Addgene Cat# 26477)), or Donor+SceI using Lipofectamine 3000. Seventy-two hours post-transfection, cells were trypsinized, collected, and analyzed with an Attune NxT flow cytometer (Thermofisher). To quantify the DNA repair events, ~10,000–30,000 cells were acquired on an Attune NxT cytometer and analyzed using the FCS Express software. Single-cell events were determined by gating on the area against the width of the forward scatter pulse signal. Debris removal was performed by excluding events with very low forward and side scatter. For determining the percentage of GFP- and mCherry-positive cells, we established positive and negative populations using mock transfections as negative controls and GFP- or mCherry-transfected samples as Fluorescence Minus One controls.

### ACE CRISPR/Cas9 reporter system

The ACE reporter (Addgene #109428) described previously^48,120^ was integrated in HEK293 cells using lentiviral transduction. ACE reporter integrated cells (GFP+) were sorted in a Becton Dickinson Influx Cell Sorter and propagated. To test the effect of cGAMP on CRISPR/Cas9 editing, HEK293-ACE reporter cells were initially transfected with 0.4 μg cGAMP per well of a 48-well plate and with Cas9 protein along with mCherry+43 gRNA and mCherry ssDNA donor template (Supplementary Table 2) 16 h later using jetCRISPR™ RNP transfection reagent (Cat# 55-151) according to the manufacturer’s protocol (see below for details). Seventy-two hours post RNP transfection, cells were trypsinized, collected, and analyzed by flow cytometer (Thermofisher). To quantify the HDR DNA repair events, ~10,000–30,000 cells were acquired on an Attune NxT cytometer and analyzed using the FCS Express software. Single-cell events were determined by gating on the area against the width of the forward scatter pulse signal. Debris removal was performed by excluding events with very low forward and side scatter. For determining the percentage of mCherry-positive cells, we established positive and negative populations using mock transfections as negative controls and mCherry-transfected samples as Fluorescence Minus One controls.

### CRISPR/Cas9-mediated Rosa26 locus modification in primary MEFs

CRISPR/Cas9-mediated Rosa26 locus modification in primary MEFs was performed using jetCRISPR™ RNP transfection reagents (Cat# 55-151) according to the manufacturer’s protocol in a 96-well plate. The ribonucleoprotein (RNP) complex was prepared by mixing 1.4 µl of 1 µM Cas9 solution with 1.4 µl of 1 µM gRNA (molar ratio 1:1) and adding 9.7 µl OptiMEM. This solution was allowed to incubate for 10 min. Next, 0.1 µg ssDNA was added to the RNP mix and incubated for 5 min. Finally, 0.4 µl JetCRISPR transfection reagent was added to the mix and incubated for 10 min at room temperature. The transfection mix was added to a 96-well plate and 30,000 primary MEF cells were added into each well. Seventy-two hours post transfection, cells were washed with PBS, collected in 75 µl of 5 mM Tris-pH 8.8, and sent for NGS.

### Comet assays

DNA damage in individual cells was assessed using the OxiSelect™ Comet Assay Kits (Cell Biolabs, Cat# STA-351) according to the manufacturer’s protocol. THP1 cells were treated as indicated, collected by centrifugation, washed twice with ice-cold PBS, and resuspended in cold PBS at 1 × 10^5^ cells/ml. Cells were mixed with molten Comet agarose (1:10, v/v) and immediately transferred to wells of the Comet Agarose Base Layer (pre-prepared by adding 75 μl molten agarose to Comet slides) in 75 μl aliquots. Cells were embedded on the agarose by cooling the gel at 4 °C for 15 min in the dark and then lysed in pre-chilled lysis buffer for 60 min at 4 °C. The cell-containing slides were neutralized in pre-chilled alkaline buffer for 30 min and then carefully transferred to a horizontal electrophoresis chamber. Electrophoresis was conducted in cold Alkaline Electrophoresis Solution (300 mM NaOH, pH > 13, 1 mM EDTA) for 30 min at 1 volt/cm (300 mA current). The samples were washed three times in pre-chilled water and immersed in 70% ethanol for 5 min. The cells were subsequently air dried and their DNA was stained with 100 µl/well of diluted Vista Green DNA Dye. After incubating at room temperature, cells were finally visualized under a fluorescence microscope using an FITC filter. Comet length was quantified using the OpenComet software as described before^121^.

### Kinase assay

WT THP1 cells were lysed in lysis buffer (1% (V/V) Nonidet P-40, 50 mM MOPS, pH 7.5, 5 mM MgCl_2_, 0.5 mM MnCl_2_, 100 mM NaCl, 0.4 mM Pefabloc SC, 0.1% (V/V) 2-mercaptoethanol) and endogenous ATM was immunoprecipitated using Abcam Anti-ATM antibody (ab78) in concert with the Dynabeads™ Protein G Immunoprecipitation Kits (Thermo Fisher, Cat# 10007D) as per the manufacturer’s protocol. ATM-bound beads were washed 3 times with kinase buffer (25 mM Mops (pH 7.5) 50 mM NaCl, 10 mM MgCl_2_, 1 mM MnCl_2_, 0.1% 2-mercaptoethanol, 20 mM beta-glycerophosphate) and dephosphorylated with Lambda Protein Phosphatase (NEB Cat# P0753S). Dephosphorylated ATM beads were incubated with recombinant GST-tagged TBK1 (Sigma Cat# SRP5089) in kinase buffer along with 10 μM [γ-^32^P] ATP for 24 h at room temperature. The reaction was stopped by adding 2× SDS sample buffer, and proteins were eluted from the beads by incubating at 98 °C for 10 min. Eluted proteins were separated on a 7.5% SDS/PAGE gel. The gel was exposed to an X-ray film for 24 h at room temperature and developed.

### ATM and GST-TBK1 interaction assay

HEK293-GFP cells were lysed in lysis buffer (1% (V/V) Nonidet P-40, 50 mM MOPS, pH 7.5, 5 mM MgCl_2_, 0.5 mM MnCl_2_, 100 mM NaCl, 0.4 mM Pefabloc SC, 0.1% (V/V) 2-mercaptoethanol). Endogenous ATM and GFP were immunoprecipitated using an Abcam Anti-ATM antibody (ab78) and an anti-GFP antibody (ab6556), respectively, in concert with the Dynabeads™ Protein G Immunoprecipitation Kits (Thermo Fisher, Cat# 10007D) as per the manufacturer’s protocol. ATM/GFP-bound beads were washed 3 times with IP buffer and incubated with recombinant GST-TBK1 (Sigma Cat# SRP5089) in IP buffer at 30 °C for 1 h. Beads were collected using magnetic separator and washed with IP buffer 3 times. Proteins were eluted by incubating at 98 °C and analyzed by western blotting to detect TBK1.

### TBK1 kinase assay using recombinant ATM as substrate

One hundred nanograms of recombinant ATM (Sigma Cat# 14-933) was mixed with 100 ng of recombinant GST-tagged TBK1 (Sigma Cat# SRP5089) in kinase buffer (25 mM Mops (pH 7.5) 50 mM NaCl, 10 mM MgCl_2_, 1 mM MnCl_2_, 0.1% 2-mercaptoethanol, 20 mM beta-glycerophosphate) along with 10 μM ATP for 18 h at room temperature. 2× Laemmli buffer was added and the mixture was then incubated at 98 °C for 10 min. The protein mixtures were separated on a 7.5% SDS/PAGE gel and immunoblotted for anti-phospho-ATM (Ser1981) antibody to assess phosphorylation status of ATM. To test the effect of inhibitors on recombinant TBK1-mediated ATM phosphorylation, 25 µM ATM inhibitor and 10 µM TBK1 inhibitor were pre-incubated with recombinant ATM or recombinant TBK1 (TBK1 inhibitor) before setting up the kinase assay as described above.

### TBK1 kinase assay with catalytically dead ATM

Flag-tagged WT and KD-ATM were (Addgene Cat# 31986) expressed in HEK293 cells by transfecting expression plasmids (Addgene Cat# 31985 and Cat# 31986) for 36 h. FLAG-tagged ATM proteins were immunoprecipitated using Dynabead slurry premixed with anti-FLAG antibody (Cell Signaling 2368). WT ATM beads and KD-ATM beads were washed with cold RIPA buffer (3×) and with cold kinase assay buffer without ATP (1×) and resuspended in the same buffer. WT and KD-ATM beads were incubated with recombinant TBK1 in kinase buffer (described previously in kinase assay) along with 10 μM ATP for 18 h at room temperature. The reaction was stopped by adding 2× SDS sample buffer and proteins were eluted from the beads by incubating at 98 °C for 10 min. Eluted proteins were separated on a 7.5% SDS/PAGE gel and immunoblotted for anti-phospho-ATM (Ser1981) antibody and total TBK1.

### PolyADP-ribosylation

cGAMP was delivered to THP1 cells by digitonin permeabilization as described earlier. Six hours later, the digitonin-permeabilized cGAMP-treated and mock cells were treated with 250 μM H_2_O_2_ for the indicated time periods or 10 min to induce protein polyADP-ribosylation. Cells were collected by centrifugation, washed once with ice-cold PBS, lysed in RIPA buffer, and analyzed by western blot to detect PARylation using pADPr antibody (Santa Cruz sc-56198).

### Cell proliferation assay: BrdU staining

cGAMP or vehicle-treated WT THP1 cells were incubated in 10 μM BrdU (used from the kit) for 1 h prior to harvesting. Cells were fixed and stained to detect BrdU incorporation by flow cytometry following the manufacturer’s protocol (FITC BrdU Flow Kit, BD Biosciences, Cat# 559619).

### Cell proliferation assay: EdU staining

Human RPE cells and U2OS-STING-overexpressing cells transfected with vehicle or cGAMP were incubated with 10 μM EdU (included in the kit) for 2 h prior to harvesting. EdU-incorporated cells were detected by confocal microscopy after Click-iT® EdU labeling following the manufacturer’s protocol (Click-iT™ EdU Alexa Fluor™ 594 Imaging Kit, Thermo Fisher Cat# C10339).

### Cell viability assay

MTS (3-(4,5-dimethylthiazol-2-yl)-5-(3-carboxymethoxyphenyl)-2-(4-sulfophenyl)-2H-tetrazolium) assays were performed using the CellTiter 96 AQueous One Solution Cell Proliferation Assay (Promega) according to the manufacturer’s instructions. THP1 cells (8000 cells/100 μl/well) pretreated with PARP inhibitor rucaparib and olaparib were treated with cGAMP and then irradiated with 10 Gy IR. Forty-eight hours later, MTS assay was performed according the to manufacturer’s protocol.

### cGAMP ELISA

In all, 2 × 10^6^ cells THP1 cells were treated with 1 μM dox for 1 h and subsequently harvested by centrifugation to measure cGAMP concentrations using a 2′3′-cGAMP ELISA Kit (Cayman Cat# 501700) in accordance with the manufacturer’s protocol.

### PARP activity assay

WT THP-1 cells were treated with cGAMP for 6 h using digitonin permeabilization as described earlier. Post cGAMP treatment, cells were collected by centrifugation and washed with cold PBS once and resuspended in Cell Extraction Buffer. PARP activity was measured using the Chemiluminescent Assay Kit (R&D, Catalog Number: 4685-096-K) following the manufacturer’s protocol.

### NAD^+^ quantification

THP-1 cells were treated with cGAMP for 6 h using digitonin permeabilization as described earlier. Post cGAMP treatment, cells were harvested and NAD^+^ was measured using the NAD/NADH Quantification Kit (Catalog Number MAK037) following the manufacturer’s protocol.

### Phylogenetic tree and protein sequence alignment

The phylogenetic tree was generated using the online platform Phylot(v2), a phylogenetic tree generator, based on NCBI or GTD taxonomy. NCBI taxonomy ID was used for *N. vectensis* (45351), *C. virginica* (6565), *Mus musculus* (39442), and *Homo sapiens* (9606). The protein sequence alignment of STING proteins was performed on CLUSTAL multiple sequence alignment platform by MUSCLE (3.8). The input STING amino acid sequences are as follows: *H. sapiens*: NP_938023.1, *M. musculus*: NP_082537.1, *N. vectensis*: XP_001620539.1, *C. virginica*: XP_022323329.1.

### Statistical analysis

Statistical methods are stated in the figure legends. In all cases, a *p* value of ≤0.05 was considered significant (*). GraphPad Prism 7 software and Microsoft Excel were utilized for numerical data tabulation, generation of bar graphs, and ascertainment of statistical significance.

### Reporting summary

Further information on research design is available in the Nature Research Reporting Summary linked to this article.

## Supplementary information

Supplementary Information

Reporting Summary

## Data Availability

Authors can confirm that all relevant data are included in the paper and/or its Supplementary Information files. All genetic material and reagents generated for this paper are available from the authors on request. Mandatory deposition of data in public repository is not required for the data types included in this manuscript. Source data are provided with this paper.

## Source data

Source Data
