# Supplementary material for: A non-canonical, interferon-independent signaling activity of cGAMP triggers DNA damage response signaling
Source: Nat Commun. 2021 Oct 27;12:6207. doi: 10.1038/s41467-021-26240-9 (PMC8551335; doi:10.1038/s41467-021-26240-9)
Supplement: Supplementary file 1 — Supplementary Information [file 41467_2021_26240_MOESM1_ESM.pdf]

Supplementary Information

A non-canonical, interferon-independent signaling activity of cGAMP triggers DNA damage response signaling

Daipayan Banerjee, Kurt Langberg, Salar Abbas, Eric Odermatt, Praveen Yerramothu, Martin Volaric, Matthew A. Reidenbach, Kathy J Krentz, C. Dustin Rubinstein, David L. Brautigan, Tarek Abbas, Bradley D. Gelfand, Jayakrishna Ambati, and Nagaraj Kerur*

*Correspondence and requests for materials should be addressed to:

kerur.1@osu.edu

Supplementary Figure 1

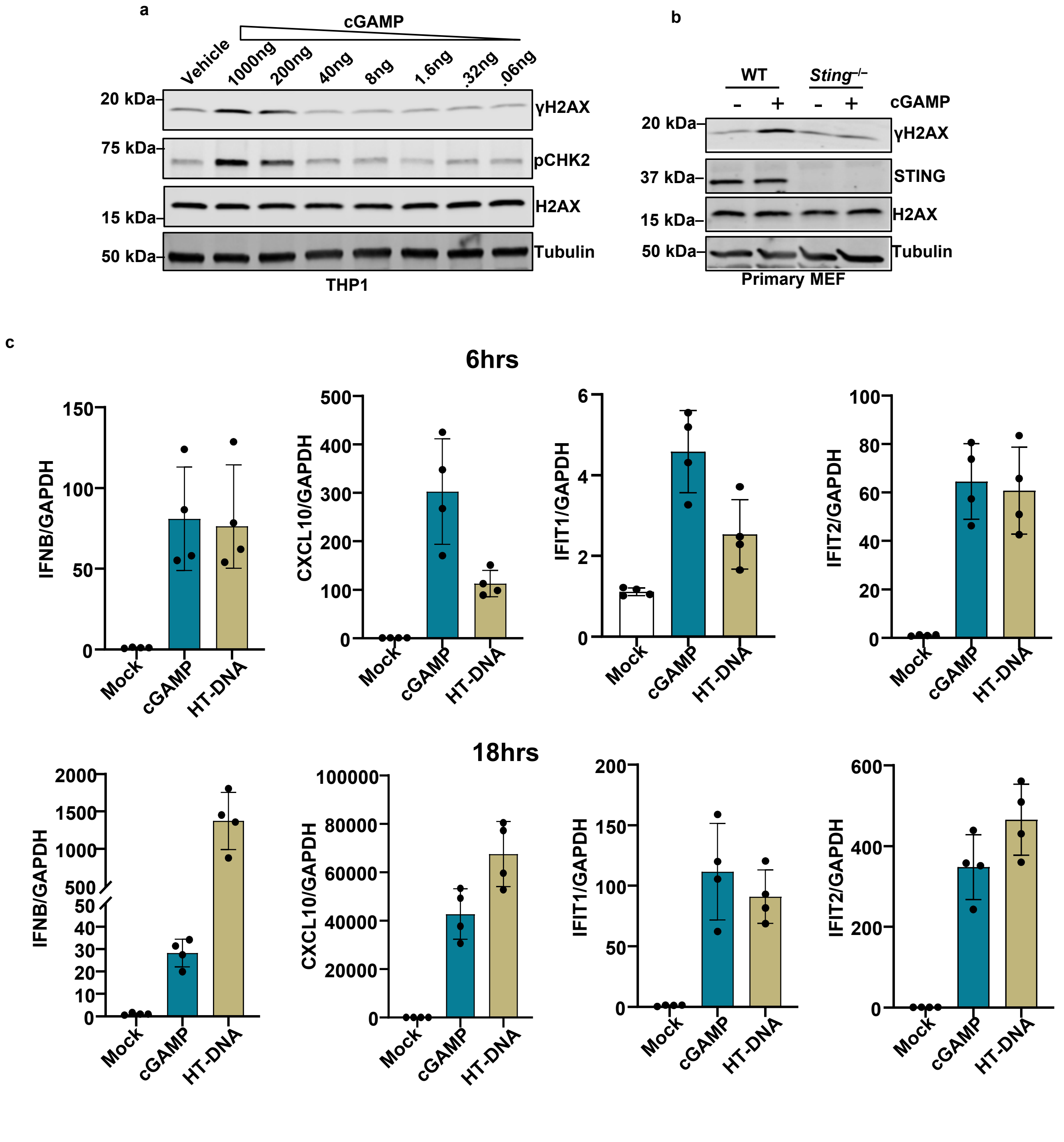

Supplementary Figure 1: A) Immunoblots for phosphorylated H2AX (γH2AX) and CHK2 (pCHK2) in THP1 cells stimulated with vehicle or various doses of cGAMP as indicated for 16hrs. Bands of interest from representative immunoblots from three independent experiments are shown. B) Immunoblots for γH2AX and STING in WT and *Sting*$^{-/-}$ primary MEF mock transfected (-) or transfected with cGAMP (+) for 16hrs. Bands of interest from representative immunoblots from three independent experiments are shown. C) Comparison of levels of IFN-β and ISGs induction in THP1 cells, by stimulation with exogenous cGAMP (1μg) to that of endogenous cGAMP induced by most commonly adopted method of cGAS activation by cytosolic transfected HT-DNA (2μg) (n=3 independent experiments, data presented are mean ± s.d.).

Supplementary Figure 2

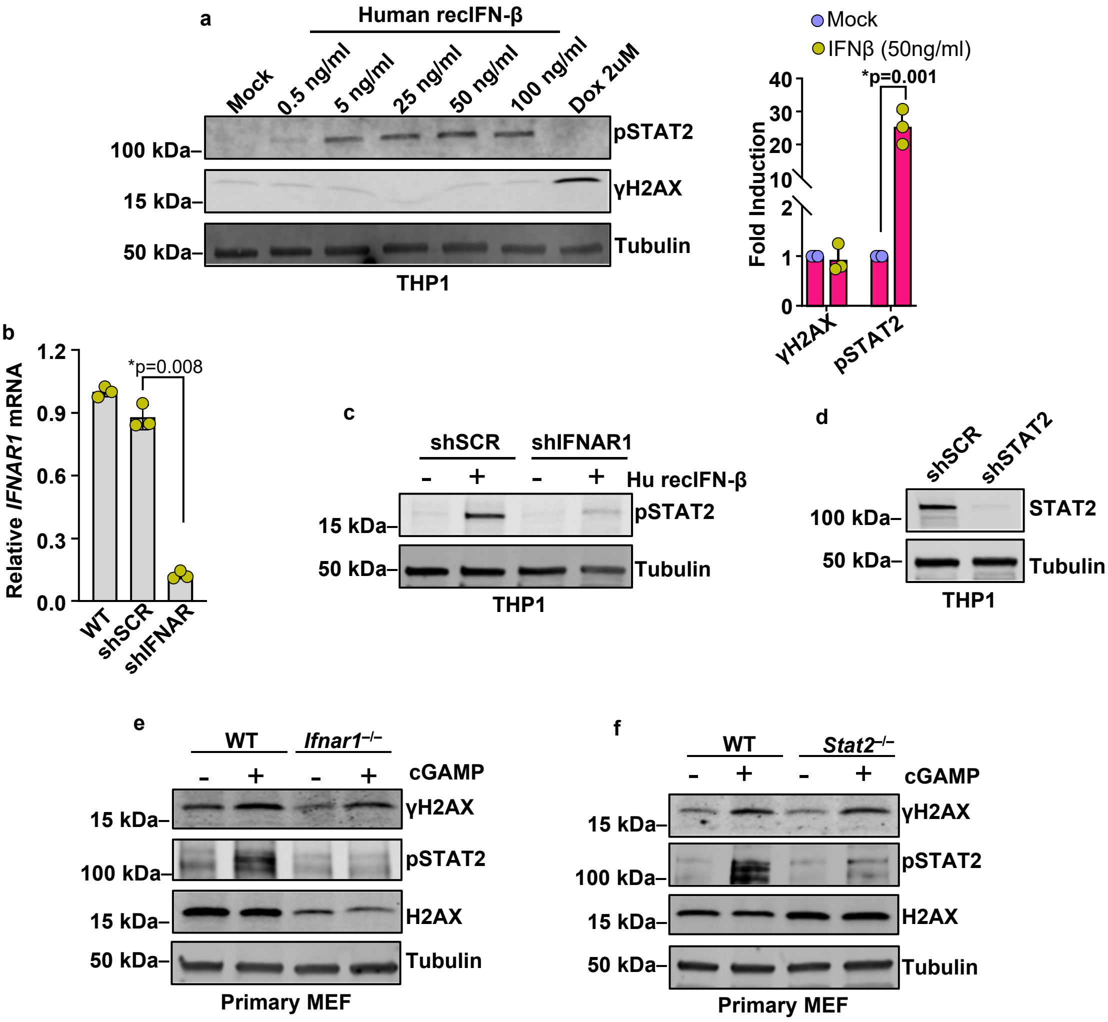

Supplementary Figure 2: A) Immunoblots for phosphorylated STAT2 (pSTAT2) and H2AX (γH2AX) in WT THP1 cells treated with indicated doses of recombinant human interferon β for 6hrs. Doxorubicin (2μM for 16hrs) was used as positive control to induce H2AX phosphorylation. Tubulin was used as a loading control. Quantification of γH2AX and pSTAT2 bands is presented in the bargraph (n= 3 independent experiments; data presented are mean ± s.d.; two-tailed paired *t* test; *p<0.05 indicates significance compared to respective groups; ns indicates not significant). B) Confirmation of *IFNAR1* mRNA knockdown by qPCR in THP1 cells transduced with lentivirus expressing shIFNAR or shScrambled (shSCR) and in untransduced THP1 cells (WT) (n= 3 independent biological replicate; data presented are mean ± s.d.; *$p<0.05$, unpaired *t* test). C) Responsiveness to recombinant human interferon β (Hu recIFN-β) (50ng/ml for 6hrs) was tested in control (shSCR) and shIFNAR1 THP1 cells by immunoblotting for phosphorylated STAT2. Bands of interest from representative immunoblots from three independent experiments are shown. D) Confirmation of target knockdown in shSTAT2 THP1 cells by immunoblotting for total STAT2. Bands of interest from two immunoblotting assessment of knockdown efficiency in the cell line is presented. E) Immunoblots for γH2AX and pSTAT2 in WT and *Ifnar1*$^{-/-}$ primary MEF mock transfected (-) or transfected with cGAMP (+) for 16hrs. Bands of interest from representative immunoblots from three independent experiments are shown. F) Immunoblots for phosphorylated H2AX (γH2AX) and STAT2 (pSTAT2) in WT and *Stat2*$^{-/-}$ primary MEF mock transfected (-) or transfected with cGAMP (+) for 16hrs. Total H2AX, tubulin and/or β-actin were used as loading controls for immunoblots as indicated. Bands of interest from representative immunoblots from three independent experiments are shown.

Supplementary Figure 3

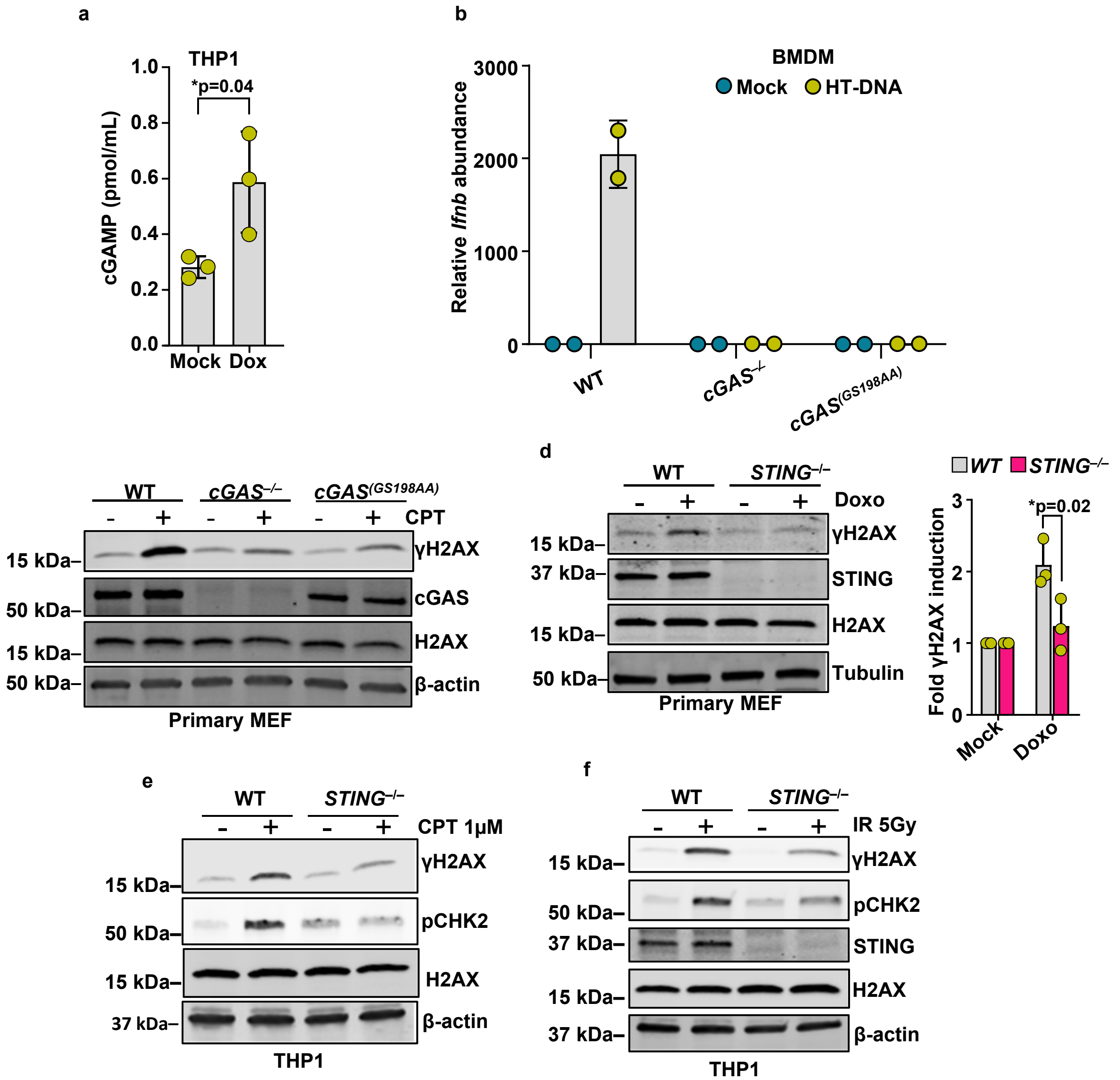

Supplementary Figure 3: A) cGAMP concentrations were measured in WT THP1 cells 1hr post 1μM Doxorubicin treatment using a 2'3'-cGAMP ELISA Kit. n= 3 independent samples; data presented are mean ± s.d.; two-tailed, unpaired *t* test; *p<0.05 indicates significance compared to respective groups; ns indicates not significant B) Analysis of *Ifnb* gene expression by quantitative real-time PCR (RT-qPCR) in WT, *cGAS*$^{-/-}$, and *cGAS*$^{(GS198AA)}$ BMDM transfected with HT-DNA or mock transfected (2μg per 6 well for 4hrs). n= 2 independent cell culture replicates; data presented are mean ± s.d. C) Immunoblots for γH2AX and cGAS in whole cell lysates collected from WT, *cGAS*$^{-/-}$, and catalytically inactive mutant *cGAS*$^{(GS198AA)}$ primary mouse embryonic fibroblast (MEF) cultures treated with camptothecin 1μM for 2hrs (+) or mock treated (-). Bands of interest from representative immunoblots from three independent experiments are shown. D) Immunoblots for γH2AX and STING in whole cell lysates from WT and *Sting*$^{-/-}$ primary MEF's mock treated (-) or treated with doxorubicin (0.5μM for 2hrs) (+). Quantification of γH2AX bands is presented in the bargraph (n= 3 independent experiments; data presented are mean ± s.d.; two-tailed paired *t* test; *p<0.05 indicates significance compared to respective groups; ns indicates not significant). E-F) Immunoblots for γH2AX, pCHK2, and STING in whole cell lysates from WT and *STING*$^{-/-}$ THP1's mock treated (-) or treated with camptothecin (1μM for 2hrs) or ionizing radiation (5Gy for 1hr) (+). Total H2AX, tubulin and/or β-actin were used as loading controls for immunoblots as indicated. Bands of interest from representative immunoblots from three independent experiments are shown.

Supplementary Figure 4

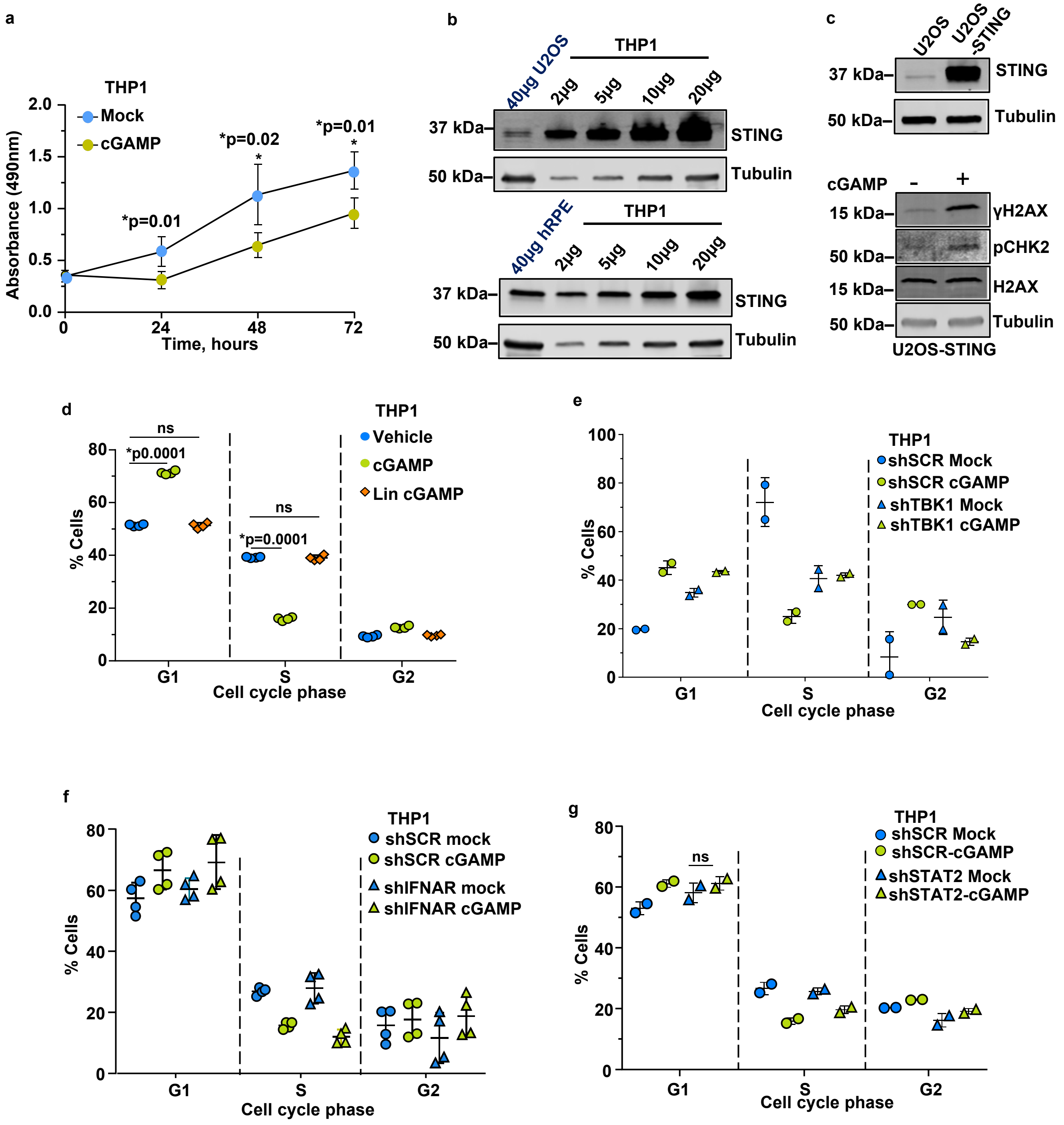

Supplementary Figure 4

Supplementary Figure 4: A) Cellular proliferation in WT THP1 cells post cGAMP treatment at indicated time points was assessed using a CellTiter 96 AQueous One Solution Cell Proliferation Assay (n= 4, cell culture replicates; data presented are mean ± s.d.; two-tailed unpaired *t* test; *p<0.05 indicates significance compared to respective groups; ns indicates not significant). B) STING expression in U2OS (upper panel) and human RPE cells (lower panel) was compared to that in varying indicated amounts of WT THP1 whole cell lysate. Bands of interest from representative immunoblots from two independent experiments are shown. C) Immunoblots for total STING in WT U2OS cells and STING-reconstituted U2OS cells (upper panel) and γH2AX and pCHK2 in STING reconstituted U2OS cells (U2OS-STING) that were transfected with cGAMP for 18hrs (lower panel). Tubulin and total H2AX served as loading controls. Bands of interest from representative immunoblots from three independent experiments are shown. D) WT THP1 cells were stimulated with vehicle, signaling incompetent linear cGAMP (Lin-cGAMP), or cGAMP. 24hrs later cell cycle analysis (propidium iodide stain) was performed by flow cytometry (n= 4, cell culture replicates; data presented are mean ± s.d.; two-tailed unpaired *t* test; *p<0.05 indicates significance compared to respective groups; ns indicates not significant). E) Control (shSCR) and shTBK1 THP1 cells were stimulated with vehicle or cGAMP. After 24hrs cell cycle analysis (propidium iodide stain) was performed by flow cytometry (n= 2 independent experiments; data presented are mean ± s.d.). F) Cell cycle analysis (propidium iodide stain) in control (shSCR) and shIFNAR1 THP1 cells 24hrs after stimulation with cGAMP or vehicle (n= 2 independent experiments; data presented are mean ± s.d.). G) Control (shSCR) and shSTAT2 THP1 cells were stimulated with vehicle or cGAMP. After 24hrs cell cycle analysis (propidium iodide stain) was performed by flow cytometry (n= 2 independent experiments; data presented are mean ± s.d.).

Supplementary Figure 5

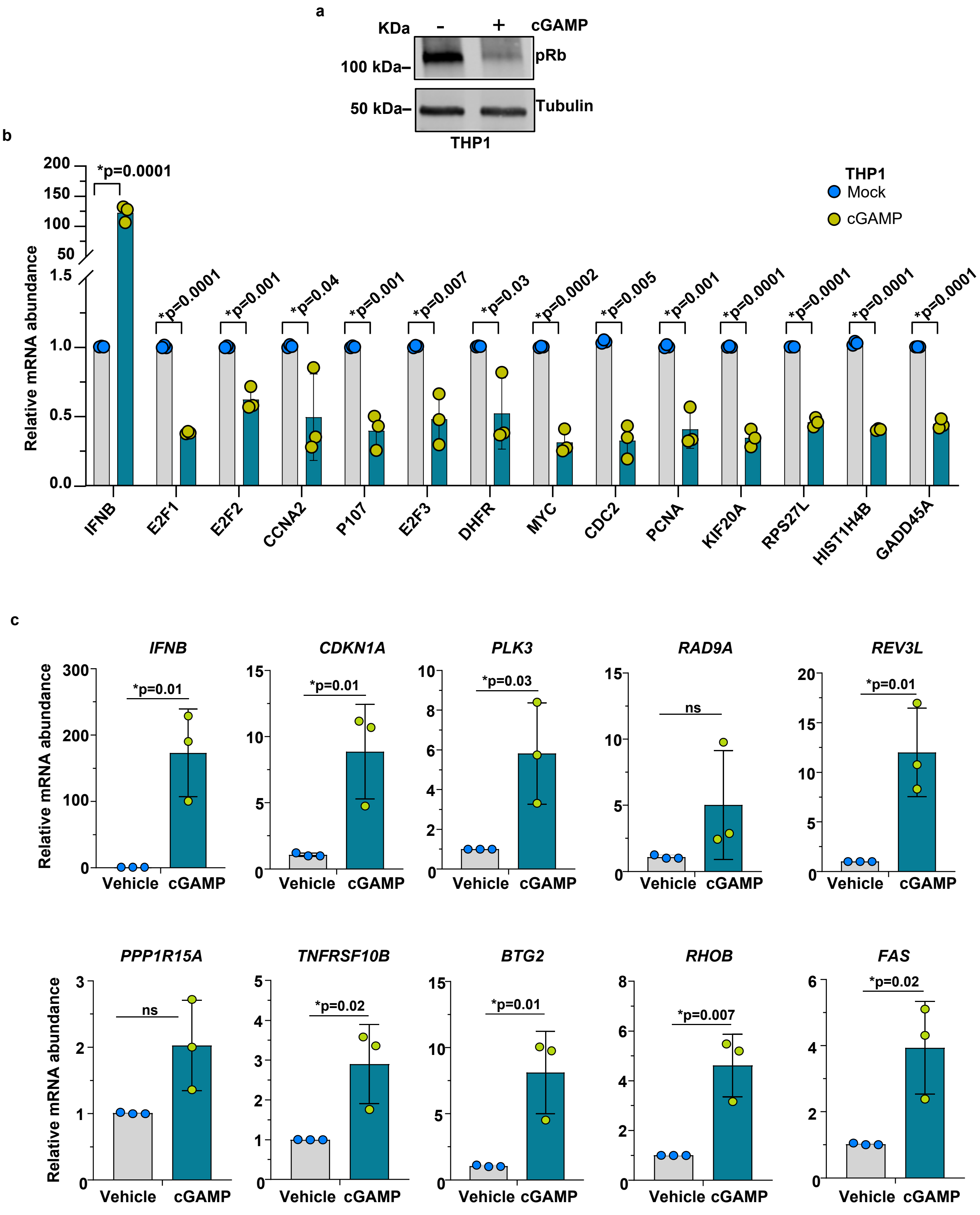

Supplementary Figure 5

Supplementary Figure 5: A) Immunoblot of phosphorylated retinoblastoma protein (pRb) 18hrs post cGAMP treatment in WT THP1 cells. Tubulin serves as the internal control. Bands of interest from representative immunoblots from three independent experiments are shown. B) Gene expression analysis of the E2F gene family in WT THP1 cells stimulated with vehicle or cGAMP for 6hrs by quantitative real-time PCR (RT-qPCR) (n=3 independent experiments, data presented are mean ± s.d.; two-tailed unpaired *t* test; *p<0.05 indicates significance compared to respective groups; ns indicates not significant). C) Analysis of DNA Damage Response (DDR) gene expression for ten DDR genes by quantitative real-time PCR (RT-qPCR) in WT THP1 cells stimulated with vehicle or cGAMP for 6hrs (n=3 independent experiments, data presented are mean ± s.d.; two-tailed unpaired *t* test; *p<0.05 indicates significance compared to respective groups; ns indicates not significant).

Supplementary Figure 6

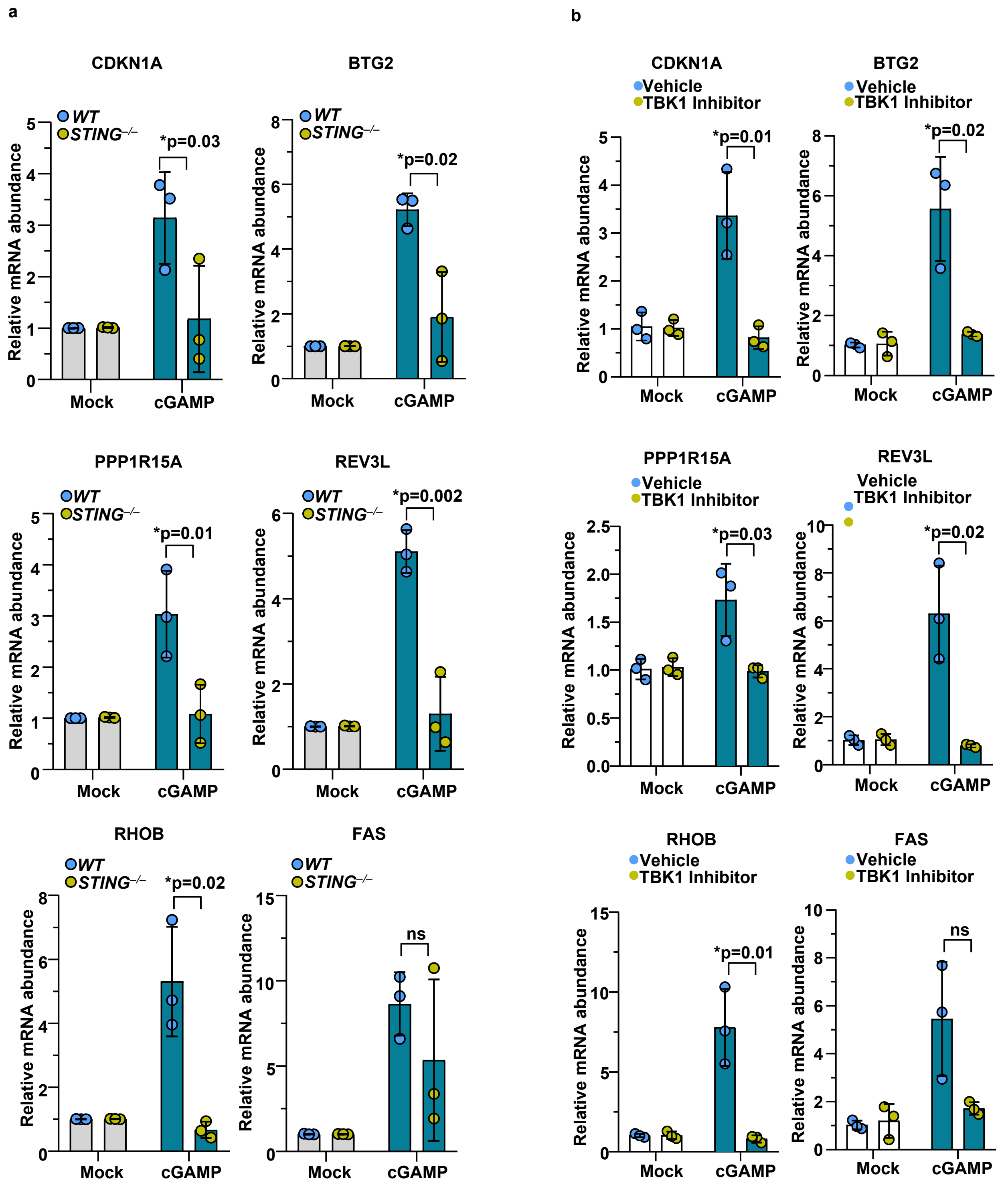

Supplementary Figure 6: A) Analysis of DNA Damage Response (DDR) gene expression of six DDR genes by quantitative real-time PCR (RT-qPCR) in WT and *STING*$^{-/-}$ THP1 cells stimulated with vehicle or cGAMP (n=3 independent experiments, data presented are mean ± s.d.; unpaired one-tailed *t* test; *p<0.05 indicates significance compared to respective groups; ns indicates not significant). B) Analysis of DNA Damage Response (DDR) gene expression of six DDR genes in WT THP1 cells treated with 10μM of the TBK1 Inhibitor MRT67307 for 2hrs followed by cGAMP treatment for 6hrs. (n=3 independent experiments, data presented are mean ± s.d.; unpaired one-tailed *t* test; *p<0.05 indicates significance compared to respective groups; ns indicates not significant).

Supplementary Figure 7

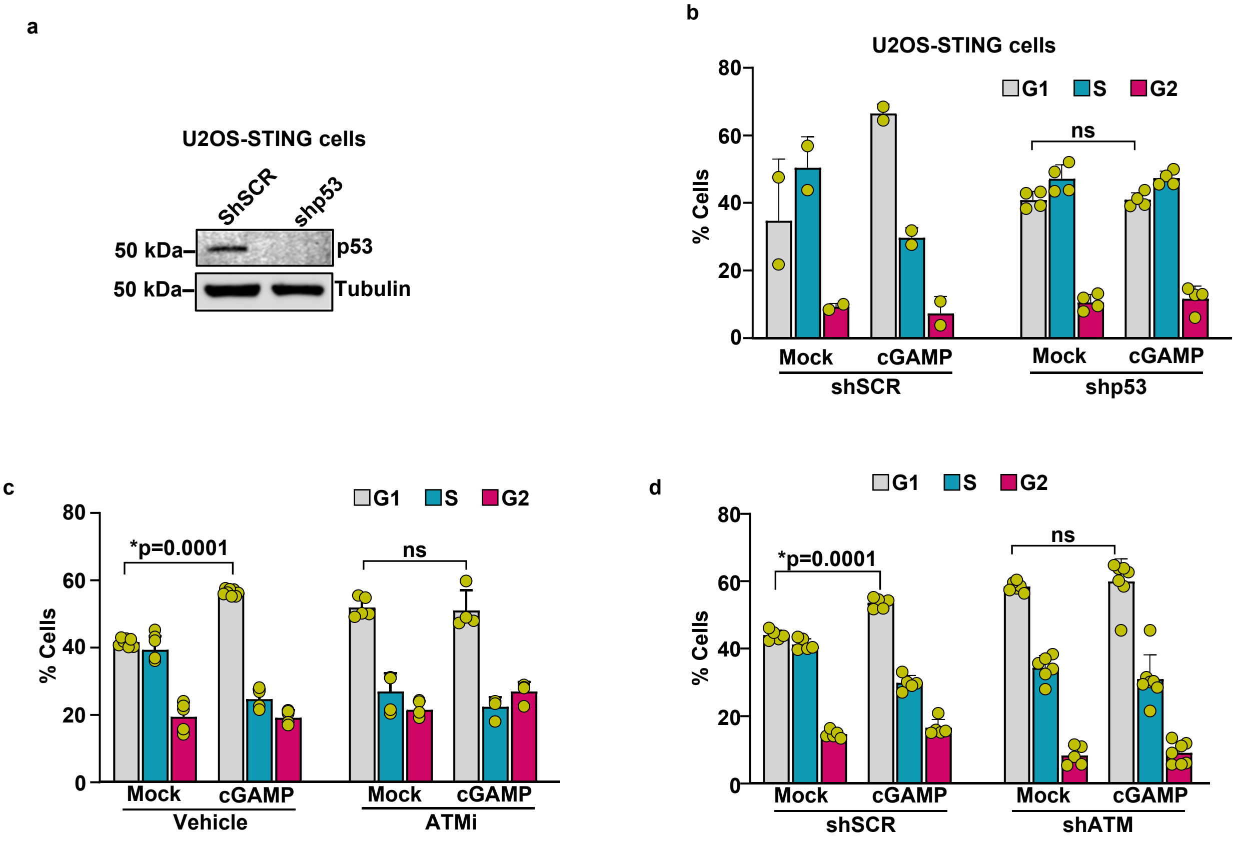

Supplementary Figure 7: A) Confirmation of p53 knockdown in shP53 U2OS-STING cells. Bands of interest from two immunoblotting assessment of knockdown efficiency in the cell line is presented. B) Cell cycle analysis (propidium iodide stain) of shSCR and shP53 U2OS-STING cells mock treated or stimulated with cGAMP for 24hrs (n= 4 independent cell culture replicates; data presented are mean ± s.d.; two-tailed unpaired *t* test; *p<0.05 indicates significance compared to respective groups; ns indicates not significant). C) Cell cycle analysis (propidium iodide stain) of WT THP1 cells pre-treated with 25µM of ATM inhibitor (KU-55933) then stimulated with mock treatment or cGAMP for 24hrs (n= 7 Vehicle group and N- 5 ATMi mock group, N=4 ATMi cGAMP group. Samples are from independent experiments; data presented are mean ± s.d.; two-tailed unpaired *t* test; *p<0.05 indicates significance compared to respective groups; ns indicates not significant). D) Cell cycle analysis (propidium iodide stain) of shSCR and shATM THP1 cells mock treated or stimulated with cGAMP for 24hrs (n= 5 for all shSCR group, N=6 for Mock +shATM group, and N=7 for cGAMP +shATM group. Samples are independenet cell culture replicates; data presented are mean ± s.d.; two-tailed unpaired *t* test; *p<0.05 indicates significance compared to respective groups; ns indicates not significant).

Supplementary Figure 8

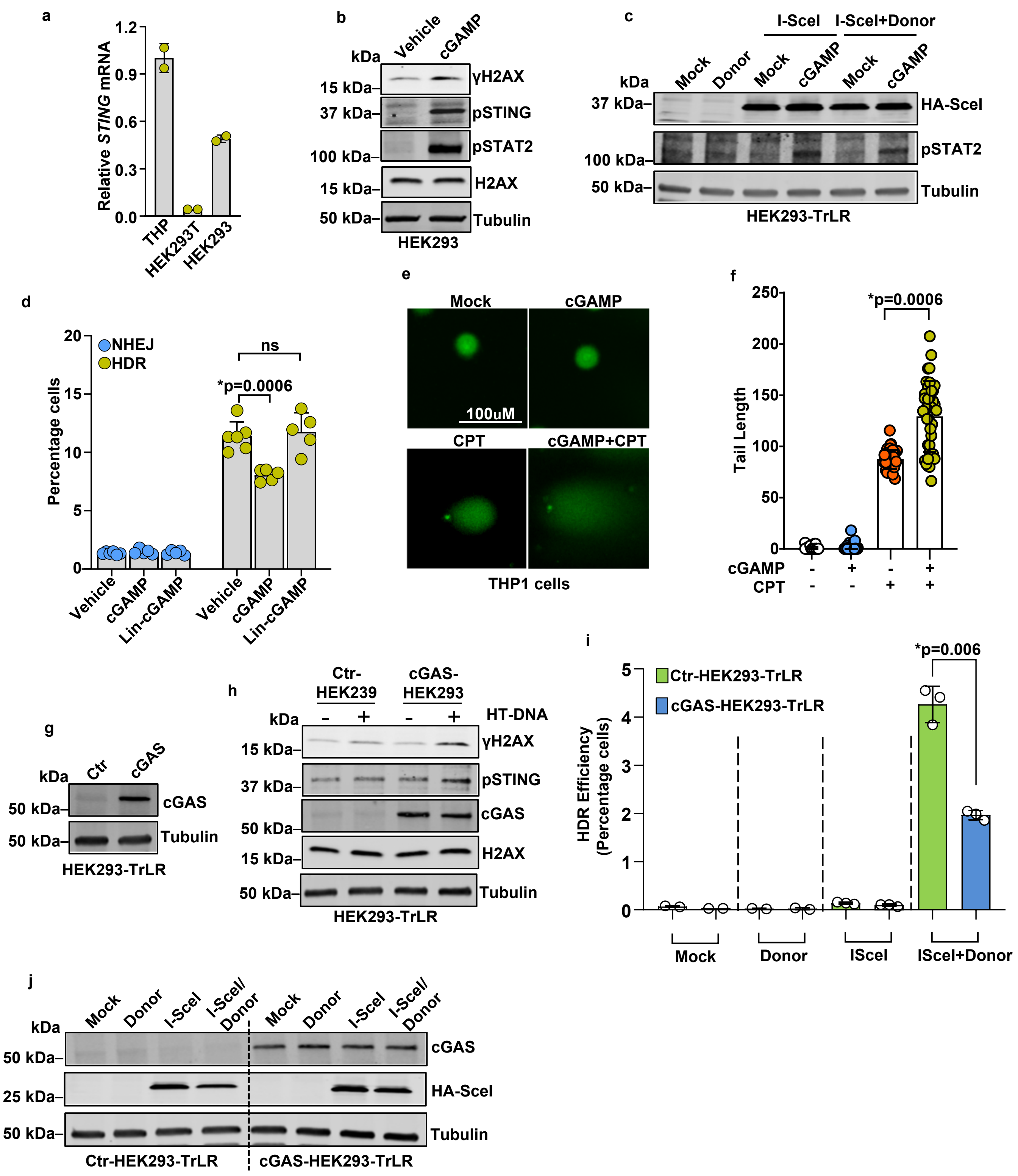

Supplementary Figure 8: A) The abundance of *STING* mRNA in the HEK293 cells used to generate HEK293-TrLR cells was measured by RT-qPCR. These relative abundances were calculated in reference to those in THP1 cells and HEK293T cells. 18S rRNA levels were used to normalize qPCR data (N=2 independent cell culture replicates, data presented are mean ± s.d.). B) To assess responsiveness to cGAMP, HEK293 cells transfected with cGAMP or vehicle were harvested (at 4hrs post transfection) and analyzed by immunoblotting for γH2AX, pSTING, and pSTAT2. Bands of interest from representative immunoblots from three independent experiments are shown. C) The HEK293-TrLR cells from the experiment described in Figure 7f, g were analyzed by immunoblotting for HA-SceI and phosphorylated STAT2 (pSTAT2) to ensure I-SceI expression and functional cGAMP signaling, respectively. Bands of interest from representative immunoblots from three independent experiments are shown. D) Quantification of flow cytometric analysis of HEK293-TrLR cells stimulated with vehicle, signaling incompetent linear cGAMP (Lin-cGAMP), or cGAMP and then transfected with I-SceI (NHEJ) or I-SceI with donor (HDR) (N=6 for vehicle groups and N=5 for cGAMP and Lin-cGAMP groups; samples are from independent biological replicates; data presented are mean ± s.d.; two-tailed unpaired *t* test; *p<0.025indicates significance compared to respective groups; ns indicates not significant). E) An alkaline comet assay was performed to assess DNA damage in THP1 cells stimulated with vehicle or cGAMP for 6hrs and then treated with 1μM camptothecin (CPT) for 16hrs. DNA (green) was visualized by staining with Vista Green DNA Dye. While the comet head is composed of intact DNA, tail consists of genetic fragments and has a length reflective of the amount of DNA damage the cell has sustained. Scale bar=100μm. Representative images from three independent experiments are shown. F) Comets for N=37 cells per condition were analyzed using OpenComet; quantification of comet tail length as a measure of DNA damage is presented. (data presented are mean ± s.d., two-tailed unpaired *t* test; *p<0.05 indicates significance compared to respective groups; ns indicates not significant) G) Immunoblots for cGAS in HEK293-TrLR cells stably expressing cGAS via lentiviral transduction. Bands of interest from two immunoblotting assessment of the ectopically expressed cGAS in the cell line is presented. H) HEK293-TrLR cells transduced with an empty vector (Ctr-HEK293-TrLR) or cGAS expression lentivirus (cGAS-HEK293-TrLR) were mock transfected or transfected with cGAS ligand HT-DNA (4ug per 6 well for 4hrs). Protein lysates from these cells were analyzed by immunoblotting for γH2AX, pSTING, and cGAS. Bands of interest from representative immunoblots from three independent experiments are shown. I) Quantification of HDR in Ctr-HEK293-TrLR and cGAS-HEK293-TrLR cells by flow cytometry as described in Fig. 7e (n=3 cell culture replicates, data presented are mean ± s.d.; two-tailed unpaired *t* test; *p<0.05 indicates significance compared to respective groups; ns indicates not significant). J) The Ctr-HEK293-TrLR and cGAS-HEK293-TrLR cells from the experiment described in (I) were analyzed by immunoblotting for HA-SceI and cGAS to ensure I-SceI and cGAS expression, respectively. Tubulin and total H2AX were used as loading controls for immunoblots as indicated. Bands of interest from representative immunoblots from three independent experiments are shown.

Supplementary Figure 9

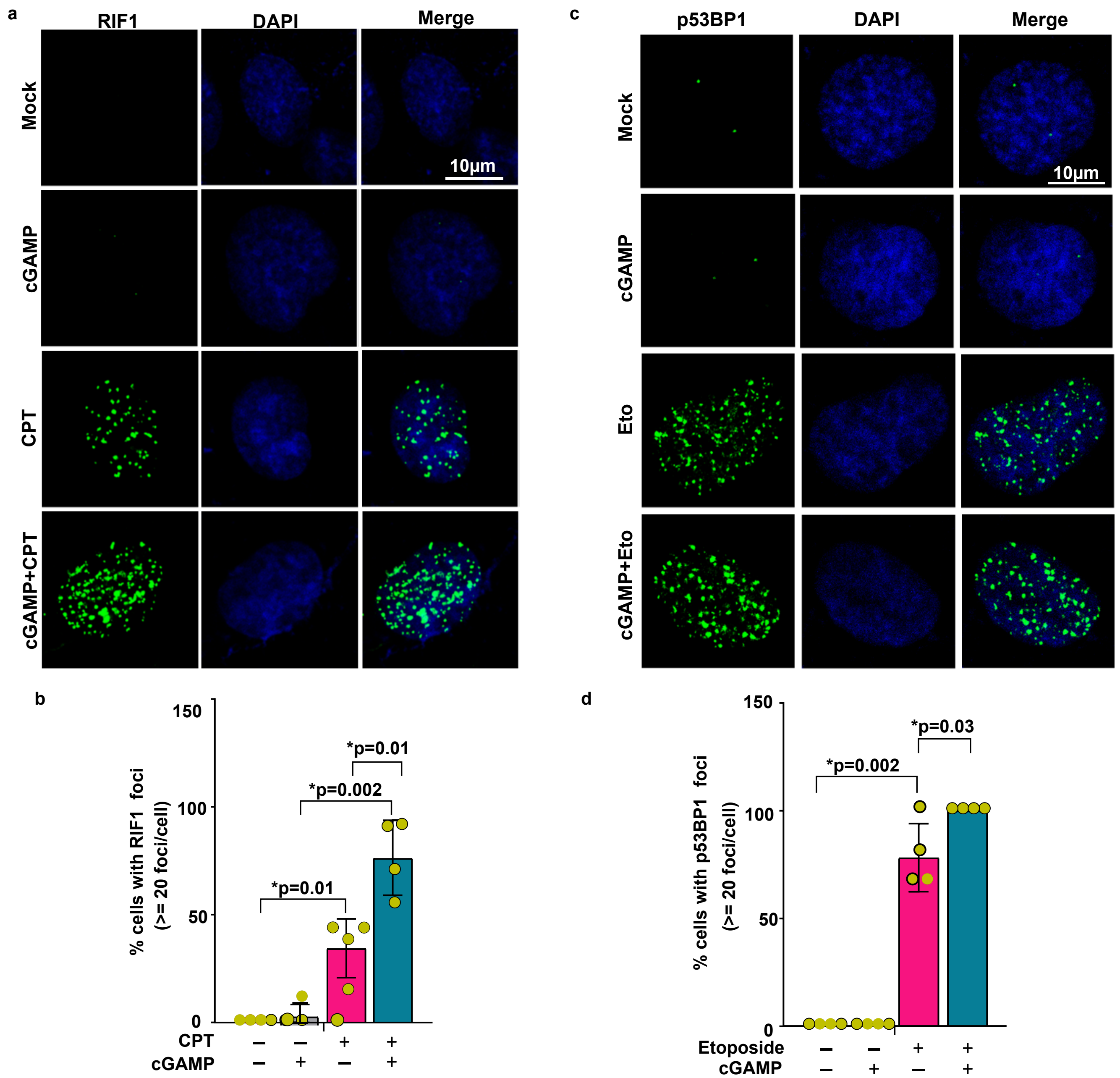

Supplementary Figure 9: A) Immunofluorescence of RIF1 in mock or CPT treated (5µM for 16hrs) U2OS-STING cells that were pretreated with mock or cGAMP. Scale bar =10µm. B) Quantification of RIF1 foci in CPT treated (5µM for 16hrs) U2OS-STING cells that were pretreated with mock or cGAMP. Each data point represents the percentage of cells with >20 foci/cell in a microscopic field (n=4 fields with over 100 cells collectively per condition; two-tailed unpaired *t* test; data presented are mean ± s.d.; *p<0.025 indicates significance compared to respective groups; ns indicates not significant; adjustments are made for multiple comparisons.). C) Immunofluorescence of phosphorylated P53BP1 (p53BP1) in mock or etoposide treated (40µM for 2 hrs) U2OS-STING cells that were pretreated with mock or cGAMP. Scale bar =10µm. D) Quantification of p53BP1 foci in etoposide treated (40 µM for 2 hrs) U2OS-STING cells that were pretreated with mock or cGAMP. Each data point represents the percentage of cells with >20 foci in a microscopic field (n=4 fields with over 100 cells collectively per condition; two-tailed unpaired *t* test; data presented are mean ± s.d.; *p<0.025 indicates significance compared to respective groups; ns indicates not significant; adjustments are made for multiple comparisons).

Supplementary Figure 10

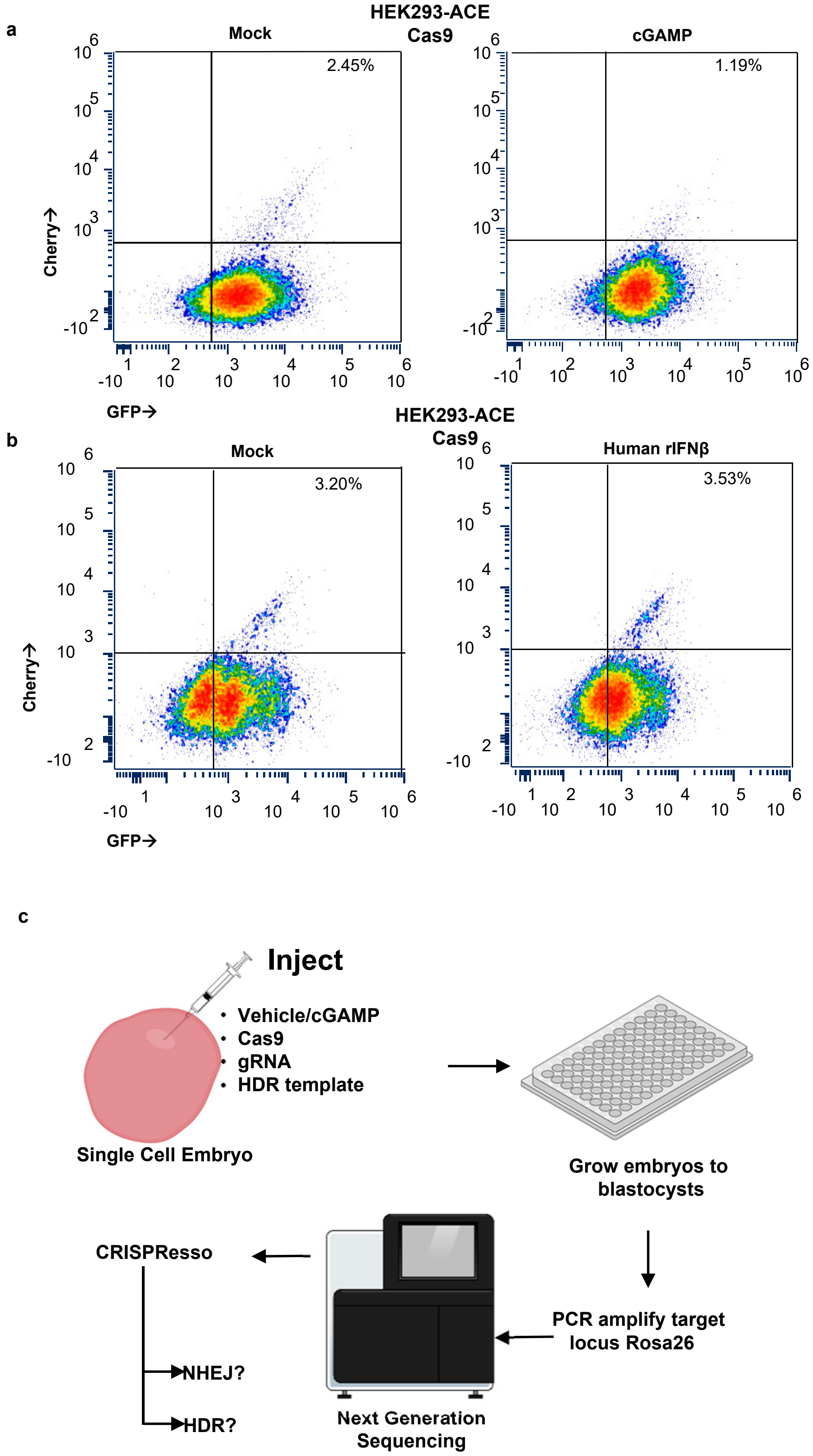

Supplementary Figure 10: A) Representative flow cytometric analyses of GFP positive HEK293-ACE CRISPR/Cas9 reporter cells "mock" stimulated or stimulated with "cGAMP" transfected with recombinant Cas9, gRNA, and donor template to repair DNA sequences encoding mutant non-fluorescent mCherry to functional fluorescent mCherry expression cassettes. B) Representative flow cytometric analyses of GFP positive HEK293-ACE CRISPR/Cas9 reporter assay with "mock" and human recombinant interferonβ (human rIFNβ) treated cells (50ng/ml for 6hrs). C) Schematic of the experimental design applied to examine the effect of cGAMP on CRISPR/Cas9-mediated gene editing in mouse embryos.

Supplementary Figure 11

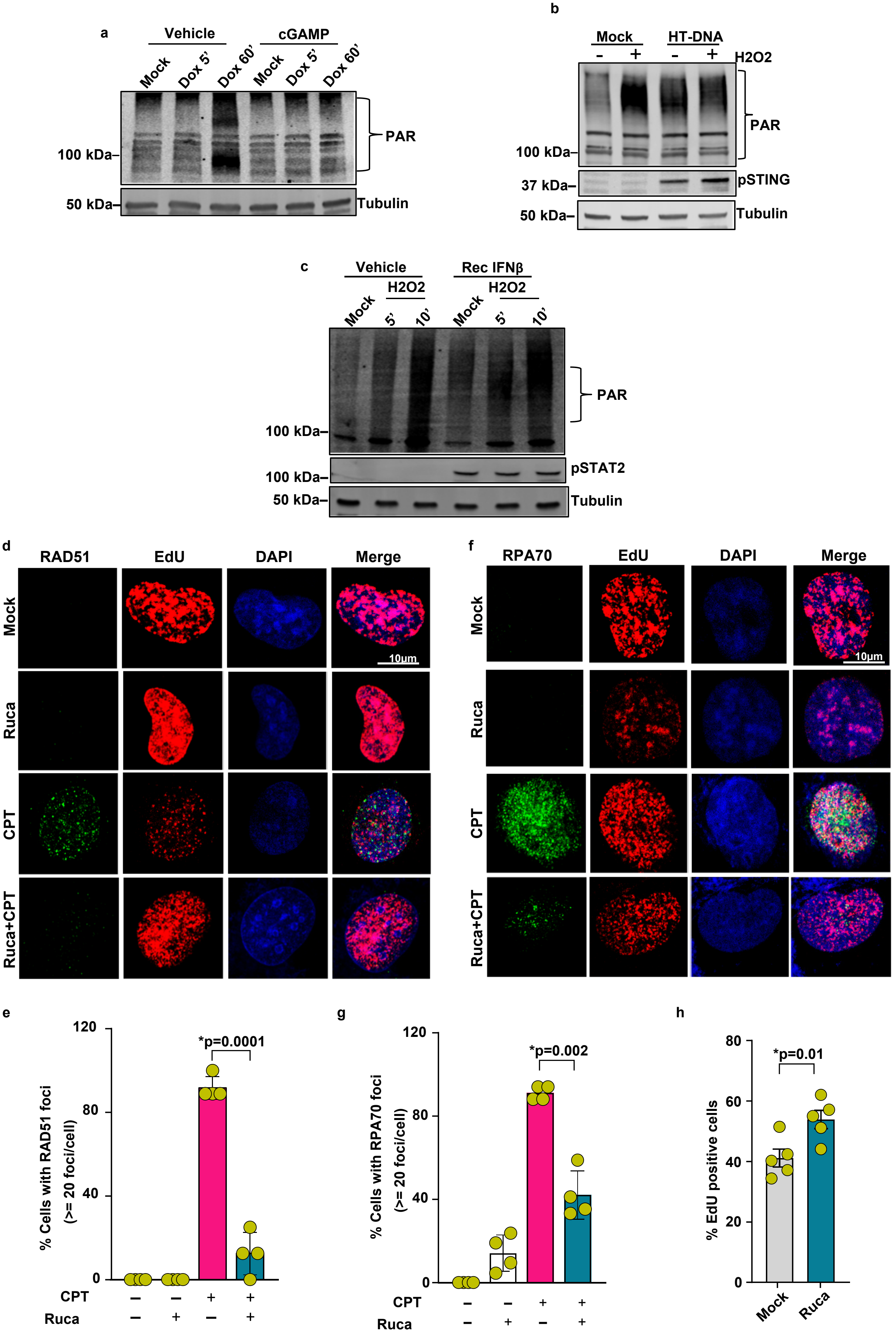

Supplementary Figure 11: A) Immunoblot of poly-ADP-ribosylated proteins (PAR) in WT THP1 cells treated with vehicle or cGAMP for 6hrs followed by 1μM Doxorubicin for the indicated time periods. Bands of interest from representative immunoblots from three independent experiments are shown. B) Immunoblots of poly-ADP-ribosylated proteins (PAR) and phosphorylated STING (pSTING) in WT THP1 cells transfected with vehicle or HT-DNA (4ug per 6 well for 4hrs) then treated with 250μM H_2O_2 for ten minutes. Bands of interest from representative immunoblots from three independent experiments are shown. C) Immunoblot of poly-ADP-ribosylated proteins (PAR) and phosphorylated STAT2 (pSTAT2) in WT THP1 cells that were pre-treated with human recombinant protein (Rec IFNβ, 50ng/ml for 6hrs) then challenged with H_2O_2 for the indicated time periods. Tubulin served as the loading control. Bands of interest from representative immunoblots from three independent experiments are shown. D) Immunofluorescence of RAD51 in mock or CPT treated (5μM for 16hrs) Edu^+ U2OS-STING cells that were preincubated with either vehicle or Rucaparib (Ruca) scale bar =10μm. E) Quantification of RAD51 foci in S-phase cells, each data point represents the percentage of EdU^+ cells with >20 foci in a microscopic field (n=4 fields with over 100 cells collectively per condition; data presented are mean ± s.d.; two-tailed unpaired t test; *$p<0.05$ indicates significance compared to respective groups; ns indicates not significant). F) Immunofluorescence of RPA70 in mock or CPT treated (5μM for 16hrs) Edu^+ U2OS-STING cells that were preincubated with either vehicle or Rucaparib (Ruca) scale bar =10μm. G) Quantification of RPA70 foci in S-phase cells, each data point represents the percentage of EdU^+ cells with >20 foci in a microscopic field (n=4 fields with over 100 cells collectively per condition; data presented are mean ± s.d.; two-tailed unpaired t test; *$p<0.05$ indicates significance compared to respective groups; ns indicates not significant). H) U2OS-STING cells incubated with vehicle or Rucaparib were analyzed to assess relative frequency of S phase by Edu incorporation assay. Cells incubated with Edu for 1hr prior to harvesting were stained for Edu incorporation using a Click-iT EdU assay. The percentages of cells with incorporated Edu as visualized by confocal microscopy are indicated in the graph. Each data point represents the percentage of cells in one image field (n=5 fields with over 100 cells collectively per condition; data presented are mean ± s.d.; two-tailed unpaired t test; *$p<0.05$ indicates significance compared to respective groups; ns indicates not significant)

Supplementary Figure 12

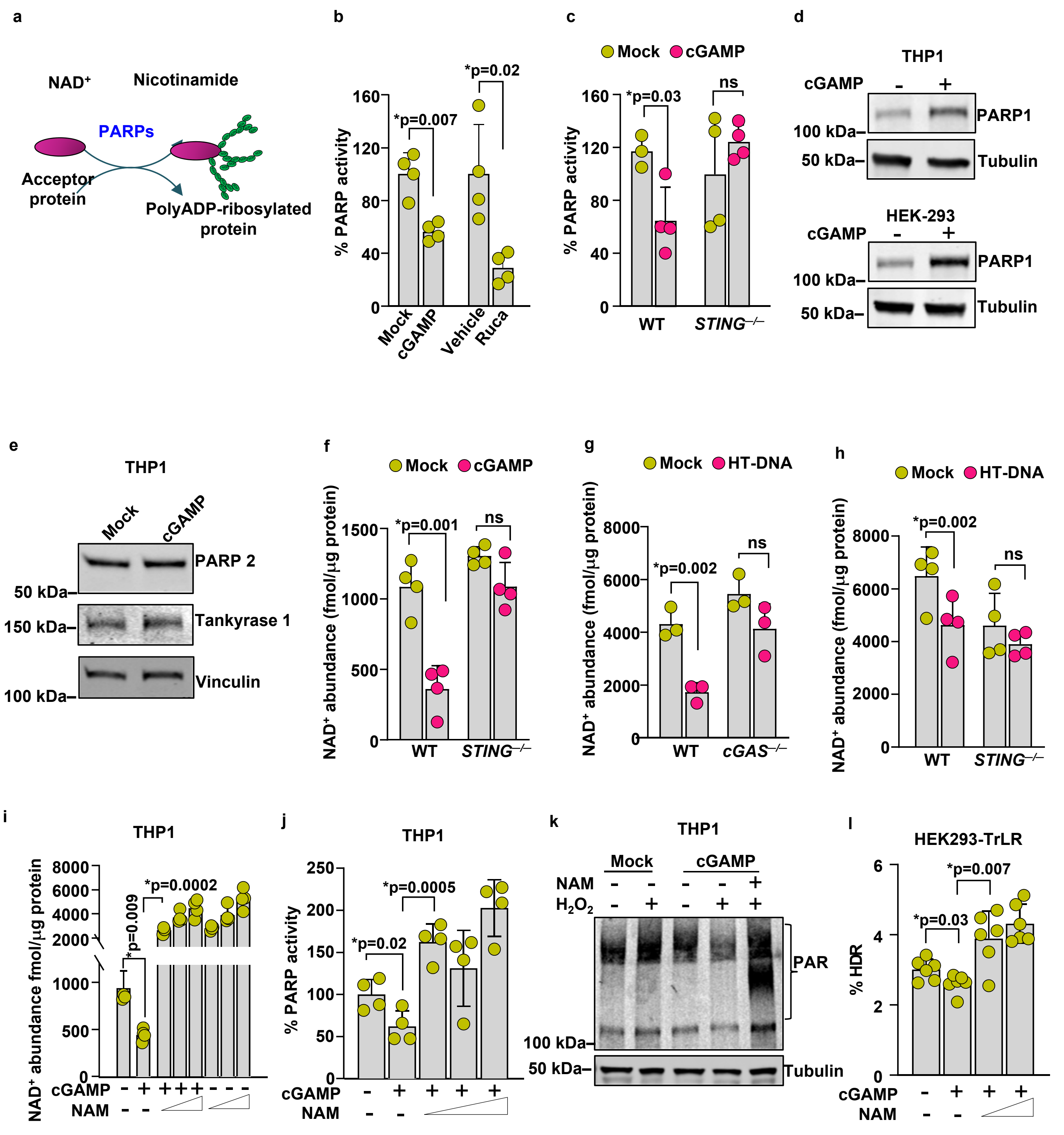

Supplementary Figure 12: cGAMP suppresses PARP enzymatic activity via decreasing cellular NAD^+ abundance.

A) Schematic of the PARP enzymatic reaction catalyzing synthesis ADP-ribose polymers on acceptor proteins; in this reaction ADP-ribose monomers are donated by NAD^+ molecules. B) PARP enzymatic activity levels in the lysates derived from THP1 cell mock treated or stimulated with cGAMP for 6 hrs, and incubated with vehicle or Rucaparib (10µM, for 6hr) is presented. (N=4 independent cell culture replicates, data presented are mean ± s.d.; two-tailed unpaired *t* test; *p<0.05 indicates significance compared to respective groups; ns indicates not significant) C) PARP enzymatic activity levels in the mock or cGAMP stimulated *WT* and *STING*$^{-/-}$ THP1 cells. (n=3 or 4 independent cell culture replicates, data presented are mean ± s.d.; two-tailed unpaired *t* test; *p<0.05 indicates significance compared to respective groups; ns indicates not significant). D) Immunoblot for PARP1 in mock or cGAMP-stimulated THP1 and HEK293 cells. Bands of interest from representative immunoblots from three independent experiments are shown. E) Immunoblot for PARP2 and Tankyrase -1 in mock or cGAMP-stimulated THP1 cells. Bands of interest from representative immunoblots from three independent experiments are shown. F) Measurement of NAD^+ abundance in mock or cGAMP stimulated WT and *STING*$^{-/-}$ THP1 cells. N=4 independent cell culture replicates, data presented are mean ± s.d.; two-tailed unpaired *t* test, *p<0.05 indicates significance compared to respective groups; ns indicates not significant. G) Measurement of NAD^+ abundance in the mock or HT-DNA (2ug/well) transfected *WT* and *cGAS*$^{-/-}$ differentiated THP1 cells. N=3 independent cell culture replicates, data presented are mean ± s.d.; two-tailed unpaired *t* test; *p<0.05 indicates significance compared to respective groups; ns indicates not significant. H) Measurement of NAD^+ abundance in the mock or HT-DNA (2ug/well) transfected *WT* and *STING*$^{-/-}$ differentiated THP1 cells. N=4 independent cell culture replicates, data presented are mean ± s.d.; two-tailed unpaired *t* test, *p<0.05 indicates significance compared to respective groups; ns indicates not significant. I) Measurement of NAD^+ abundance in mock or cGAMP stimulated THP1 cells that were preincubated with 2mM, 4mM and 8mM nicotinamide (NAM) for 18 hrs or media. N=4 independent cell culture replicates, data presented are mean ± s.d.; two-tailed unpaired *t* test; *p<0.025 indicates significance compared to respective groups; ns indicates not significant; adjustments are made for multiple comparisons. J) Measurement of PARP enzymatic activity levels in mock or cGAMP stimulated THP1 cells that were preincubated with 2mM, 4mM and 8mM nicotinamide (NAM) for 18 hrs or not. N=4 independent cell culture replicates, data presented are mean ± s.d.;, two-tailed unpaired *t* test; *p<0.025 indicates significance compared to respective groups; ns indicates not significant; adjustments are made for multiple comparisons. K) Immunoblot of poly-ADP-ribosylated proteins (PAR) in mock or cGAMP stimulated THP1 cells that were preincubated with 2mM nicotinamide (NAM) for 18 hrs or not and treated with 250µM H_2O_2 (+) for 10 minutes. Bands of interest from representative immunoblots from three independent experiments are shown. L) Quantification of HDR events in mock or cGAMP stimulated HEK293-TrLR cells that were preincubated with either 2mM and 4 mM nicotinamide (NAM) or not. N=6 independent cell culture replicates, data presented are mean ± s.d.; two-tailed unpaired *t* test; *p<0.025 indicates significance compared to respective groups; ns indicates not significant; adjustments are made for multiple comparisons.

Supplementary Figure 13

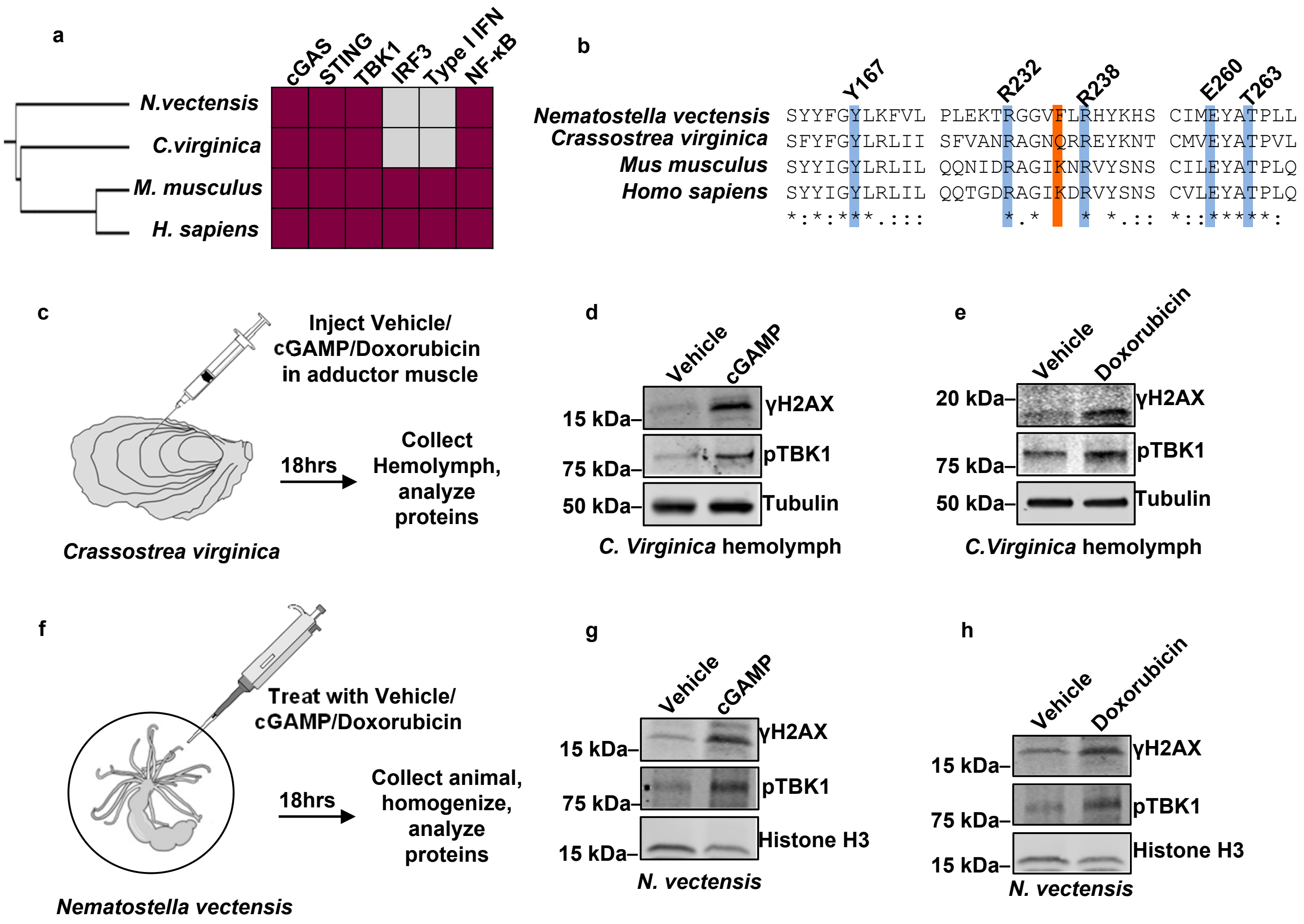

Figure 13: cGAMP activates DNA damage response in *Crassostrea virginica* and *Nematostella vectensis*. A) The phylogenetic tree was generated using https://phylot.biobyte.de/ with NCBI Taxonomy IDs as input data for *Nematostella vectensis*, *Crassostrea virginica*, *Mus musculus*, and *Homo sapiens*. The presence or absence of homologs of cGAS, STING, TBK1, downstream IRF3, type I Interferon, and NF-κB in each species are indicated by maroon and gray boxes respectively[73, 74, 75]. B) Phylogenetic alignment of STING proteins, * (asterisk) indicates fully conserved, : (colon) indicates strongly similar properties, . (period) indicates weakly similar properties. Key cGAMP interacting residues are highlighted in blue (conserved) or orange (divergent). C-E) Doxorubicin, cGAMP, or vehicle (water) was injected in the adductor muscle of *C. virginica* and hemolymph was collected. Whole cell lysates from hemocytes were analyzed by immunoblotting to assess the phosphorylation status of H2AX (γH2AX) and TBK1 (pTBK1). Tubulin was used as a loading control for immunoblots. Bands of interest from representative immunoblots from three independent experiments are shown. F-H) *N. vectensis* were immersed in digitonin permeabilization solution supplemented with cGAMP or vehicle and then transferred to maintenance sea salt water. Protein lysates generated by homogenizing whole animals via sonication were analyzed by immunoblotting for phosphorylated H2AX (γH2AX) and TBK1 (pTBK1). Histone H3 was used as loading control for immunoblots. *N. vectensis* were immersed in maintenance sea salt water with or without doxorubicin. Protein lysates were prepared as above and immunoblotted for γH2AX and pTBK1 as described above. Bands of interest from representative immunoblots from three independent experiments are shown.

Supplementary Figure 14

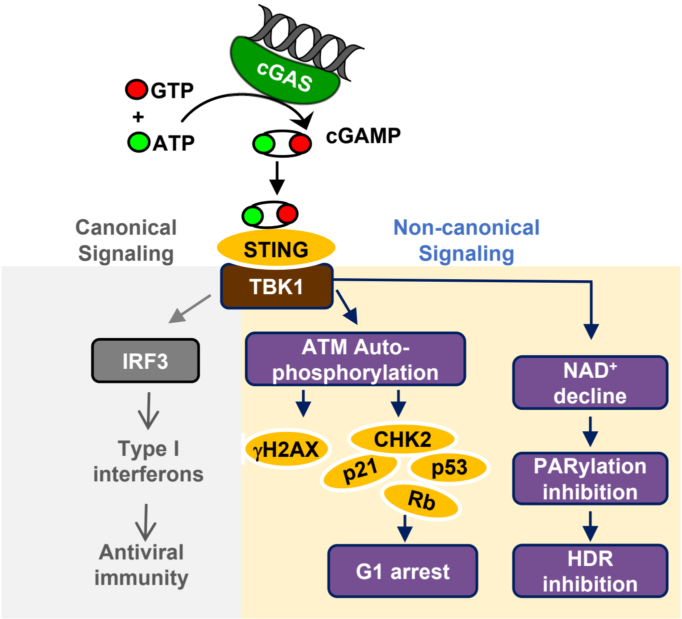

Supplementary Figure 14: Model showing the non-canonical activity of cGAMP as it pertains to the activation of DNA Damage Response signaling via the STING-TBK1 axis and inhibition of PARylation activity which functionally impede cell cycle progression and homology directed repair.

Supplementary Figure 15

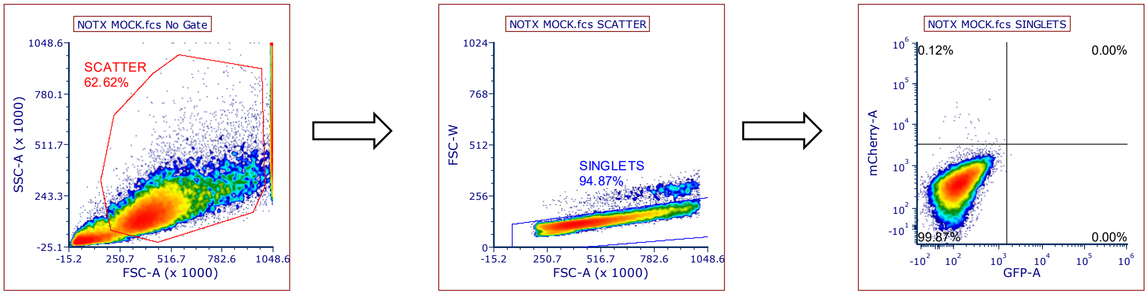

Supplementary Figure 15A:Gating strategy for sorting GFP and mCherry positive cells in TrLR and ACE reporter assays

The above figure panels exemplify the gating strategy for our the experiments in TrLR and ACE reporter assays: To quantify the DNA repair events, ~10,000-30,000 cells were acquired on an Attune NxT cytometer and analyzed using FCS Express software. Single cell events were determined by gating on the area against the width of the forward scatter pulse signal. Debris removal was performed by excluding events with very low forward and side scatter. For determining % of GFP and mCherry positive cells, we established positive and negative populations using mock transfections as negative controls and GFP or mCherry transfected samples as Fluorescence Minus One controls.

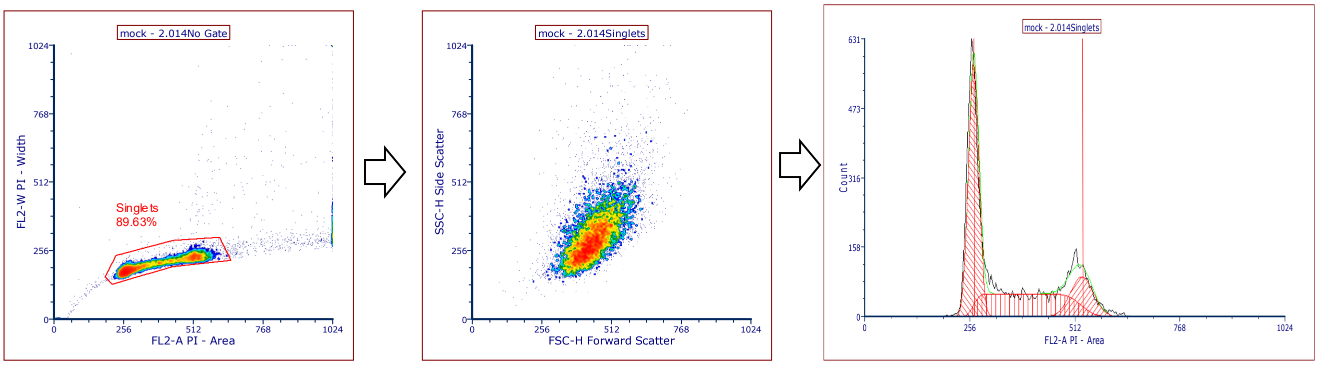

Supplementary Figure 15B:Gating strategy for sorting PI stained THP1 cells for cell cycle analysis

The above figure panels exemplify the gating strategy for our flowcytometry analysis to examine cell cycle analysis: To determine percentage of cell population in various cell cycle stage, ~10,000-30,000 cells were acquired on an Attune NxT cytometer and analyzed using FCS Express software. Cell cycle analysis was performed on propidium iodide stained cells and single cell events were determined by gating on the area against the width of the propidium iodide pulse signal. DNA cell cycle modeling and fit was performed using the Multicycle AV plugin (Phoenix Flow Systems).

Supplementary Table 1

Antibodies	Source	Dilution
Phospho-Histone H2AX	Cell Signaling Cat# 9718	WB 1:1000
Phospho-Chk2	Cell Signaling Cat# 2197	WB 1:1000
Phospho-ATM	Cell Signaling Cat# 5883	WB 1:1000
Phospho-ATR	Cell Signaling Cat# 2853	WB 1:1000
Phospho-STING (for mouse)	Cell Signaling Cat# 72971	WB 1:1000
Phospho-STING (for human)	Cell Signaling Cat# 19781	WB 1:1000
STING	Cell Signaling Cat# 13647	WB 1:1000
Phospho-STAT2	Millipore-Sigma Cat# 07-224	WB 1:250
STAT2	Thermo Fisher Cat# 701105	WB 1:1000
Phospho-TBK1	Abcam Cat# ab109272	WB 1:1000
TBK1	Cell Signaling Cat# 3013	WB 1:1000
β-Actin	Millipore-Sigma Cat# A2228-100UL	WB 1:5000
Tubulin	Millipore-Sigma Cat# T6199-200UL	WB 1:5000
HA-Tag	Cell Signaling Cat# 2367	WB 1:1000
IRF3	Novas Biologicals Cat# NBP1-78769	WB 1:1000
Phospho-NF-κB p65	Cell Signaling Cat# 3033	WB 1:1000
cGAS (for mouse)	Cell Signaling Cat# 31659	WB 1:1000
TBP	Abcam Cat# ab51841	WB 1:1000
Histone H2A.X Antibody	Cell Signaling Cat# 2595	WB 1:1000
Phospho-Rb (Ser807/811)	Cell Signaling Cat# 8516	WB 1:1000
Phospho-Chk1 (Ser345)	Cell Signaling Cat# 2348	WB 1:1000
pADPr Antibody (10H)	Santa Cruz Biotechnology Cat# sc-56198	WB 1:200
PARP (46D11) (For IP)	Cell Signaling Cat# 9532	4µg per reaction
PARP1 Antibody (For WB)	Cell Signaling Cat# 9542	WB 1:1000
PARP2 (For WB)	Thermo Fisher Scientific Cat# MA5-34728	WB 1:1000
Tankyrase-1 (For WB)	Bethyl Laboratories, Inc., Cat# A302-399A-T	WB 1:1000
Anti-RAD 51 antibody	Bio-academia Cat# 70-001	IF 1:6000
RPA70/RPA1 Antibody	Cell Signaling Cat# 2267	IF 1:50
Phospho-Histone H2AX	Cell Signaling Cat# 9718	IF 1:500
Anti-TBK1 Antibody	Atlas Antibodies Cat#HPA045797	IF 1:200
HA-Tag	Cell Signaling Cat# 2367	IF 1:100
IRDye® 800CW Donkey anti-Mouse IgG	LI-COR Biosciences Cat# 926-32212	WB 1:15,000
IRDye® 800CW Donkey anti-Rabbit IgG	LI-COR Biosciences Cat#926-32213	WB 1:15,000
Donkey anti-Rabbit Alexa fluor 488	Thermofisher Cat#A-21206	IF 1:1000
Donkey anti-Mouse, Alexa Fluor 555	Thermofisher Cat#A-31570	IF 1:2000

Supplementary Table 2

Primers, sgRNA, shRNA, ssDNA donor templates	Source	Catalogue#
qPCR hIFNβ forward GCGACACTGTTCGTGTTGTC	IDT	NA
qPCR hIFNβ Reverse GCCTCCCATTCAATTGCCAC	IDT	NA
qPCR hCDKN1A-forward AGTCAGTTCCTTGTGGAGCC	IDT	NA
qPCR hCDKN1A-reverse CATTAGCGCATCACAGTCGC	IDT	NA
qPCR hPLK3-forward AGAAGTGCGCTACTACCTGC	IDT	NA
qPCR hPLK3-reverse TCAGGTCAGCCGTCTCAAAG	IDT	NA
qPCR hRAD9A-forward GAGCCCTTTTCCCAGAGTTACA	IDT	NA
qPCR hRAD9A-reverse AGAGAAGGGCAGAACAGCCT	IDT	NA
qPCR hREV3L-forward ACTACTACATGGCCAGCCCG	IDT	NA
qPCR hREV3L-reverse TGCTTTTATGTGGCTTGTCTTGG	IDT	NA
qPCR hRHOB-forward CAGTAAGGACGAGTTCCCCG	IDT	NA
qPCR hRHOB-reverse GTCCACCGAGAAGCACATGA	IDT	NA
qPCR hPPP1R15A-forward CTCTGGCAATCCCCCATACC	IDT	NA
qPCR hPPP1R15A-reverse TCTCGCTCACCATACATGCC	IDT	NA
qPCR hTNFRSF10B-forward TTCCCTACCGCCATGGAACA	IDT	NA
qPCR hTNFRSF10B-reverse GGGGAGCTAGGTCTTGTTGG	IDT	NA
qPCR hBTG2-forward GGTAACGCTGTCTTGTGGAC	IDT	NA
qPCR hBTG2-reverse CGGGAAACCAGTGGTGTTTG	IDT	NA
qPCR hFAS-forward ACCCGGACCCAGAATACCAA	IDT	NA
qPCR hFAS-reverse AAGAAGACAAAGCCACCCCA	IDT	NA
qPCR hKIF20A-forward ATTTGGGGTCTGTGGTACGC	IDT	NA
qPCR hKIF20A-reverse ACAAGGGCCTAACCCTCAAG	IDT	NA
qPCR hRPS27L-forward CTTGCTAGCTGTGTGGGCT	IDT	NA
qPCR hRPS27L-reverse CTGAGCATGGCTGAAAACCG	IDT	NA
qPCR hPCNA-forward AGGCTCTAGCCTGACAAATGC	IDT	NA
qPCR hPCNA-reverse AAGTCTAGCTGGTTTCGGCT	IDT	NA
qPCR h18s forward CGCAGCTAGGAATAATGGAATAGG	IDT	NA
qPCR h18s reverse GCCTCAGTTCCGAAAACCAA	IDT	NA
qPCR huIFNAR1-forward GCGCGAACATGT AAC TGG TG	IDT	NA
qPCR huIFNAR1-reverse ATTCCCGACAGA CTC ATC GC	IDT	NA
qPCR huSTING forward ATATCTGCGGCTGATCCTGC	IDT	NA
qPCR huSTING-reverse GGTCTGCTGGGGCAGTTTAT	IDT	NA
qPCR mIFNB-forward CGTGGGAGATGTCCTCAACT	IDT	NA
qPCR mIFNB-reverse CCTGAAGATCTCTGCTCGGAC	IDT	NA
qPCR m18s-forward TTC GTA TTG CGC CGC TGA A	IDT	NA
qPCR m18S-reverse CTT TCG CTC TGG TCC GTC TT	IDT	NA
hE2F1-forward AGCTCATTGCCAAGAAGTCCA	IDT	NA
hE2F1-reverse AGGGTCTGCAATGCTACGAA	IDT	NA
hE2F2-forward GGGTAGGCAGGGGAATGTTT	IDT	NA
hE2F2-reverse AGTTGCCAACAGCACGGATA	IDT	NA

Supplementary Table 2 (continued)

Primers, sgRNA, shRNA, ssDNA donor templates	Source	Catalogue#
qPCR hCCNA2-forward ACCATTCATGTGGATGAAGCAG	IDT	NA
qPCR hCCNA2-reverse ACACTCACTGGCTTTTCATCTT	IDT	NA
qPCR hp107-forward GGACATCTTCCCCTGATGCC	IDT	NA
qPCR hp107-reverse TCTTAGCACTCCCTGCGGTA	IDT	NA
qPCR hE2F3-forward GAAATGCCCTTACAGCAGCAG	IDT	NA
qPCR hE2F3-reverse TGGTGAGCAGACCAAGAGAC	IDT	NA
qPCR hDHFR-forward AGAATGACCACAACCTCTTCAGT	IDT	NA
qPCR hDHFR-reverse TGCCACCAACTATCCAGACC	IDT	NA
qPCR hMYC-forward TACAACACCCGAGCAAGGAC	IDT	NA
qPCR hMYC-reverse CTAACGTTGAGGGGCATCGT	IDT	NA
qPCR hPCNA-forward AGGCTCTAGCCTGACAAATGC	IDT	NA
qPCR hPCNA-reverse AAGTCTAGCTGGTTTCGGCT	IDT	NA
qPCR hKIF20A-forward ATTTGGGGTCTGTGGTACGC	IDT	NA
qPCR hKIF20A-reverse ACAAGGGCCTAACCCTCAAG	IDT	NA
qPCR hRPS27L-forward CTTGCTAGCTGTGTGGGCT	IDT	NA
qPCR hRPS27L-reverse CTGAGCATGGCTGAAAACCG	IDT	NA
qPCR hHIST1H4B-forward CCGAAAAGTGCTGCGGGATA	IDT	NA
qPCR hHIST1H4B-reverse GAAACACCTTGAGAACGCCAC	IDT	NA
qPCR hGADD45A-forward GCTGCGAGAACGACATCAAC	IDT	NA
qPCR hGADD45A-reverse TCCATGTAGCGACTTTCCCG	IDT	NA
qPCR hCDC2 Forward GGGCTACCCGATTGGTGAAT	IDT	NA
qPCR hCDC2 Reverse AGGAACCCCTTCCTCTTCACT	IDT	NA
sgRNA TMEM173 GAACCAAGGCTGCCTTC	ABMgood	K2402705
sgRNA Scrambled GCACTCACATCGCTACATCA	ABMgood	K010
shRNA TBK1<br>CCGGGCGGCAGAGTTAGGTGAAATTCTCGAGAATTTCACCTA<br>ACTCTGCCGCTTTTTG	Sigma	TRCN0000<br>314840
shRNA IFNAR<br>CCGGGCCAAGATTCAGGAAATTATTCTCGAGAATAATTTCCT<br>GAATCTTGGCTTTTTG	Sigma	TRCN0000<br>368990
shRNA STAT2<br>CCGGGACTGAAATCATCCGCCATTACTCGAGTAATGGCGGAT<br>GATTTCAGTCTTTTTG	Sigma	TRCN0000<br>368990
shRNA p53<br>CCGGCGGCGCACAGAGGAAGAGAATCTCGAGATTCTCTTCCT<br>CTGTGCGCCGTTTTT	Sigma	TRCN0000<br>003753
sgRNA Rosa26_NcoI<br>ACTCCAGTCTTTCTAGAAGATGG	IDT	NA
ssDonor_Rosa26_Nco1<br>TCTGAGGACCGCCCTGGGCCTGGGAGAATCCCTTCCCCCTCT<br>TCCCTCGTGATCTGCAACTCCAGTCTTTCTAGAccATGGGCGG<br>GAGTCTTCTGGGCAGGCTTAAAGGCTAACCTGGTGTGTGGGC<br>GTTGTCCTGCAGGGGAATTGAACAG	IDT	NA

Supplementary Table 2 (continued)

Primers, sgRNA, shRNA, ssDNA donor templates	Source	Catalogue#
sgRNA cGAS(GS198AA)<br>GGTGTGGAGCAGCTGAACACTGG	IDT	NA
ssDonor cGAS(GS198AA)<br>GAATAAAGTTGTGGAACGCCTGCTGCGCAGAATGCAGAAAC<br>GGGAGTCGGAGTTCAAAGGTGTGGAGCAGCTGAACACTgccgc<br>cTACTATGAACATGTGAAGGTGAGCGTCAAGACCTGCTGGAG<br>GGGCTCCGGCCCCACTCCTCACTTGCCTCCTCA	IDT	NA
mCherry ssDNA donor template (ACE reporter)<br>GGCGAGGGCCGCCCCTACGAGGGCACCCAGACCGCCAAGCT<br>GAAGGTGACCAAGGGTGGCCCCTTACCCTTCGCCT<br>GGGACATCCTGTCCCCTCAGTTCATGTACGGCTCCAAGGCCT<br>ACGTGAAGCACC	IDT	NA
mCherry+43 gRNA (ACE reporter)<br>TGGCCCCTCACCCTTCGCCT	IDT	NA
