# Supplementary material for: A non-canonical, interferon-independent signaling activity of cGAMP triggers DNA damage response signaling
Source: Nat Commun. 2021 Oct 27;12:6207. doi: 10.1038/s41467-021-26240-9 (PMC8551335; doi:10.1038/s41467-021-26240-9)
Supplement: Supplementary file 2 — Reporting Summary [file 41467_2021_26240_MOESM2_ESM.pdf]

nature portfolio

Corresponding author(s): Nagaraj Kerur

Last updated by author(s): Sep 6, 2021

Reporting Summary

Nature Portfolio wishes to improve the reproducibility of the work that we publish. This form provides structure for consistency and transparency in reporting. For further information on Nature Portfolio policies, see our Editorial Policies and the Editorial Policy Checklist.

Statistics

For all statistical analyses, confirm that the following items are present in the figure legend, table legend, main text, or Methods section.

n/a	Confirmed	
☐	☒	The exact sample size (*n*) for each experimental group/condition, given as a discrete number and unit of measurement
☐	☒	A statement on whether measurements were taken from distinct samples or whether the same sample was measured repeatedly
☐	☒	The statistical test(s) used AND whether they are one- or two-sided<br>*Only common tests should be described solely by name; describe more complex techniques in the Methods section.*
☒	☐	A description of all covariates tested
☐	☒	A description of any assumptions or corrections, such as tests of normality and adjustment for multiple comparisons
☐	☒	A full description of the statistical parameters including central tendency (e.g. means) or other basic estimates (e.g. regression coefficient) AND variation (e.g. standard deviation) or associated estimates of uncertainty (e.g. confidence intervals)
☐	☒	For null hypothesis testing, the test statistic (e.g. *F*, *t*, *r*) with confidence intervals, effect sizes, degrees of freedom and *P* value noted<br>*Give P values as exact values whenever suitable.*
☒	☐	For Bayesian analysis, information on the choice of priors and Markov chain Monte Carlo settings
☒	☐	For hierarchical and complex designs, identification of the appropriate level for tests and full reporting of outcomes
☒	☐	Estimates of effect sizes (e.g. Cohen's *d*, Pearson's *r*), indicating how they were calculated

Our web collection on statistics for biologists contains articles on many of the points above.

Software and code

Policy information about availability of computer code

Data collection	Flow cytometer (Attune NxT), WesternBlot (Odyssey CLx, Licor), qPCR(QuantstudioFlex 7, ThermoFisfer Scientific) and All images were obtained by confocal microscope system (Nikon, C2).
Data analysis	GraphPad Prism 7 software, Microsoft Excel, OpenComet v1.3 software (in ImageJ), FCS Express and Multicycle AV plugin (Phoenix Flow Systems), online tool MUSCLE (EBI), CRISPResso, LICOR image studio, PhyloT

For manuscripts utilizing custom algorithms or software that are central to the research but not yet described in published literature, software must be made available to editors and reviewers. We strongly encourage code deposition in a community repository (e.g. GitHub). See the Nature Portfolio guidelines for submitting code & software for further information.

Data

Policy information about availability of data

All manuscripts must include a data availability statement. This statement should provide the following information, where applicable:

- Accession codes, unique identifiers, or web links for publicly available datasets
- A description of any restrictions on data availability
- For clinical datasets or third party data, please ensure that the statement adheres to our policy

Authors can confirm that all relevant data are included in the paper and/or its supplementary information files.

Field-specific reporting

Please select the one below that is the best fit for your research. If you are not sure, read the appropriate sections before making your selection.

☒ Life sciences ☐ Behavioural & social sciences ☐ Ecological, evolutionary & environmental sciences

For a reference copy of the document with all sections, see nature.com/documents/nr-reporting-summary-flat.pdf

Life sciences study design

All studies must disclose on these points even when the disclosure is negative.

Sample size	No statistical methods were used to predetermine sample sizes. Sample sizes were selected based literature (PMID: 29176737 and 23258412).
Data exclusions	For determining the frequency of CRISPR/Cas9-mediated gene editing outcomes (NHEJ, HDR) in mouse embryos (Figure 8f), samples with fewer than 100 reads were excluded from analysis. For other experiments, no exclusion of data was made.
Replication	All experimental data was reliably reproduced in multiple independent experiments as indicated in the figure legends.
Randomization	Mice/cell lines were assigned into groups according to genotype of interest. Randomization was done generically.
Blinding	Flowcytometry and NGS-based determination of DNA repair were performed in blinded fashion. For remaining experiments blinding was not meaningful as same investigator performed the experiments and analyzed the data.

Reporting for specific materials, systems and methods

We require information from authors about some types of materials, experimental systems and methods used in many studies. Here, indicate whether each material, system or method listed is relevant to your study. If you are not sure if a list item applies to your research, read the appropriate section before selecting a response.

Materials & experimental systems

n/a	Involved in the study	
☐	☒	Antibodies
☐	☒	Eukaryotic cell lines
☒	☐	Palaeontology and archaeology
☐	☒	Animals and other organisms
☒	☐	Human research participants
☒	☐	Clinical data
☒	☐	Dual use research of concern

Methods

n/a	Involved in the study	
☒	☐	ChIP-seq
☐	☒	Flow cytometry
☒	☐	MRI-based neuroimaging

Antibodies

Antibodies used

Sources and usage details of all the antibodies used in the study are described in the methods section and in Table 1.
• Phospho-Histone H2AX, Cell Signaling Cat# 9718, WB 1:1000
• Phospho-Chk2, Cell Signaling Cat# 2197, WB 1:1000
• Phospho-ATM, Cell Signaling Cat# 5883, WB 1:1000
• Phospho-ATR, Cell Signaling Cat# 2853, WB 1:1000
• Phospho-STING (for mouse), Cell Signaling Cat# 72971, WB 1:1000
• Phospho-STING (for human), Cell Signaling Cat# 19781, WB 1:1000
• STING, Cell Signaling Cat# 13647, WB 1:1000
• Phospho-STAT2, Millipore-Sigma Cat# 07-224, WB 1:250
• STAT2, Thermo Fisher Cat# 701105, WB 1:1000
• Phospho-TBK1, Abcam Cat# ab109272, WB 1:1000
• TBK1, Cell Signaling Cat# 3013, WB 1:1000
• Beta-Actin, Millipore-Sigma Cat# A2228-100UL, WB 1:5000
• Tubulin, Millipore-Sigma Cat# T6199-200UL, WB 1:5000
• HA-Tag, Cell Signaling Cat# 2367, WB 1:1000
• IRF3, Novas Biologicals Cat# NBP1-78769, WB 1:1000
• Phospho-NF-?B p65, Cell Signaling Cat# 3033, WB 1:1000
• cGAS (for mouse), Cell Signaling Cat# 31659, WB 1:1000
• TBP, Abcam Cat# ab51841, WB 1:1000
• Histone H2A.X Antibody, Cell Signaling Cat# 2595, WB 1:1000

- Phospho-Rb (Ser807/811), Cell Signaling Cat# 8516, WB 1:1000
- Phospho-Chk1 (Ser345), Cell Signaling Cat# 2348, WB 1:1000
- pADPr Antibody (10H), Santa Cruz Biotechnology Cat# sc-56198, WB 1:200
- PARP (46D11) (For IP), Cell Signaling Cat# 9532, 4µg per reaction
- PARP1 Antibody (For WB), Cell Signaling Cat# 9542, WB 1:1000
- PARP2 (For WB), Thermo Fisher Scientific Cat# MA5-34728, WB 1:1000
- Tankyrase-1 (For WB), "Bethyl Laboratories, Inc., Cat# A302-399A-T", WB 1:1000
- Anti-RAD 51 antibody, Bio-academia Cat# 70-001, IF 1:6000
- RPA70/RPA1 Antibody, Cell Signaling Cat# 2267, IF 1:50
- Phospho-Histone H2AX, Cell Signaling Cat# 9718, IF 1:500
- Anti-TBK1 Antibody, Atlas Antibodies Cat#HPA045797, IF 1:200
- HA-Tag, Cell Signaling Cat# 2367, IF 1:100
- IRDye® 800CW Donkey anti-Mouse IgG, LI-COR Biosciences Cat# 926-32212, "WB 1:15, 000"
- IRDye® 800CW Donkey anti-Rabbit IgG, LI-COR Biosciences Cat#926-32213, "WB 1:15, 000"
- Donkey anti-Rabbit Alexa fluor 488, Thermofisher Cat#A-21206, IF 1:1000
- "Donkey anti-Mouse, Alexa Fluor 555", Thermofisher Cat#A-31570, IF 1:2000

Validation

Phospho-Histone H2AX, Cell Signaling Cat# 9718
This antibody was validated by detection of a single band at predicted molecular weight in cells exposure DNA damage and band which was absent in control samples https://www.cellsignal.com/products/primary-antibodies/phospho-histone-h2a-x-ser139-20e3-rabbit-mab/9718

Phospho-Chk2, Cell Signaling Cat# 2197
This antibody was validated by detection of a single band at predicted molecular weight in cells exposure DNA damage and band which was absent in control samples https://www.cellsignal.com/products/primary-antibodies/phospho-chk2-thr68-c13c1-rabbit-mab/2197

Phospho-ATM, Cell Signaling Cat# 5883
This antibody was validated by detection of a single band at predicted molecular weight in cells exposure DNA damage and band which was at significantly lower intensity in control samples https://www.cellsignal.com/products/primary-antibodies/phospho-atm-ser1981-d6h9-rabbit-mab/5883

Phospho-ATR, Cell Signaling Cat# 2853
This antibody was validated by detection of a single band at predicted molecular weight in cells exposure DNA damage and band which was at significantly lower intensity in control samples https://www.cellsignal.com/products/primary-antibodies/phospho-atr-ser428-antibody/2853

Phospho-STING (for mouse), Cell Signaling Cat# 72971, WB 1:1000
This antibody was validated by detection of a single band at predicted molecular weight in cells exposure cytosolic DNA and band which was absent in control samples
https://www.cellsignal.com/products/primary-antibodies/phospho-sting-ser365-d8f4w-rabbit-mab/72971

Phospho-STING (for human), Cell Signaling Cat# 19781
This antibody was validated by detection of a single band at predicted molecular weight in cells exposure cytosolic DNA and band which was absent in control samples.
https://www.cellsignal.com/products/primary-antibodies/phospho-sting-ser366-d7c3s-rabbit-mab/19781

STING, Cell Signaling Cat# 13647
This antibody was validated by detection of a single band (and in some cell lines two) at predicted molecular weights (https://www.cellsignal.com/products/primary-antibodies/sting-d2p2f-rabbit-mab/13647). This antibody was also validated in THP1 cells where the signal was absent in knockout cells (Fig. 2a)

Phospho-STAT2, Millipore-Sigma Cat# 07-224
This antibody was validated by detection of a single band at predicted molecular weights (https://www.emdmillipore.com/US/en/product/Anti-phospho-STAT2-Tyr689-Antibody, MM_NF-07-224). This antibody was also validated in THP1 cells where the signal was absent in shSTAT2 knockdown cells (Fig. 2e and Fig. S2d).

STAT2, Thermo Fisher Cat# 701105
This antibody was validated by detection of single band at predicted molecular weight (https://www.thermofisher.com/antibody/product/STAT2-Antibody-clone-1H10L19-Recombinant-Monoclonal/701105). This antibody was also validated in THP1 cells where the signal was absent in shSTAT2 knockdown cells (Fig. 2e and Fig. S2d).

Phospho-TBK1, Abcam Cat# ab109272
This antibody was validated by detection of single band at predicted molecular weight in cells treated with phosphatase inhibitor Calyculin A as well as with specific TBK1 phospho peptide as an epitope in immunodotblot (https://www.abcam.com/naktbk1-phospho-s172-antibody-epr28672-ab109272.html).

TBK1, Cell Signaling Cat# 3013
This antibody was validated by detection of a single band at predicted molecular weight. (https://www.cellsignal.com/products/primary-antibodies/tbk1-nak-antibody/3013). This antibody was also validated in THP1 cells where the signal was absent in shTBK1 knockdown cells (Fig. 2b, 3f, 3g).

Beta-Actin, Millipore-Sigma Cat# A2228-100UL
This antibody was validated by detection of a single band at predicted molecular weight (https://www.sigmaaldrich.com/US/en/product/sigma/a2228?context=product).

Tubulin, Millipore-Sigma Cat# T6199-200UL
This antibody was validated by detection of a single band at predicted molecular weight
(https://www.sigmaaldrich.com/US/en/product/sigma/t6199?context=product)

HA-Tag, Cell Signaling Cat# 2367
This antibody was validated by detection of a single band of HA-tagged protein at predicted molecular weight, No band was observed in sample without HA-tagged protein.
https://www.cellsignal.com/products/primary-antibodies/ha-tag-6e2-mouse-mab/2367

IRF3, Novus Biologicals Cat# NBP1-78769
This antibody was validated by detection of a single band at predicted molecular weight. https://www.novusbio.com/products/irf3-antibody_nbp1-78769
This antibody was also validated in IRF3–/– mouse embryonic fibroblast cells (Fig. 2c, and 3h).

Phospho-NF-kappa B p65, Cell Signaling Cat# 3033
This antibody was validated by detection of a single band at predicted molecular weight in cells specifically stimulated to induce NF-kappa B p65 phosphorylation
https://www.cellsignal.com/products/primary-antibodies/phospho-nf-kb-p65-ser536-93h1-rabbit-mab/3033

cGAS (for mouse), Cell Signaling Cat# 31659
This antibody was validated by detection of a single band at predicted molecular weight.
https://www.cellsignal.com/products/primary-antibodies/cgas-d3o8o-rabbit-mab-mouse-specific/31659
This antibody was also validated in cGAS–/– mouse embryonic fibroblast cells (Fig. 3a-b).

TBP, Abcam Cat# ab51841
This antibody was validated by detection of a single band at predicted molecular weight.
https://www.abcam.com/tata-binding-protein-tbp-antibody-mabcam-51841-chip-grade-ab51841.html

Histone H2A.X Antibody, Cell Signaling Cat# 2595
This antibody was validated by detection of a single band at predicted molecular weight.
https://www.cellsignal.com/products/primary-antibodies/histone-h2a-x-antibody/2595

Phospho-Rb (Ser807/811), Cell Signaling Cat# 8516
This antibody was validated by detection of a single band at predicted molecular weight in cells specifically stimulated to induce Rb phosphorylation
https://www.cellsignal.com/products/primary-antibodies/phospho-rb-ser807-811-d20b12-xp-rabbit-mab/8516

Phospho-Chk1 (Ser345), Cell Signaling Cat# 2348
This antibody was validated by detection of a single band at predicted molecular weight in cells specifically stimulated to induce Chk1 phosphorylation
https://www.cellsignal.com/products/primary-antibodies/phospho-chk1-ser345-133d3-rabbit-mab/2348

pADPr Antibody (10H), Santa Cruz Biotechnology Cat# sc-56198
This antibody was validated by detection of a characteristic smear-like big band on immunoblot, this band was absent in cells treated with PARP inhibitor in previous reports (PMID: 31519936 and PMID: 33144600). Another set of validation comes from our experiments where characteristic smear-like big band corresponding to PAR is present in cells stimulated with H2O2 (Fig 10b, d).

PARP (46D11) (For IP), Cell Signaling Cat# 9532
This antibody was validated by detection of a single band at predicted molecular weight and has been tested for IP

https://www.cellsignal.com/products/primary-antibodies/parp-46d11-rabbit-mab/9532

PARP1 Antibody (For WB), Cell Signaling Cat# 9542
This antibody was validated by detection of a single band at predicted molecular weight and has been tested for detection of cleaved product in response to staurosproin and etoposide.
https://www.cellsignal.com/products/primary-antibodies/parp-antibody/9542

PARP2 (For WB), Thermo Fisher Scientific Cat# MA5-34728
This antibody was validated by detection of a single band at predicted molecular weight
https://www.thermofisher.com/antibody/product/PARP2-Antibody-clone-JG34-56-Recombinant-Monoclonal/MA5-34728

Tankyrase-1 (For WB), Bethyl Laboratories, Inc., Cat# A302-399A-T
This antibody was validated by detection of a single band at predicted molecular weight
https://www.bethyl.com/product/A302-399A/Tankyrase+1+Antibody#

Anti-RAD 51 antibody, Bio-academia Cat# 70-001
This antibody was validated by detection of a single band at predicted molecular weight and has been tested for RAD51 foci formation by IF in response to X-ray irradiation.
https://www.bioacademia.co.jp/en/html/upload/save_image/E70-012%20anti-Rad51%20(human)antibody%20rabbit%20polyclonal.pdf

RPA70/RPA1 Antibody, Cell Signaling Cat# 2267
This antibody was validated by detection of a single band at predicted molecular weight and has been tested for RAD51 foci formation by IF in response to UV damage.
https://www.cellsignal.com/products/primary-antibodies/rpa70-rpa1-antibody/2267

Phospho-Histone H2AX, Cell Signaling Cat# 9718
This antibody was validated by detection of a single band at predicted molecular weight and has been tested for foci formation by IF in response to UV damage.
https://www.cellsignal.com/products/primary-antibodies/phospho-histone-h2a-x-ser139-20e3-rabbit-mab/9718

Anti-TBK1 Antibody, Atlas Antibodies Cat#HPA045797
This antibody (HPA045797) was validated for IF
https://www.atlasantibodies.com/products/antibodies/primary-antibodies/triple-a-polyclonals/tbk1-antibody-hpa045797/

HA-Tag, Cell Signaling Cat# 2367
This antibody was validated for IF in cells expressing HA-tagged protein. No signal was observed in cells lacking HA-tagged protein expression
https://www.cellsignal.com/products/primary-antibodies/ha-tag-6e2-mouse-mab/2367

Eukaryotic cell lines

Policy information about cell lines

Cell line source(s)	THP-1 (ATCC), U2OS (ATCC),LentiX-293T (Takara), HEK293 (ATCC), sources of other cells are described in the methods section.
Authentication	STR Analysis
Mycoplasma contamination	All cell lines tested mycoplasma negative.
Commonly misidentified lines (See ICLAC register)	No commonly misidentified cell lines were used.

Animals and other organisms

Policy information about studies involving animals; ARRIVE guidelines recommended for reporting animal research

Laboratory animals	Mouse embryonic fibroblasts (MEF) were isolated from Wild type (WT), cGAS−/−, cGAS(GS198AA), , Sting−/−, Irf3−/−, Ifnar1−/−, Stat2 −/−. Pregnant mice between ages of 10-20 weeks were euthanized, embryos were extracted (E11-13) and freshly dissected

	embryonic tissues were used to isolated primary MEF. Mice were co-housed in barrier animal facility in microisolator cages utilizing individually ventilated cage (IVC) systems with filtered air and active filter exhaust, 12-hour light/12 hour dark cycle in temperature and humidity controlled environment. All rodent diet was irradiated to be sterile which was provided ad libitum with a standard laboratory diet and sterile water was provided using automatic water systems
Wild animals	Starlet sea anemones:<br>Wild type, laboratory maintained, Self-sustaining Nematostella vectensis culture containing of 3-6 months old male and female animals (PMID: 17615350) was a kind gift from Timothy J Jegla (Penn State University Department of Biology). They were maintained in 6-well plates containing water with Instant Ocean® Aquarium Sea Salt (50gm/l) and fed brine shrimp. To stimulate them with cGAMP, N. vectensis were immersed in 0.5ml digitonin permeabilization solution (50mM Hepes pH 7.0, 100mM KCl, 85mM sucrose, 3mM MgCl2, 0.2% BSA, 1mM ATP, 0.1mM DTT, 2µg/ml digitonin) supplemented with 2µg cGAMP or vehicle for 10 minutes. Permeabilization solution was removed and the animals returned to maintenance sea salt water. Doxorubicin was added to the maintenance sea salt water to a final concentration of 2µM. 16hrs post treatment. N. vectensis were collected and washed once with cold PBS before lysing by the addition of 200µl RIPA buffer. Samples were homogenized by sonication and centrifuged at 4oC to remove debris. Soluble lysates were quantified for protein analysis by western blot.<br><br>Oysters:<br>Wild adult eastern oysters (Crassostrea virginica) of both male and female sexes ranging from 2cm - 7cm in length (1 to 3 years in age) were collected from a natural oyster reef on Virgnia's Eastern Shore. Following collection, the diploid oysters were placed on ice and transported approximately 3h to the University of Virginia main campus in Charlottesville, VA. There they were stored in in an aerated aquarium containing water supplemented with Instant Ocean® Aquarium Sea Salt (50gm/l). A small hole was made on the oyster shell beneath the adductor muscle where cGAMP (25µg/50gm oyster weight) or doxorubicin (1µg/50gm oyster weight) was injected. Treated oysters were kept at 4°C in the dark for 16hrs after which they were shucked, had their hemolymph/tissues harvested for analysis by western blotting, and dead oyster and shells were disposed of.
Field-collected samples	Study did not involve sample collected from the field
Ethics oversight	All animal experiments were approved by the University of Virginia's Institutional Animal Care and Use Committee.

Note that full information on the approval of the study protocol must also be provided in the manuscript.

Flow Cytometry

Plots

Confirm that:

- [x] The axis labels state the marker and fluorochrome used (e.g. CD4-FITC).
- [x] The axis scales are clearly visible. Include numbers along axes only for bottom left plot of group (a 'group' is an analysis of identical markers).
- [x] All plots are contour plots with outliers or pseudocolor plots.
- [x] A numerical value for number of cells or percentage (with statistics) is provided.

Methodology

Sample preparation	Primary mouse embryonic fibroblast (MEF) isolation:<br>Primary MEFs were isolated using Primary Mouse Embryonic Fibroblast Isolation Kit (Thermofisher, Cat# 88279) according to the manufacturer's instructions. Mouse embryos were extracted from a euthanized mouse (E11-13) and freshly dissected embryonic tissues were minced into 1-3mm3 fragments in ice cold HBSS buffer. Tissues were washed twice in cold HBSS buffer before 0.2ml MEF Isolation Enzyme (with Papain) was added to each tube. All samples were then incubated at 37°C for 30 minutes. The MEF Isolation Enzyme was removed and the tissues washed twice with cold HBSS buffer. The remaining products were resuspended in 0.5ml pre-warmed complete DMEM for subsequent Primary Cell Isolation by pipetting up and down. 1ml media was added to a single cell suspension which was then counted and tested for viability by trypan blue staining, and plated according to manufacturer’s protocol.<br><br>Starlet sea anemones:<br>Wild type, laboratory maintained, Self-sustaining Nematostella vectensis culture containing of 3-6 months old male and female animals (PMID: 17615350) was a kind gift from Timothy J Jegla (Penn State University Department of Biology). They were maintained in 6-well plates containing water with Instant Ocean® Aquarium Sea Salt (50gm/l) and fed brine shrimp. To stimulate them with cGAMP, N. vectensis were immersed in 0.5ml digitonin permeabilization solution (50mM Hepes pH 7.0, 100mM KCl, 85mM sucrose, 3mM MgCl2, 0.2% BSA, 1mM ATP, 0.1mM DTT, 2µg/ml digitonin) supplemented with 2µg cGAMP or vehicle for 10 minutes. Permeabilization solution was removed and the animals returned to maintenance sea salt water. Doxorubicin was added to the maintenance sea salt water to a final concentration of 2µM. 16hrs post treatment. N. vectensis were collected and washed once with cold PBS before lysing by the addition of 200µl RIPA buffer. Samples were homogenized by sonication and centrifuged at 4oC to remove debris. Soluble lysates were quantified for protein analysis by western blot.<br><br>Oysters:<br>Wild adult eastern oysters (Crassostrea virginica) of both male and female sexes ranging from 2cm - 7cm in length (1 to 3 years in age) were collected from a natural oyster reef on Virgnia's Eastern Shore. Following collection, the diploid oysters were placed on ice and transported approximately 3h to the University of Virginia main campus in Charlottesville, VA. There they were stored in in an aerated aquarium containing water supplemented with Instant Ocean® Aquarium Sea Salt (50gm/l). A small hole was made on the oyster shell beneath the adductor muscle where cGAMP (25µg/50gm oyster weight) or

	doxorubicin (1µg/50gm oyster weight) was injected. Treated oysters were kept at 4°C in the dark for 16hrs after which they were shucked and had their hemolymph/tissues harvested for analysis by western blotting.
Instrument	Flow cytometer (Attune NxT), WesternBlot (Odyssey CLx, Licor), qPCR(QuantstudioFlex 7, ThermoFisfer Scientific) and All images were obtained by confocal microscope system (Nikon, C2).
Software	GraphPad Prism 7 software, Microsoft Excel, OpenComet v1.3 software (in ImageJ), FCS Express and Multicycle AV plugin (Phoenix Flow Systems), online tool MUSCLE (EBI), CRISPResso, LICOR image studio, PhyloT
Cell population abundance	100%
Gating strategy	For Cell cycle material using BrDU and 7-AAD, we used the below methods:<br>~10,000-30,000 cells were acquired on an Attune NxT cytometer and analyzed using FCS Express software. Cell cycle analysis was performed on BrDU and 7-AAD stained cells and single cell events were determined by gating on the area against the width of the 7-AAD pulse signal. Gating for S-phase, BrDU-positive populations of cell cycle was determined using Fluorescence Minus One (FMO) controls. G1 and G2 populations were determined from untreated, normal cycling cells gated on single cell events.<br><br>For experiments using Propidium Iodide based cell cycle analysis we used the below methods:<br>~10,000-30,000 cells were acquired on an Attune NxT cytometer and analyzed using FCS Express software. Cell cycle analysis was performed on propidium iodide stained cells and single cell events were determined by gating on the area against the width of the propidium iodide pulse signal. DNA cell cycle modeling and fit was performed using the Multicycle AV plugin (Phoenix Flow Systems).<br><br>For experiments using GFP and mCherry, we used the below methods:<br>~10,000-30,000 cells were acquired on an Attune NxT cytometer and analyzed using FCS Express software. Single cell events were determined by gating on the area against the width of the forward scatter pulse signal. Debris removal was performed by excluding events with very low forward and side scatter. For determining % of GFP and mCherry positive cells we established positive and negative populations using mock transfections as negative controls and GFP or mCherry transfected samples as Fluorescence Minus One controls.

☒ Tick this box to confirm that a figure exemplifying the gating strategy is provided in the Supplementary Information.
